# Supplementary material for: MathIOmica: An Integrative Platform for Dynamic Omics
Source: Sci Rep. 2016 Nov 24;6:37237. doi: 10.1038/srep37237 (PMC5121649; doi:10.1038/srep37237)
Supplement: Supplementary Note 1 [file srep37237-s1.pdf]

MathIOmica: An Integrative Platform for Dynamic Omics

George I. Mias[1,*], Tahir Yusufaly[2], Raeuf Roushangar[1], Lavida R. K. Brooks[1], Vikas V. Singh[1], and Christina Christou[3]

[1]Michigan State University, Biochemistry and Molecular Biology, East Lansing, MI 48824, USA
[2]University of Southern California, Department of Physics and Astronomy, Los Angeles, CA, 90089, USA
[3]Mercy Cancer Center, Department of Radiation Oncology, Mason City, IA 50401, USA
[*]gmias@msu.edu

SUPPLEMENTARY NOTE 1

MathIOmica: Omics Analysis Tutorial

- Loading the MathIOmica Package
- Data in MathIOmica
- Transcriptome Data
- Proteomic Data
- Metabolomic Data
- Combined Data Clustering
- Visualization
- Annotation and Enrichment

MathIOmica is an omics analysis package designed to facilitate method development for the analysis of multiple omics in Mathematica, particularly for dynamics (time series/longitudinal data). This extensive tutorial follows the analysis of multiple dynamic omics data (transcriptomics, proteomics, and metabolomics from human samples). Various MathIOmica functions are introduced in the tutorial, including additional discussion of related functionality. We should note that the approach methods are simply an illustration of MathIOmica functionality, and should not be considered as a definitive appoach. Additionally, certain details are included to illustrate common complications (e.g. renaming samples, combining datasets, transforming accessions from one database to another, dealing with replicates and Missing data, etc.).

After a brief discussion of data in MathIOmica, each example data (transcriptome, proteome and metabolome) are imported and preprocessed. Next a simulation is carried out to obtain datasets for each omics used to assess statistical significance cutoffs. The datasets are combined, and classified for time series patterns, followed by clustering. The clusters are visualized, and biological annotation of Gene Ontology (GO) and pathway analysis (KEGG: Kyoto Encyclopedia of Genes and Genomes) are finally considered.

N.B.1 For a more streamlined/simple example with less discussion please check out the tutorial on MathIOmica Dynamic Transcriptome.

N.B.2 We highly recommend the saving of intermediate results whenever possible. Some functions perform lengthly intensive computations and the performance may vary from system to system. Please use `Put` to save expressions to a file, and equivalently `Get` to recover these expressions.

Loading the MathIOmica Package

The functions defined in the `MathIOmica`` context provide support for conducting analyses of omics data (See also the MathIOmica Overview).

This loads the package:

```
In[1]:= << MathIOmica`
```

Also we can load MathIOmica as:

```
In[1]:= Needs["MathIOmica`"]
```

Data in MathIOmica

In this section we will discuss the data objects in use by MathIOmica, particularly the format of an OmicsObject. The data in the tutorial will be imported as an OmicsObject which is first described in this section. Then we present the example data included with MathIOmica. The example data will be imported in subsequent sections to illustrate analysis methods available in MathIOmica.

Data Format: OmicsObject

In MathIOmica the calculations utilize what we term an omics object (OmicsObject). An OmicsObject is an association of associations with some additional characteristics. It has an external (outer) association to denote samples and an internal (inner) association for annotation.

OmicsObject Structure

In an OmicsObject the outer association has M outer labels as keys, corresponding to M samples. Across the samples there are N inner labels (e.g. identifiers for genes/proteins), and the the inner labels are the same across samples. For a given j^{th} outer label, $OuterLabel_j$, the kth inner label, $InnerLabel_k$ has a value of :

$InnerLabel_k \rightarrow \{\{Measurements_{jk}\}, \{Metadata_{jk}\}\}$

OmicsObject structure:

```
<|OuterLabel_1 → <|InnerLabel_1 → {{Measurements_11}, {Metadata_11}},
   InnerLabel_2 → {{Measurements_12}, {Metadata_12}},
   InnerLabel_3 → {{Measurements_13}, {Metadata_13}},
   ...,
   InnerLabel_k → {{Measurements_1k}, {Metadata_1k}},
   ...,
   InnerLabel_N → {{Measurements_1N}, {Metadata_1N}}|>,
 OuterLabel_2 → <|InnerLabel_1 → {{Measurements_21}, {Metadata_21}},
   InnerLabel_2 → {{Measurements_22}, {Metadata_22}},
   InnerLabel_3 → {{Measurements_23}, {Metadata_23}},
   ...,
   InnerLabel_k → {{Measurements_2k}, {Metadata_2k}},
   ...,
   InnerLabel_N → {{Measurements_2N}, {Metadata_2N}}|>,
 ...,
 OuterLabel_j → <|InnerLabel_1 → {{Measurements_j1}, {Metadata_j1}},
   InnerLabel_2 → {{Measurements_j2}, {Metadata_j2}},
   InnerLabel_3 → {{Measurements_j3}, {Metadata_j3}},
   ...,
   InnerLabel_k → {{Measurements_jk}, {Metadata_jk}},
   ...,
   InnerLabel_N → {{Measurements_jN}, {Metadata_jN}}|>,
 ...,
 OuterLabel_M → <|InnerLabel_1 → {{Measurements_M1}, {Metadata_M1}},
   InnerLabel_2 → {{Measurements_M2}, {Metadata_M2}},
   InnerLabel_3 → {{Measurements_M3}, {Metadata_M3}},
   ...,
   InnerLabel_k → {{Measurements_Mk}, {Metadata_Mk}},
   ...,
   InnerLabel_N → {{Measurements_MN}, {Metadata_MN}}|>
|>
```

For any j^{th} outer label, $OuterLabel_j$, it is possible that the mth inner label, $InnerLabel_m$ is missing and takes a Missing[] value in the form $InnerLabel_m \rightarrow$ Missing[]. This can happen if the measurement was not performed for the sample, or no value was recorded (e.g. mass sectrometry data).

For example here is a list of 3 samples using protein identifiers (specifically, these are UniProt accessions). The measurements are relative intensities in this case and the metadata is the number of peptides per sample.

```
In[2]:= omicsObjectExample = <|"FirstSample" → <|{"A0AVT1"} → {{0.937}, {17}}, {"A0MZ66"} → {{1.059}, {9}},
      {"A1A4S6"} → {{1.03}, {11}}, {"A1L0T0"} → {{1.268}, {4}}, {"A0FGR8"} → Missing[] |>,
    "SecondSample" → <|{"A0AVT1"} → {{1.003}, {17}}, {"A0MZ66"} → Missing[],
      {"A1A4S6"} → {{0.779}, {11}}, {"A1L0T0"} → {{0.917}, {4}}, {"A0FGR8"} → {{0.921}, {24}} |>,
    "ThirdSample" → <|{"A0AVT1"} → {{1.064}, {19}}, {"A0MZ66"} → Missing[],
      {"A1A4S6"} → {{0.545}, {5}}, {"A1L0T0"} → Missing[], {"A0FGR8"} → {{0.87}, {23}} |> |>;
```

The outer labels of an OmicsObject are strings, while the inner labels are typically lists of strings.

Methods to Import Data as an OmicsObject

There are multiple methods to import data as an OmicsObject using MathIOmica. Four functions assist with importing data directly from text files:

(i) `DataImporter` provides a graphical dynamic interface that utilizes file headers to assist with the creation of OmicsObject variables from multiple files.

(ii) The `OmicsObjectCreator` function provides a function to create an OmicsObject from already existing/imported data in a Mathematica notebook.

(iii) `DataImporterDirect` and (iv) `DataImporterDirectLabeled` provide additional expert mode functions that may be used to directly import data as OmicsObject variables without a graphical interface.

`DataImporter`[*associationName*]	provides a graphical interface to extract data and create an OmicsObject variable *associationName* for associations of information.
`OmicsObjectCreator`[*outerLabels*, *innerLabels*, *measurements*,*metadata*]	creates an `OmicsObject` for use with MathIOmica. It uses the following inputs:<br>*outerLabels* — Outer labels (keys) for the OmicsObject.<br>*innerLabels* — Inner labels (keys) for identifiers in the OmicsObject.<br>*measurements* — List of measurements for each inner label.<br>*metadata* — List of metadata for each label.
`DataImporterDirect`[*positionsList*, *fileList*, *headerLines*]	*Expert Usage:* The `DataImporterDirect` function is a helper function originally created for `DataImporter`.<br>`DataImporterDirect`[*positionsList*, *fileList*, *headerLines*] creates an OmicsObject importing the column number in *positionsList* from the *fileList* file path list, and importing data by skipping a number of *headerLines*.
`DataImporterDirectLabeled`[*sampleRules*, *fileList*, *headerLines*, *headerColumnAssociations*]	*Expert Usage:* The `DataImporterDirectLabeled` function creates an `OmicsObject` association for *variableName*, by imporing data from the files at the paths specified in the *fileList*, using the *sampleRules* as a label to column header imported rule for each file, and the *headerColumnAssociations* list of associations to associate column headers to column positions for each file.

Functions for importing/creating OmicsObject datasets.

Working with OmicsObject Data

An OmicsObject is an association of associations, and so Query can be used directly to access and manipulate components. MathIOmica also offers multiple functions that can implement computations and manipulation of an OmicsObject:

`Applier`[*function*, *inputData*]	applies *function* to OmicsObject, association or list *inputData* components.
`ApplierList`[*function*, *inputData*]	applies *function* to list of lists from an association, nested association or components or a matrix *inputData*.
`ConstantAssociator`[*inputAssociation*, *associationAddition*]	adds multi key constant to an OmicsObject (or an association of associations) *inputAssociation*, with each addition specified in a single association *associationAddition*, of form <|addition1→ Value1,addition2→ Value2,...|>.
`CreateTimeSeries`[*dataIn*]	creates a time series list across an OmicsObject *dataIn* using outer Keys for points.
`EnlargeInnerAssociation`[*omicsObjectList*]	combines a list of OmicsObject (associations of associations) *omicsObjectList* elements by enlarging the inner associations – inner association Keys must be different.
`EnlargeOuterAssociation`[*omicsObjectList*]	combines a list, *omicsObjectList*, of OmicsObject (or associations of associations) elements to a combined output by enlarging the outer associations – outer association keys must be different.
`FilteringFunction`[*omicsObject*,*cutoff*]	filters an OmicsObject data by a chosen comparison (by default greatr or equal) to a *cutoff*.
`FilterMissing`[*omicsObject*, *percentage*]	filters out data from *omicsObject* if across the datasets a *percentage* of data points is missing.
`LowValueTag`[*omicsObject*, *valueCutoff*]	takes an *omicsObject* and tags values in specified position as `Missing[]` based on provided *valueCutoff*.
`MeasurementApplier`[*function*,*omicsObject*]	applies a *function* to the measurement list of an *omicsObject*, ignoring missing values.
`Returner`[*originalAssociation*, *update*]	returns a modified *originalAssociation* updated at a specified position by the single association *update*, e.g. from `Applier` or `ApplierList` result.

Functions for manipuling OmicsObject datasets.

Example Data

MathIOmica comes with multiple example data. The data can be found in the `ConstantMathIOmicaExamplesDirectory`:

We can get a listing of the current example Data by evaluating:

```
In[3]:= FileNames[__, ConstantMathIOmicaExamplesDirectory]
```

The data contains both initial (raw) data and additionally intermediate data that have been analyzed in MathIOmica and are used in the examples (***N.B.*** *these files should* ***not*** *be altered or removed*). The dynamic raw datasets are from an integrative Personal Omics Profile as described below:

**integrative Personal Omics Profiling (iPOP)**	Data from the first integrative Omics Profiling (iPOP) is used comprised of dynamics from proteomics transcriptomics and metabolomics. The data corresponds to a time series analysis of omics from blood com-ponenets from a single individual.<br>Different samples (from 7 to 21 included here) were obtained at different time points. The time points included here correspond to days ranging from 186th to the 400th day of the study, (this can be repre-sented in the following sample to day association: `<|7→186,8→255,9→289,10→290,11→292,12→294,13→297,14→301,15→307,16→311,17→322,18→329,19→369,20→380,21→400|>`. On day 289 the subject of the study had a Respiratory syncytial virus infection. Additionally, after day 301, the subject displayed high glucose levels and was eventually diagnosed with type 2 diabetes. The analyzed mapped data are used in [illegible]

Example iPOP Set Description	*File Names located in the* ***ConstantMathIOmicaExamplesDirectory***.
**iPOP Transcriptome.** The transcriptomic data included was obtained from mapping of the originally RNA Sequencing raw data using the Tuxedo suite. The data corresponds to transcriptome from peripheral blood mononuclear cells (PBMCs).	iPOP_ 07_genes.fpkm_tracking<br>iPOP_ 08_genes.fpkm_tracking<br>iPOP_ 09_genes.fpkm_tracking<br>iPOP_ 10_genes.fpkm_tracking<br>iPOP_ 11_genes.fpkm_tracking<br>iPOP_ 12_genes.fpkm_tracking<br>iPOP_ 13_genes.fpkm_tracking<br>iPOP_ 14_genes.fpkm_tracking<br>iPOP_ 15_genes.fpkm_tracking<br>iPOP_ 16_genes.fpkm_tracking<br>iPOP_ 17_genes.fpkm_tracking<br>iPOP_ 18_genes.fpkm_tracking<br>iPOP_ 19_genes.fpkm_tracking<br>iPOP_ 20_genes.fpkm_tracking<br>iPOP_ 21_genes.fpkm_tracking
**iPOP Proteome.** The Proteomics data from analysis of mass spectrometry data using the Sequest algorithm implemented by ProteomeDiscoverer. The data corresponds to proteome from PBMCs.<br>The names of the files provide a correspondce of samples to Tandem Mass Tag labels in order of increasing m/z values from 126 to 131 amu. 6 TMT labels were used in each experiment.<br>The data has been adapted from the original to UniProt accessions.	8_7_9_10_11_14_MulticonsensusReports_3Replicates.csv<br>8_12_13_15_16_14_MulticonsensusReports_3Replicates.csv<br>8_17_19_20_21_14_MulticonsensusReports_3Replicates.csv
**iPOP Metabolome.** The Metabolomics data from analysis of mass spectrometry data. The data corresponds to small molecule metabolomics from plasma ran with technical triplicates.<br>The names of the files provide a correspondce of samples ran in positive or negative mode.	metabolomics_negative_mode.csv<br>metabolomics_positive_mode.csv

Description of Example iPOP original datasets and corresponding files in the `ConstantMathIOmicaExamplesDirectory`. **N.B.** this table is provided as a reference for the examples, and these files should ***not*** be altered or removed.

Various analyzed datasets are used in the MathIOmica documentation for examples:

Data Description	*File Name(s) located in the* ***ConstantMathIOmicaExamplesDirectory*** *.*
iPOP transcriptome imported as an OmicsObject across all timepoints.	rnaExample
iPOP proteome data imported as an OmicsObject across all timepoints.	proteinExample
iPOP metabolome data imported as an OmicsObject across all timepoints and technical replicates for negative and positive mode aligned mass spectrometry features.	metabolomicsNegativeModeExample<br>metabolomicsPositiveModeExample
Example time series from proteomics.	proteinTimeSeriesExample
Example classification results from proteomics.	proteinClassificationExample
Example classification results from proteomics.	proteinClusteringExample
Example combined clustering results from transcriptome, proteome and metabolome data.	combinedClustersExample
Example enrichment analysis results for Gene Ontology and KEGG pathway analysis for combined omics data in this tutorial.	combinedGOAnalysis<br>combinedKEGGAnalysis
Spectra from proteomics mass spectrometry data examples.	small.pwiz.1.1.mzML<br>exampleMS3.mzXML

Description of example analyzed datasets and corresponding files in the ConstantMathIOmicaExamplesDirectory . **N.B.** this table is provided as a reference for the examples, and these files should ***not*** be altered or removed.

Transcriptome Data

In this section we import the example transcriptome iPOP dataset, and illustrate a preprocessing approach for this omic dataset.

Importing OmicsObject Transcriptome Data

We first import the transcriptomics data example (for details on how to import such data please refer to DataImporter , DataImporterDirect , DataImporterDirectLabeled and OmicsObjectCreator documentation).

We import the transcriptomics OmicsObject

```
In[4]:= rnaExample = Get[FileNameJoin[{ConstantMathIOmicaExamplesDirectory, "rnaExample"}]]
```

```
Out[4]= <|7 → <|{FAM138A, RNA} → {{0}, {OK}}, {OR4F5, RNA} → {{0}, {OK}},
    {LOC729737, RNA} → {{2.73998}, {OK}}, ··· 25 262 ···, {LOC100507412, RNA} → {{0}, {OK}},
    {RNA45S5, RNA} → {{0}, {OK}}, {DUX4L, RNA} → {{0}, {OK}}|>,
  8 → <|··· 1 ···|>, ··· 11 ···, 20 → <|··· 1 ···|>, 21 → <|··· 1 ···|>|>

large output | show less | show more | show all | set size limit...
```

There are multiple samples given by the outer associations. We can use Query to get any data. For example we can get the outer keys:

```
In[5]:= Query[Keys]@rnaExample
Out[5]= {7, 8, 9, 10, 11, 12, 13, 14, 15, 16, 17, 18, 19, 20, 21}
```

Notice that we have used "@" to form a Query using a prefix function application, which is used throughout the MathIOmica tutorials and documentation. This is the same as using the [ ] form:

```
In[6]:= Query[Keys][rnaExample]
Out[6]= {7, 8, 9, 10, 11, 12, 13, 14, 15, 16, 17, 18, 19, 20, 21}
```

We can get the expression raw data from any sample and entry. For example, the 10th and 14th entries in sample 12:

```
In[7]:= Query["12", {7777, 55}]@rnaExample
Out[7]= <|{NDNL2, RNA} → {{21.1197}, {OK}}, {ATAD3C, RNA} → {{0.560212}, {OK}}|>
```

The keys correspond to "Gene Symbols" and are also tagged with an "RNA" label. The values of all the keys/IDs correspond to {{measurements}, {metadata}}, and in this particular example {{"FPKM" values}, {"FPKM status"}}. Here, FPKM stands for Fragments Per Kilobase of transcript per Million mapped reads. The example is from mapped RNA-Sequencing data. FPKM is then a relative measure of transcript (gene) expression.

We can query all timepoints for a particular gene of interest if it exists. We must use the same labels as the actual keys of the OmicsObject:

```
In[8]:= Query[All, Key@{"NFKBIB", "RNA"}]@rnaExample
Out[8]= <|7 → {{12.7644}, {OK}}, 8 → {{14.9997}, {OK}}, 9 → {{15.8482}, {OK}},
 10 → {{17.3504}, {OK}}, 11 → {{18.5309}, {OK}}, 12 → {{16.7081}, {OK}}, 13 → {{14.6549}, {OK}},
 14 → {{17.3951}, {OK}}, 15 → {{8.93065}, {OK}}, 16 → {{16.2545}, {OK}}, 17 → {{17.9217}, {OK}},
 18 → {{16.0331}, {OK}}, 19 → {{18.7293}, {OK}}, 20 → {{10.8115}, {OK}}, 21 → {{12.9051}, {OK}}|>
```

We note that we added Key@ before the bracket to indicate that this list is used as a key for the inner associations.

We can query all timepoints for multiple genes of interest if it exists. We must use the same labels as the actual keys of the OmicsObject:

```
In[9]:= Query[All, {Key@{"NFKBIB", "RNA"}, Key@{"NDNL2", "RNA"}}]@rnaExample
Out[9]= <|7 → <|{NFKBIB, RNA} → {{12.7644}, {OK}}, {NDNL2, RNA} → {{13.6201}, {OK}}|>,
 8 → <|{NFKBIB, RNA} → {{14.9997}, {OK}}, {NDNL2, RNA} → {{16.3813}, {OK}}|>,
 9 → <|{NFKBIB, RNA} → {{15.8482}, {OK}}, {NDNL2, RNA} → {{16.2763}, {OK}}|>,
 10 → <|{NFKBIB, RNA} → {{17.3504}, {OK}}, {NDNL2, RNA} → {{17.2483}, {OK}}|>,
 11 → <|{NFKBIB, RNA} → {{18.5309}, {OK}}, {NDNL2, RNA} → {{18.3254}, {OK}}|>,
 12 → <|{NFKBIB, RNA} → {{16.7081}, {OK}}, {NDNL2, RNA} → {{21.1197}, {OK}}|>,
 13 → <|{NFKBIB, RNA} → {{14.6549}, {OK}}, {NDNL2, RNA} → {{22.0412}, {OK}}|>,
 14 → <|{NFKBIB, RNA} → {{17.3951}, {OK}}, {NDNL2, RNA} → {{17.1224}, {OK}}|>,
 15 → <|{NFKBIB, RNA} → {{8.93065}, {OK}}, {NDNL2, RNA} → {{10.4774}, {OK}}|>,
 16 → <|{NFKBIB, RNA} → {{16.2545}, {OK}}, {NDNL2, RNA} → {{23.6771}, {OK}}|>,
 17 → <|{NFKBIB, RNA} → {{17.9217}, {OK}}, {NDNL2, RNA} → {{21.8782}, {OK}}|>,
 18 → <|{NFKBIB, RNA} → {{16.0331}, {OK}}, {NDNL2, RNA} → {{21.4414}, {OK}}|>,
 19 → <|{NFKBIB, RNA} → {{18.7293}, {OK}}, {NDNL2, RNA} → {{19.9134}, {OK}}|>,
 20 → <|{NFKBIB, RNA} → {{10.8115}, {OK}}, {NDNL2, RNA} → {{22.5756}, {OK}}|>,
 21 → <|{NFKBIB, RNA} → {{12.9051}, {OK}}, {NDNL2, RNA} → {{22.55}, {OK}}|>|>
```

Or in a more concise form

```
In[10]:= Query[All, Key[#] & /@ {{"NFKBIB", "RNA"}, {"NDNL2", "RNA"}}]@rnaExample

Out[10]= <|7 → <|{NFKBIB, RNA} → {{12.7644}, {OK}}, {NDNL2, RNA} → {{13.6201}, {OK}}|>,
 8 → <|{NFKBIB, RNA} → {{14.9997}, {OK}}, {NDNL2, RNA} → {{16.3813}, {OK}}|>,
 9 → <|{NFKBIB, RNA} → {{15.8482}, {OK}}, {NDNL2, RNA} → {{16.2763}, {OK}}|>,
 10 → <|{NFKBIB, RNA} → {{17.3504}, {OK}}, {NDNL2, RNA} → {{17.2483}, {OK}}|>,
 11 → <|{NFKBIB, RNA} → {{18.5309}, {OK}}, {NDNL2, RNA} → {{18.3254}, {OK}}|>,
 12 → <|{NFKBIB, RNA} → {{16.7081}, {OK}}, {NDNL2, RNA} → {{21.1197}, {OK}}|>,
 13 → <|{NFKBIB, RNA} → {{14.6549}, {OK}}, {NDNL2, RNA} → {{22.0412}, {OK}}|>,
 14 → <|{NFKBIB, RNA} → {{17.3951}, {OK}}, {NDNL2, RNA} → {{17.1224}, {OK}}|>,
 15 → <|{NFKBIB, RNA} → {{8.93065}, {OK}}, {NDNL2, RNA} → {{10.4774}, {OK}}|>,
 16 → <|{NFKBIB, RNA} → {{16.2545}, {OK}}, {NDNL2, RNA} → {{23.6771}, {OK}}|>,
 17 → <|{NFKBIB, RNA} → {{17.9217}, {OK}}, {NDNL2, RNA} → {{21.8782}, {OK}}|>,
 18 → <|{NFKBIB, RNA} → {{16.0331}, {OK}}, {NDNL2, RNA} → {{21.4414}, {OK}}|>,
 19 → <|{NFKBIB, RNA} → {{18.7293}, {OK}}, {NDNL2, RNA} → {{19.9134}, {OK}}|>,
 20 → <|{NFKBIB, RNA} → {{10.8115}, {OK}}, {NDNL2, RNA} → {{22.5756}, {OK}}|>,
 21 → <|{NFKBIB, RNA} → {{12.9051}, {OK}}, {NDNL2, RNA} → {{22.55}, {OK}}|>|>
```

We should also note that we can take advantage of Mathematica's native direct access to Wolfram Alpha, to look up any "Gene Symbol" information by evaluating (needs a network connection):

NFKBIB

Processing Transcriptome Mapped Data

We will next preprocess the imported transcriptome data. We will first relabel the data, carry out quantile normalization and filtering and we will finally create time series.

Labeling, Normalization and Filtering

Re-labeling Samples with Times

First, we illustrate how to change the outer keys. In this example, we notice that the sample numberings do not correspond to actual days, so we may want to adjust the outer keys to correspond to real times.

We form an association between samples to actual days of the study:

```
In[12]:= sampleToDays =
  <|"7" → "186", "8" → "255", "9" → "289", "10" → "290", "11" → "292", "12" → "294", "13" → "297", "14" → "301",
   "15" → "307", "16" → "311", "17" → "322", "18" → "329", "19" → "369", "20" → "380", "21" → "400"|>;
```

We can now do a KeyMap to rename the outer keys:

```
In[13]:= rnaLongitudinal = KeyMap[sampleToDays, rnaExample]

Out[13]= <|186 → <|{FAM138A, RNA} → {{0}, {OK}},
     {OR4F5, RNA} → {{0}, {OK}}, {LOC729737, RNA} → {{2.73998}, {OK}}, ⋯ 25262 ⋯,
     {LOC100507412, RNA} → {{0}, {OK}}, {RNA45S5, RNA} → {{0}, {OK}}, {DUX4L, RNA} → {{0}, {OK}}|>,
   255 → <| ⋯ 1 ⋯ |>, ⋯ 11 ⋯, 380 → ⋯ 1 ⋯, 400 → <| ⋯ 1 ⋯ |>|>

large output | show less | show more | show all | set size limit...
```

Quantile Normalization

QuantileNormalization[*data*]	performs quantile normalization of *data*.

QuantileNormalization can perform quantile normalization across various samples for multiple forms of data, including OmicsObject and matrix data.

We normalize the transcriptome data using the QuantileNormalization function.

In[14]:=
```
rnaQuantileNormed = QuantileNormalization[rnaLongitudinal]
```

Out[14]=
```
<|186 → <|{FAM138A, RNA} → {{0.}, {OK}}, {OR4F5, RNA} → {{0.}, {OK}},
  {LOC729737, RNA} → {{2.73998}, {OK}}, ⋯25262⋯, {LOC100507412, RNA} → {{0.}, {OK}},
  {RNA45S5, RNA} → {{0.}, {OK}}, {DUX4L, RNA} → {{0.}, {OK}}|>, ⋯13⋯, 400 → <|⋯1⋯|>|>
```
large output | show less | show more | show all | set size limit...

Tag Missing and Low Values

Next, we will tag values of less than 1 FPKM as Missing. Additionally, we will treat values of FPKM less than 5 as "noise" and set them all to a token value of 1.

LowValueTag[*omicsObject*, *valueCutoff*]	takes an *omicsObject* and tags values in specified position as Missing[] based on provided *valueCutoff*.

LowValueTag allows us to tag low values.

option name	default value	
ComponentIndex	1	Selection of which component of a list to use in the association or OmicsObject input values.
ListIndex	1	Selection of which list to use in the association or OmicsObject input values.
OtherReplacement	_Missing :> Missing[]	Replacement rule for any other kind of replacement in the data.
ValueReplacement	Missing[]	Value that specifies how tagged data points will be replaced.

Options for LowValueTag.

We first use LowValueTag to tag values of 0 as Missing[]:

In[15]:=
```
rnaZeroTagged = LowValueTag[rnaQuantileNormed, 0]
```

Out[15]=
```
<|186 → <|{FAM138A, RNA} → {{Missing[]}, {OK}},
  {OR4F5, RNA} → {{Missing[]}, {OK}}, {LOC729737, RNA} → {{2.73998}, {OK}}, ⋯25263⋯,
  {RNA45S5, RNA} → {{Missing[]}, {OK}}, {DUX4L, RNA} → {{Missing[]}, {OK}}|>,
 255 → <|⋯1⋯|>, ⋯11⋯, 380 → <|⋯1⋯|>, 400 → <|⋯1⋯|>|>
```
large output | show less | show more | show all | set size limit...

We next use LowValueTag again to set all FPKM values <1 to unity:

```
In[16]:= rnaNoiseAdjusted = LowValueTag[rnaZeroTagged, 1, ValueReplacement → 1]

Out[16]= <|186 → <|{FAM138A, RNA} → {{Missing[]}, {OK}},
    {OR4F5, RNA} → {{Missing[]}, {OK}}, {LOC729737, RNA} → {{2.73998}, {OK}}, ⋯25263⋯,
    {RNA45S5, RNA} → {{Missing[]}, {OK}}, {DUX4L, RNA} → {{Missing[]}, {OK}}|>,
  255 → <|⋯1⋯|>, ⋯11⋯, 380 → <|⋯1⋯|>, 400 → <|⋯1⋯|>|>

large output | show less | show more | show all | set size limit...
```

Filter Data

We will next remove values that have been tagged as Missing[], retaining data that have at least 3/4 data points available across all samples. Here we use the function FilterMissing:

FilterMissing[*omicsObject*, *percentage*]	filters out data from *omicsObject*, retaining data across the datasets with a *percentage* of data points not missing.

FilterMissing allows the removal of data marked as Missing[], and retains only data with measurements available for a certain percentage of samples.

option name	default value	
MininumPoints	3	Minimum number of datapoints to keep.
Reference	{}	Select a reference outer key for which should remove dataset if the reference point has a Missing value.
ShowPlots	True	Whether to show summary plots.

Options for FilterMissing.

In this dataset we will use a reference point, day "255" which was a healthy measurement.

Hence, we filter out data where the reference point "255" is missing and retain data with at least 3/4 poings available:

```
In[17]:= rnaFiltered = FilterMissing[rnaNoiseAdjusted, 3/4, Reference → "255"]
```

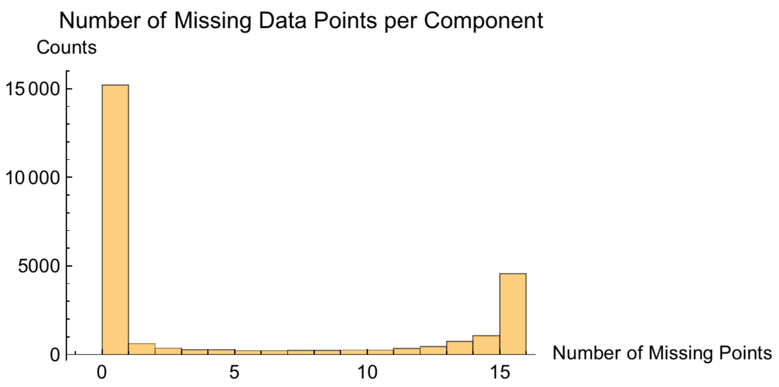

{Missing -> Counts: ,
<|0 → 15 203, 1 → 615, 2 → 365, 3 → 279, 4 → 276, 5 → 217, 6 → 217, 7 → 231, 8 → 238,
9 → 248, 10 → 255, 11 → 334, 12 → 440, 13 → 737, 14 → 1058, 15 → 4555|>}

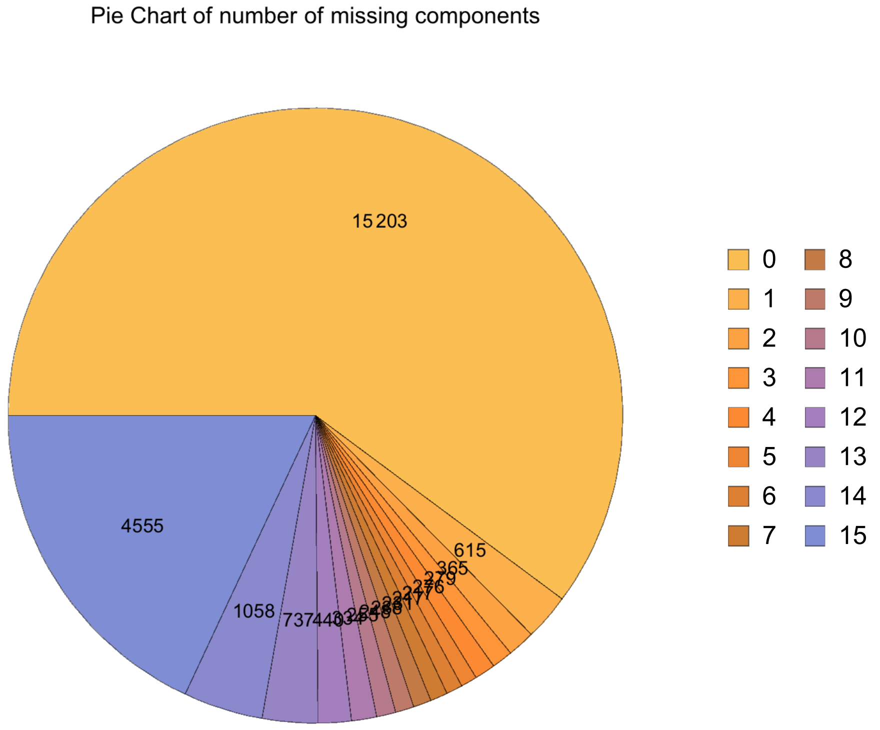

Out[17]=

```
<|186 → <|{LOC729737, RNA} → {{2.73998}, {OK}}, {DDX11L1, RNA} → {{6.75461}, {OK}},
   {WASH7P, RNA} → {{11.8883}, {OK}}, … 16 456 …, {LOC101929148, RNA} → {{1}, {OK}},
   {KDM5D, RNA} → {{7.73125}, {OK}}, {UTY, RNA} → {{3.16532}, {OK}}|>, … 13 …, 400 → <| … 1 … |>|>
```

large output | show less | show more | show all | set size limit...

Create Transcriptome Time Series

We can now create time series for each of the genes. MathIOmica provides functions to facilitate the process, such as `CreateTimeSeries` and `TimeExtractor`. The functions assume an OmicsObject as an input for which times have been used as the sample labels (outer keys).

`CreateTimeSeries[omicsObject]`	creates a time series list across an OmicsObject using outer keys as times.
`TimeExtractor[omicsObject]`	extracts a list of sorted times from an OmicObject's outer keys.

We extract the times for the filtered RNA data using `TimeExtractor`:

```
In[18]:= timesRNA = TimeExtractor[rnaFiltered]
Out[18]= {186, 255, 289, 290, 292, 294, 297, 301, 307, 311, 322, 329, 369, 380, 400}
```

For each gene we now extract a time series (list of values) corresponding to these times:

```
In[19]:= timeSeriesRNA = CreateTimeSeries[rnaFiltered]
Out[19]= <|{LOC729737, RNA} → {2.73998, 1, 5.15563, 4.53362,
   5.71829, 1, 1.413, 2.38838, 1, 1, 2.2049, 2.18935, 4.05165, 2.70102, 1.22675},
  ⋯ 16460 ⋯, {UTY, RNA} → {3.16532, 3.28427, 3.06644, 1.77757, 2.90109, 2.48543,
   2.49979, 2.65107, 1, 2.24661, 1.49351, 1.56608, 1.59413, 1.13702, 1}|>
large output | show less | show more | show all | set size limit...
```

Take Log Ratios Compared to Reference in Transcriptome Time Series

Next, we want to use log ratios of expression at any time point compared to a healthy datapoint.

`SeriesApplier[function,data]`	applies a given *function* to *data*, an association of lists, implementing masking for `Missing` values.

Applying a function to a series with Missing data.

We first use `SeriesApplier` to implement the logarithm:

```
In[20]:= timeSeriesRNALog = SeriesApplier[Log, timeSeriesRNA]
Out[20]= <|{LOC729737, RNA} → {1.00795, 0, 1.64009, 1.51152, 1.74367, 0, 0.345715, 0.870615, 0, 0,
   0.790682, 0.783605, 1.39912, 0.993629, 0.204368}, ⋯ 16460 ⋯, {UTY, RNA} → {⋯ 1 ⋯}|>
large output | show less | show more | show all | set size limit...
```

Now we need to compare to use log ratios of expression at any time point compared to a healthy datapoint. We can use the function `SeriesInternalCompare`:

`SeriesInternalCompare[associationOfLists]`	compares each value in each list of *associationOfLists* to an internal reference value in the list, if the reference point itself is not Missing.

Comparing values in a series to an internal reference point in the series.

option name	default value	
CompareFunction	(If[MatchQ[Head[#2], Missing], Missing[], (#1- #2)]&)	The function is used by a Query operation on non-missing input data. Namely: Query[All, CompareFunction[#,#[[ComparisonIndex]]]&]@
ComparisonIndex	1	List position of list value that will be used as a reference data point.
DeleteRule	{Head, Missing}	DeleteRule allows the customization of how to select values for the reference data point for which its key should be deleted. The DeleteRule value takes the structure deleteRuleOptionValue = {MatchQ first argument, MatchQ second argument}. The MatchQ function referred to here is implemented by SeriesInternalCompare internally, and uses the deleteRuleOptionValue as: MatchQ[deleteRuleOptionValue[[1]][reference comparison value], deleteRuleOptionValue[[2]]] The default removes the corresponding key if the value used for reference in the comparison is actually Missing, i.e. the comparison reference point has Head that matches Missing.

Options for SeriesInternalCompare.

We compare every value in each series to the healthy "255" time point, which is the second element in each series:

```
In[21]:= rnaCompared = SeriesInternalCompare[timeSeriesRNALog, ComparisonIndex → 2]

Out[21]= <|{LOC729737, RNA} → {1.00795, 0, 1.64009, 1.51152, 1.74367, 0, 0.345715, 0.870615, 0, 0,
    0.790682, 0.783605, 1.39912, 0.993629, 0.204368}, ··· 16378 ···, {UTY, RNA} → {··· 1 ···}|>
large output   show less   show more   show all   set size limit...
```

Take the Norm and Remove Constant Transcriptome Time Series

Next, we normalize each series, using again SeriesApplier:

```
In[22]:= normedRNACompared = SeriesApplier[Normalize, rnaCompared]

Out[22]= <|{LOC729737, RNA} → {0.268104, 0., 0.436246, 0.402048, 0.463797, 0., 0.0919564,
    0.231574, 0., 0., 0.210313, 0.20843, 0.372152, 0.264294, 0.0543597}, ··· 16378 ···, ··· 1 ···|>
large output   show less   show more   show all   set size limit...
```

ConstantSeriesClean[*dataIn*]	removes constant list series from an association of lists.

Removing constant series.

Finally, we use ConstantSeriesClean to remove constant series, as we are interested in changing time patterns:

```
In[23]:= rnaFinalTimeSeries = ConstantSeriesClean[normedRNACompared]
```

```
Removed series and returning filtered
  list. If you would like a list of removed keys run the
  command ConstantSeriesClean[data,ReturnDropped → True].
```

```
Out[23]= <|{LOC729737, RNA} → {0.268104, 0., 0.436246, 0.402048, 0.463797, 0., 0.0919564,
   0.231574, 0., 0., 0.210313, 0.20843, 0.372152, 0.264294, 0.0543597}, ⋯11628⋯, ⋯1⋯|>
large output | show less | show more | show all | set size limit...
```

Resampling Transcriptome Data

In addition to the above, we want to create a resampled distribution for the transcriptome dataset prior to classification and clustering. In this subsection we first resample the imported and labeled transcriptome dataset, Then, we carry out the full analysis in this "bootstrap" dataset, to create a set of random time series. This bootstrap distribution of time series will be used to provide the cutoffs used in the time series classification in the following subsection.

Resampling the Transcriptome Data

First, we use BootstrapGeneral:

BootstrapGeneral[*omicsObject*, *numberResampled*]	performs a resampling of the *omicsObject* data with replacement, and generates a new association structure with numbering corresponding to the *numberResampled* of new identities.

We can perform resampling of an OmicsObject to create a bootstrap dataset to be used for statistical considerations.

We create a resampling of 100000 sets:

```
In[24]:= rnaBootstrap = BootstrapGeneral[rnaLongitudinal, 100 000]
```

```
Out[24]= <|186 → <|1 → {{6.26661}, {OK}}, 2 → {{13.292}, {OK}}, 3 → {{11.8179}, {OK}},
   4 → {{0.662128}, {OK}}, 5 → {{0}, {OK}}, ⋯99991⋯, 99997 → {{8.89907}, {OK}},
   99998 → {{0.033111}, {OK}}, 99999 → {{0}, {OK}}, 100000 → {{0.0640671}, {OK}}|>,
  255 → <|⋯1⋯|>, ⋯11⋯, 380 → ⋯1⋯, 400 → <|⋯1⋯|>|>
large output | show less | show more | show all | set size limit...
```

Processing the Bootstrap Transcriptome and Creating Bootstrap Time Series

We normalize the transcriptome bootstrap data using the QuantileNormalization function:

```
In[25]:= rnaBootstrapQuantileNormed = QuantileNormalization[rnaBootstrap];
```

We use LowValueTag to tag zero values as Missing[]:

```
In[26]:= rnaBootstrapZeroTagged = LowValueTag[rnaBootstrapQuantileNormed, 0];
```

We next use LowValueTag again to set all FPKM values <1 to unity:

```
In[27]:= rnaBootstrapNoiseAdjusted = LowValueTag[rnaBootstrapZeroTagged, 1, ValueReplacement → 1];
```

Next, we filter out data where the reference point "255" is missing and retain data with at least 3/4 poings available:

In[28]:=
```
rnaBootstrapFiltered = FilterMissing[rnaBootstrapNoiseAdjusted, 3/4, Reference → "255"]
```

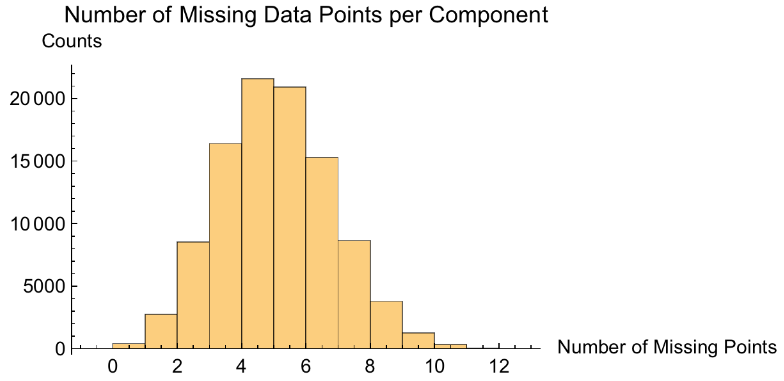

{Missing -> Counts: , <|0 → 410, 1 → 2739, 2 → 8543, 3 → 16 392, 4 → 21 572, 5 → 20 924, 6 → 15 296, 7 → 8652, 8 → 3794, 9 → 1277, 10 → 337, 11 → 56, 12 → 8|>}

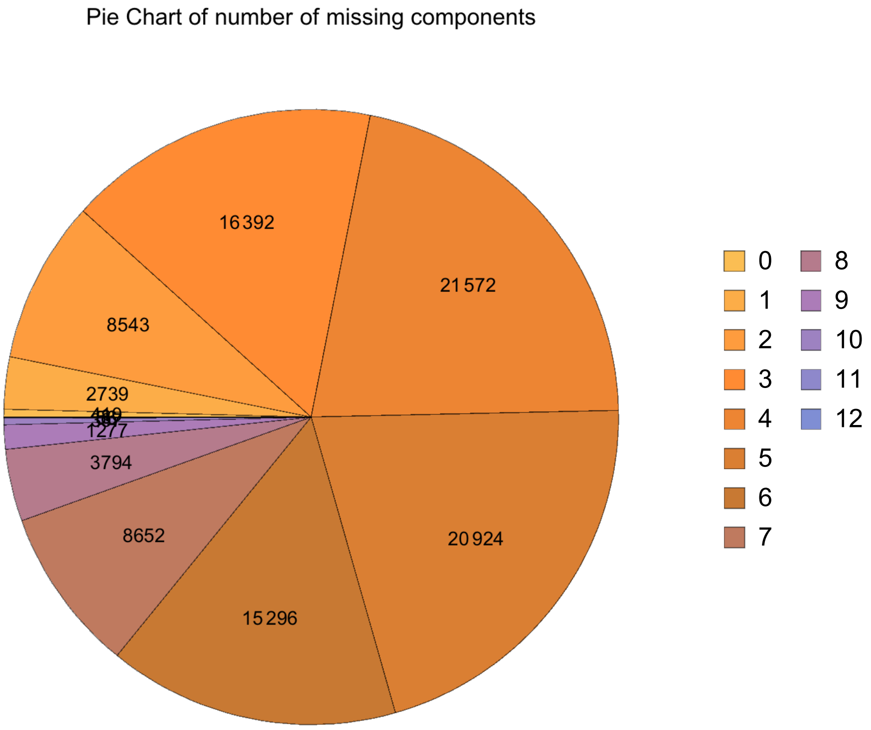

Out[28]=
```
<|186 → <|1 → {{6.26661}, {OK}}, 2 → {{13.292}, {OK}}, 3 → {{11.8179}, {OK}}, 7 → {{1}, {OK}},
   9 → {{12.33}, {OK}}, … 28 074 …, 99 990 → {{Missing[]}, {OK}}, 99 994 → {{38.785}, {OK}},
   99 997 → {{8.89907}, {OK}}, 99 998 → {{1}, {OK}}, 100 000 → {{1}, {OK}}|>, … 13 …, 400 → <| … 1 … |>|>
```

large output | show less | show more | show all | set size limit...

For each bootstrap member we now extract a time series (list of values) corresponding to the series times:

In[29]:=
```
timeSeriesBootstrapRNA = CreateTimeSeries[rnaBootstrapFiltered]
```

Out[29]=
```
<|1 → {6.26661, 57.0833, 22.2908, 1, Missing[], 1, 1,
   41.6445, Missing[], 6.14077, 1, 1, Missing[], 3.33812, 11.3984}, ⋯ 28082 ⋯,
 100000 → {1, 1, 7.03099, 1, Missing[], 1, Missing[], 1, 34.5339, 23.7363, 13.2995, 1, 1, 1, 1}|>
```

large output | show less | show more | show all | set size limit...

We use `SeriesApplier` to implement a logarithm:

In[30]:=
```
timeSeriesBootstrapRNALog = SeriesApplier[Log, timeSeriesBootstrapRNA]
```

Out[30]=
```
<|1 → {1.83524, 4.04451, 3.10417, 0, Missing[],
   0, 0, 3.72917, Missing[], 1.81495, 0, 0, Missing[], 1.20541, 2.43347},
 ⋯ 28082 ⋯, 100000 → {0, 0, 1.95033, 0, Missing[], ⋯ 5 ⋯, 2.58773, 0, 0, 0, 0}|>
```

large output | show less | show more | show all | set size limit...

We compare every value in each series to the healthy "255" time point, which is the second element in each series:

In[31]:=
```
rnaBootstrapCompared = SeriesInternalCompare[timeSeriesBootstrapRNALog, ComparisonIndex → 2]
```

Out[31]=
```
<|1 → {-2.20928, 0., -0.940338, -4.04451, Missing[], -4.04451, -4.04451, -0.315342, Missing[],
   -2.22956, -4.04451, -4.04451, Missing[], -2.8391, -1.61104}, ⋯ 23607 ⋯, 100000 → ⋯ 1 ⋯|>
```

large output | show less | show more | show all | set size limit...

Next, we normalize each series, using again `SeriesApplier`:

In[32]:=
```
normedBootstrapRNACompared = SeriesApplier[Normalize, rnaBootstrapCompared]
```

Out[32]=
```
<|1 → {-0.217389, 0., -0.0925277, -0.397973, Missing[], -0.397973, -0.397973, -0.0310292, Missing[],
   -0.219385, -0.397973, -0.397973, Missing[], -0.279363, -0.158524}, ⋯ 23607 ⋯, 100000 →
  {0., 0., 0.339121, 0., Missing[], 0., Missing[], 0., 0.61587, 0.550676, 0.449952, 0., 0., 0., 0.}|>
```

large output | show less | show more | show all | set size limit...

Finally, we use `ConstantSeriesClean` to remove constant series, as we are interested in changing time patterns:

In[33]:=
```
rnaBootstrapFinalTimeSeries = ConstantSeriesClean[normedBootstrapRNACompared]
```

Out[33]=
```
<|1 → {-0.217389, 0., -0.0925277, -0.397973, Missing[], -0.397973, -0.397973, -0.0310292, Missing[],
   -0.219385, -0.397973, -0.397973, Missing[], -0.279363, -0.158524}, ⋯ 23607 ⋯, 100000 →
  {0., 0., 0.339121, 0., Missing[], 0., Missing[], 0., 0.61587, 0.550676, 0.449952, 0., 0., 0., 0.}|>
```

large output | show less | show more | show all | set size limit...

Classification of Transcriptome Time Series

In this subsection we will classify the transcriptome time series based on patterns in the series. For the classification we will use `TimeSeriesClassification`.

TimeSeriesClassification[*data*, *setTimes*]	takes a *data* association (or list of lists) of values corresponding to intensities collected over time and classifies the values into classes (groups) that show distinct similar temporal patterns. TimeSeriesClassification takes as inputs:<br>*data* — Association with series as values, or a list of series, where the series contain information regarding time intensities/observations. Each series may include Missing data points and may be entered as list of N signal intensities corresponding one–to–one to the N *setTimes* with Missing inserted appropriately if the data is absent, $\{X_1 = X(t_1), X_2 = X(t_2), ..., X_N = X(t_N)\}$. Alternatively, each series data may be a list of pairs of values $\{\{t_1, X_1\}, \{t_2, X_2\}, ..., \{t_N, X_N\}\}$ for only existing measurements.<br>*setTimes* — A global complete set of all possible N times during which all data series could have been collected in the window of the experiment, including times for which no values were reported or are missing, $\{t_1, t_2, ..., t_N\}$.

Classifying a set of time series based on temporal behavior.

option name	default value	
AutocorrelationCutoffs	{0}	Cutoffs, for "Autocorrelation" and "InterpolatedAutocorrelation" methods, for different lags that will be used to filter out data series for which the lags are not within cutoffs. The list length corresponds to cuttofs at different lags, with the ith lag cutoff provided as the ith index, i.e. $\rho_c = \{\rho_{c1}, \rho_{c2}, ..., \rho_{ci}, ..., \rho_{jk}\}$ up to k, where $1 \leq k \leq n$, and typically n = Floor[Length[setTimes] / 2]. The classification will only consider lags up to the length of the list provided. The cutoffs are user–provided and typically calculated through simulation.
AutocorrelationLogic	False	Option to return the autocorrelation logic list for each signal, with the default set to False. If set to True, a logic vector is returned indicating whether or not at a particular lag the autocorrelation for a signal is above or below the AutocorrelationCutoffs.
AutocorrelationOptions	{UpperFrequencyFactor → 1}	Options that are used by the internal Autocorrelation function in the case that the Method → "Autocorrelation" is set.
InterpolationDeltaT	"Auto"	Time step used to grid the time window over which calculations will be performed. If set to "Auto" the step will correspond to dividing the span of the interval into a number of equal steps equal to the number of input time points.

InterpolationOptions	{}	Options list for the internal Interpolation function used to interpolate between data points that have Missing values or uneven spacing.
LombScargleCutoff	0	Cutoff value for "LombScargle" method, for filtering the highest intensity observed in the power spec– trum. The cutoff is user–provided and typically calculated through simulation.
LombScargleOptions	{PairReturn→ False, NormalizeIntensi- ties→ True}	Options that are used by the internal LombScargle function if the case that the Method → "LombScargle" is set.
Method	"LombScargle"	Selection of which algorithm to use in the classifica· tion scheme.
ReturnAllSpikes	False	Option whether each signal may maintain unique membership to each spike class, or be allowed to belong to multiple classes. Used in "Autocorrelation" and "InterpolatedAutocorrelation" methods. If set to False, first spike maxima are classified, and only signals found not to belong to spike maxima are then considered for membership in the spike minima class.
ReturnData	True	If set to True will return input keys to data associa– tions in the classification. If set to False will only return the keys of the input data in the classification.
ReturnModels	False	Whether to return the models as well as the classifi– cation information for the input data. The data is returned as an association with the key "TimeSeriesClasses" for classification groups and one of the following: (i) "Models" for model–based methods, (ii) "LombScargle" for periodograms in the "LombScargle" method, (iii) "Autocorrelations" for autocorrelation based methods.
SpikeCutoffs	<|1 →{.99,-99}, 2 → {.99,-99}|>	Association with number, n, of data points as keys, and values corresponding to cutoffs, in the form <|n → {Maximum Spike Cutoff$_n$, Minimum Spike Cutoff$_n$}|> used to call spike maxima and minima for a time series with this number of datapoints. The values are provided by the user depending on data approach based on simulation. The default values are only place– holders and should be replaced by real values. The association must have corresponding keys for all lengths of input datasets, so that Keys[OptionValue[SpikeCutoffs]] ∈ {Possible lengths of numeric data}. , i.e. all possible lengths of series constructed by excluding Missing or other non–numeric values).

Options for TimeSeriesClassification .

TimeSeriesClassification uses multiple methods to classify data. The periodogram/autocorrelation methods used use cutoffs from simulation/user-provided values, to assess class membership based on statistical significance. In this tutorial we will use the "LombScargle" method, to classify data based on a Lomb-Scargle computation of a periodogram. The data is classified based into classes major (highest intensity) frequencies based on the generated periodogram for a signal, when the intensity of this frequency is above an intensity threshold cutoff. Additionally, data that displays spikey behavior

in the real intensity, that is not classified into any frequency classes, is classified as a SpikeMaximum or SpikeMinimum if the spike is higer or lower respectively than what one would expect from a random signal.

Method	Description
"LombScargle"	Classification based on periodograms (power spectra) generated by a Lomb–Scargle computation as implemented internally by the `LombScargle` function. The data is classified into classes of major (highest intensity) frequencies and spikes (maxima or minima in real signal intensity), depending on cutoffs typically provided by simulation and passed to the function by the `LombScargleCutoffs` and `SpikeCutoffs` option values. The returned {computed classification vector} for this method is the intensity list of the periodogram for each signal.
"Autocorrelation"	Classification based on autocorrelations generated by a Lomb–Scargle approach using an inverser Fourier transform of spectral intensities, as implemented through the `Autocorrelation` function. The data is classified into autocorrelations at different lags and spikes (maxima or minima) classes, depending on cutoffs typically provided by simulation. The returned {computed classification vector} for this method is the autocorrelation list for each signal.
"InterpolatedAutocorrelation"	Classification based on autocorrelations generated directly in time, with `Missing` data handled through interpolation. The data is classified into autocorrelations at different lags and spikes (maxima or minima) classes depending on cutoffs typically provided by simulation. The returned {computed classification vector} for this method is the autocorrelation list for each signal.
"TimeSeriesModelAggregate"	Classification based on model fitting of time series through `TimeSeriesModelFit` and all available models therein. The data is classified into aggregate model classes. The returned {computed classification vector} for this method is the actual input signal.
"TimeSeriesModelDetailed"	Classification based on model fitting of time series through `TimeSeriesModelFit` and all available models therein. The data is classified into model classes based on individual model degree parameters. The returned {computed classification vector} for this method is the "BestFitParameters" for the model fit. If this list is empty an integer list is returned {token integer} – this is used in subsequent clustering applications.

Methods for `TimeSeriesClassification`.

To create the cutoffs for the classification we will first use the bootstrap time series set created in the previous subsection, and `QuantileEstimator`.

`QuantileEstimator`[*data*, *timepoints*]	obtains the quantile estimator following bootstrap for time series. It takes as inputs:<br>*data* — Association or list with series as values, from which to generate a distribution.<br>*timepoints* — Timepoints over which the time series run.

Estimating the quantile value that can be used as a cutoff for classification of time series based on bootstrap simulations.

option name	default value	
AutocorrelationOptions	{}	Specific options when calculating autocorrelations for the time series.
InterpolationDeltaT	"Auto"	Time step used to grid the time window over which calculations will be performed. If set to "Auto" the step will correspond to dividing the span of the interval into a number of equal steps equal to the number of input time points.
InterpolationOptions	{}	Options list for the internal Interpolation function used to interpolate between data points that have Missing values or uneven spacing.
LombScargleOptions	{PairReturn → False, NormalizeIntensities→ True}	Specific options when calculating LombScargle periodograms for the time series.
Method	"LombScargle"	Method of calculation. Choices include one of the following: {"LombScargle","Autocorrelation", "InterpolatedAutocorrelation","Spikes"}
QuantileValue	0.95	Which quantile to extract.

Options for QuantileEstimator .

Depending on the cutoffs we would like to generate, we select the appropriate Method (also considering the Method that the downstream TimeSeriesClassification will use).

Method	Description
"Autocorrelation"	List of values corresponding to selected quantile of autocorrelations, with the ith lag quantile provided as the ith index, i.e. ρ_c={ρ_{c1},ρ_{c2},,...,ρ_{ci},,..., ρ_{ck}} up to k lags, where 1≤ k ≤ n, and typically n=Floor[Length[timepoints]/2]. The method utilizes the Autocorrelation function internally.
"InterpolatedAutocorrelation"	List of values corresponding to selected quantile for autocorrelations, with the ith lag quantile provided as the ith index, i.e. ρ_c={ρ_{c1},ρ_{c2},,...,ρ_{ci},,..., ρ_{ck}} up to k lags, where 1≤ k ≤ n, and typically n=(Length[timepoints]−1). The method utilizes an Interpolation followed by a CorrelationFunction implementation to compute autocorrelations, i.e. missing data or uneven sampling is handled by data interpolation.
"LombScargle"	Single value corresponding to selected quantile of maximum peak intensity of periodogram. The method utilizes the LombScargle function internally.
"Spikes"	Association with number, n, of data points as keys, and values corresponding to quantiles for maxima and minima of the series, in the form <|n → {Maximum Spike Quantile$_n$, Maximum Spike Quantile$_n$}|> . The keys are generated automatically so that so that Keys[output] ∈ {Possible lengths of numeric data}. , i.e. all possible lengths of input series constructed by excluding Missing or other non−numeric values).

Method selection and output for QuantileEstimator .

The default output for TimeSeriesClassification is an Association with outer keys being the classification classes, inner keys being the class members, and each class member value being a list of

{{computed classification vector}, {input data list}}. The general output structure is for M output classes of each having m_i members:

```
<| Class_1 → <|Member_11 → {{classification vector_11}, {input data vector_11}},
   Member_12 → {{classification vector_12}, {input data vector_12}}, ...,
   Member_1m_1 → {{classification vector_1m_1}, {input data vector_1m_1}}|>,
 Class_2 → <|Member_21 -> {{classification vector_21}, {input data vector_21}},
   Member_22 -> {{classification vector_22}, {input data vector_22}}, ...,
   Member_2m_2 → {{classification vector_2m_2}, {input data vector_2m_2}}|>, ...,
 Class_M → <|Member_M1 -> {{classification vector_M1}, {input data vector_M1}},
   Member_M2 -> {{classification vector_M2}, {input data vector_M2}}, ...,
   Member_Mm_M → {{classification vector_Mm_M}, {input data vector_Mm_M}}|>|>
```

Before we classify our transcriptome data, we estimate for the "LombScargle" Method a 0.95 quantile cutoff from the bootstrap transcriptome data:

```
In[34]:= q95RNA = QuantileEstimator[rnaBootstrapFinalTimeSeries, timesRNA]
Out[34]= 0.859043
```

Next, we estimate the "Spikes" 0.95 quantile cutoff from the bootstrap transcriptome data:

```
In[35]:= q95RNASpikes = QuantileEstimator[rnaBootstrapFinalTimeSeries, timesRNA, Method → "Spikes"]
Out[35]= <|12 → {0.821455, -0.445414}, 13 → {0.803486, -0.427123},
  14 → {0.780647, -0.401738}, 15 → {0.755499, -0.379251}|>
```

Now we can classify the transcriptome time series data based on these cutoffs:

```
In[36]:= rnaClassification = TimeSeriesClassification[rnaFinalTimeSeries,
   timesRNA, LombScargleCutoff → q95RNA, SpikeCutoffs → q95RNASpikes]
   Method → "LombScargle"
Out[36]= <| ··· 1 ··· |>
  large output | show less | show more | show all | set size limit...
```

The default output for TimeSeriesClassification is an Association with outer keys being the classification classes, inner keys being the class members, and each class member value being a list of {{computed classification vector}, {input data list}}. The general output structure is for M output classes of each having m_i members:


```
<| Class_1 → <|Member_11 → {{classification vector_11}, {input data vector_11}},
   Member_12 → {{classification vector_12}, {input data vector_12}}, ...,
   Member_1m_1 → {{classification vector_1m_1}, {input data vector_1m_1}}|>,
 Class_2 → <|Member_21 -> {{classification vector_21}, {input data vector_21}},
   Member_22 -> {{classification vector_22}, {input data vector_22}}, ...,
   Member_2m_2 → {{classification vector_2m_2}, {input data vector_2m_2}}|>, ...,
 Class_M → <|Member_M1 -> {{classification vector_M1}, {input data vector_M1}},
   Member_M2 -> {{classification vector_M2}, {input data vector_M2}}, ...,
   Member_Mm_M → {{classification vector_Mm_M}, {input data vector_Mm_M}}|>|>
```


If we want the classes produced, we can query the keys:

```
In[37]:= Keys[rnaClassification]
Out[37]= {SpikeMax, SpikeMin, f1, f2, f3, f4, f5, f6, f7}
```

For the number of members in each class we have:

In[38]:=
```
Query[All, Length]@rnaClassification
```

Out[38]=
```
<|SpikeMax → 600, SpikeMin → 8507, f1 → 58, f2 → 3, f3 → 13, f4 → 40, f5 → 14, f6 → 10, f7 → 56|>
```

We can obtain the membership list in any class of interest:

In[39]:=
```
Query["f1", Keys]@rnaClassification
```

Out[39]=
```
{{HPCAL4, RNA}, {C1orf35, RNA}, {EPC1, RNA}, {CCSER2, RNA}, {ARL3, RNA}, {ADD3, RNA}, {PDZD8, RNA},
 {PVRL1, RNA}, {SORL1, RNA}, {XPOT, RNA}, {UHRF1BP1L, RNA}, {VWA8, RNA}, {KIAA0586, RNA},
 {SNURF, RNA}, {NEO1, RNA}, {CMIP, RNA}, {CYB5D1, RNA}, {MED1, RNA}, {TSHZ1, RNA}, {ZNF507, RNA},
 {CENPBD1P1, RNA}, {NCOA1, RNA}, {PNPT1, RNA}, {MARCH7, RNA}, {PLCL1, RNA}, {PRNP, RNA},
 {XRN2, RNA}, {LOC284801, RNA}, {RPN2, RNA}, {NRIP1, RNA}, {NPTXR, RNA}, {PPARA, RNA},
 {COMMD2, RNA}, {KPNA4, RNA}, {LRCH3, RNA}, {SEPT11, RNA}, {SEC31A, RNA}, {GUCY1A3, RNA},
 {FNIP2, RNA}, {KIAA1430, RNA}, {ZFR, RNA}, {JMY, RNA}, {SEC24A, RNA}, {PTP4A1, RNA}, {HACE1, RNA},
 {ZBTB24, RNA}, {HECA, RNA}, {RP9, RNA}, {BBS9, RNA}, {ATXN7L1, RNA}, {C7orf60, RNA}, {MFHAS1, RNA},
 {ADAM9, RNA}, {KAT6A, RNA}, {PTENP1, RNA}, {CDK20, RNA}, {RPS6KA3, RNA}, {CASK, RNA}}
```

We may also want to know what these frequencies correspond to. The "LombScargle" method uses a `LombScargle` transformation.

`LombScargle[`*data*, *setTimes*`]`	calculates the Lomb–Scargle power spectrum for time series *data* that runs over specified *setTimes*. It takes as input:

data	Time series (data as a list; list may be the value of a single key in an association). The series may include Missing data points. Data may be entered as list of N signal intensities corresponding one–to–one to the N setTimes with Missing inserted appropriately if the data is absent, $\{X_1=X(t_1), X_2=X(t_2), ..., X_N=X(t_N)\}$. Alternatively, the data may be a list of pairs of values $\{\{t_1,X_1\},\{t_2,X_2\},...,\{t_N,X_N\}\}$ for only existing measurements.
setTimes	A complete set of all possible N times during which data could have been collected in the window of the experiment, including times for which no data was collected, $\{t_1, t_2, ..., t_N\}$.

Calculating the power spectrum of a (possibly unevenly sampled) time series.

option name	default value	
FrequenciesOnly	False	Whether to return only the computation frequencies. An association of frequencies "f" ordered from low to high by index i is returned in the form: <|"f1" → $frequency_1$, "f2" → $frequency_2$, ..., "fi" → $frequency_{i,...,}$ "fn" → $frequency_n$ |>
NormalizeIntensities	False	Whether the intensities list should be normalized or not.
OversamplingRate	1	Rate at which to oversample the time series using zero-padding.
PairReturn	False	Whether data should be returned as {frequency list,intensity list} or as pairs: {{frequency1,intensity1}, {frequency2, intensi-ty2},...,{frequencyN,intensityN}.
UpperFrequencyFactor	1	Value ≥ 1, by which to scale the upper Nyquist cutoff frequency and increase spectral resolution.

Options for LombScargle.

To obtain the possible frequencies we simply run LombScargle over the desired times for one of the time series and set the FrequenciesOnly option to True:

```
In[40]:= LombScargle[rnaFinalTimeSeries[[1]], timesRNA, FrequenciesOnly → True]

Out[40]= <|f1 → 0.00500668, f2 → 0.0104306, f3 → 0.0158545,
          f4 → 0.0212784, f5 → 0.0267023, f6 → 0.0321262, f7 → 0.0375501|>
```

Proteomic Data

Importing OmicsObject Proteome Data

We now import the proteomics data example (for details on how to import such data please refer to DataImporter, DataImporterDirect, DataImporterDirectLabeled and OmicsObjectCreator documentation).

We import the proteomics OmicsObject MathIOmica example:

```
In[41]:= proteinExample = Get[FileNameJoin[{ConstantMathIOmicaExamplesDirectory, "proteinExample"}]]

Out[41]= <|7 → <|{A0AVT1, Protein} → {{0.937}, {17}}, {A0FGR8, Protein} → {{1.073}, {24}},
            {A0MZ66, Protein} → {{1.059}, {9}}, ⋯5219⋯, {Q9Y6I4, Protein} → Missing[],
            {Q9Y6I9, Protein} → Missing[], {Q9Y6X3, Protein} → Missing[]|>,
          9 → <|⋯1⋯|>, ⋯9⋯, 20 → ⋯1⋯, 21 → <|⋯1⋯|>|>

         large output | show less | show more | show all | set size limit...
```

There are multiple samples given by the outer associations. We can use Query to get any data. For example we can get the outer keys:

```
In[42]:= Query[Keys]@proteinExample

Out[42]= {7, 9, 10, 11, 14, 12, 13, 15, 16, 17, 19, 20, 21}
```

We notice that sample 8 is missing - this is because it was used as a reference in the proteomics experiment. Point 18 is missing as there was no sample for that time point. We will address this in the next section.

We can get the expression raw data from any sample and entry. For example, the 14th and 214th entries in sample 12:

In[43]:=
```
Query["12", {14, 22}]@proteinExample
```

Out[43]=
```
<|{A5PLN9, Protein} → {{1.057}, {3}}, {A6NGU5, Protein} → Missing[]|>
```

The keys correspond to UniProt accessions, and have been tagged with a "Protein" label as well. The values of all the keys/IDs correspond to `{{measurements}, {metadata}}`, and in this particular example: `{{relative intensity compared to reference}, {number of unique peptides identified for the given protein}}`.

The measurement for each protein is a relative intensity, i.e. the ratio of the value for the protein compared to the reference timepoint that has been chosen as the healthy sample "8", day "255" (in the experiment this was TMT reporter with 126 amu). The last list, the "metadata", in the proteomics OmicsObject was chosen to be the number of unique peptides identified for the given protein.

Additional Information: Gene Translation

As an aside, let us consider the form of the protein identifiers. MathIOmica can perform basic `GeneTranslation` going from one kind of identifier to another, using `GetGeneDictionary` :

`GeneTranslation` [*inputIDList*, *targetIDList*, *geneDictionary*]	uses *geneDictionary* to convert *inputIDList* IDs to different annotations as indicated by *targetIDList*. It takes for inputs:<br>`inputIDList` — List of n IDs (strings) to be converted in the form {inputID$_1$, inputID$_2$, ..., inputID$_n$}<br>`targetIDList` — List of target identifier strings, as used in the gene geneDictionary, {target ID$_1$, targetID$_2$, ... target ID$_k$} e.g. {"UniProt ID","Gene Symbol"}. Can also be provided as a single string for only one kind of IDs.<br>`geneDictionary` — Gene dictionary to base translation on in the form generated by `GetGeneDictionary` .
`GetGeneDictionary` []	creates an ID/accession dictionary from a UCSC table search – typically of gene annotations. GetGeneDictionary uses MathIOmica data for the annotations..

Translating gene identifiers using a gene dictionary.

We use GetGeneDictionary to define a gene dictionary:

In[44]:=
```
geneDictionary = GetGeneDictionary[]
```

Out[44]=
```
<|human → <|UCSC ID → {uc001aaa.3, uc010nxr.1, uc010nxq.1, uc001aal.1, uc001aaq.2, uc001aar.2,
    uc001aau.3, uc021oeh.1, ⋯ 121 567 ⋯, uc022cfk.1, uc031tkn.1, uc022cgh.1, uc022cha.1,
    uc022chb.1, uc022chc.1, uc022che.1, uc022cpe.1}, ⋯ 6 ⋯, HGU ... x ID → ⋯ 1 ⋯|>|>
```

large output | show less | show more | show all | set size limit...

The current version of the gene dictionary has accessions for the following identifiers:

In[45]:=
```
Query[All, Keys]@geneDictionary
```

Out[45]=
```
⟨|human → {UCSC ID, UniProt ID, Gene Symbol, RefSeq ID,
   NCBI Protein Accession, Ensembl ID, KEGG Gene ID, HGU133Plus2 Affymetrix ID}|⟩
```

We can now use GeneTranslation (setting the optional InputID to "UniProt ID") to convert our example "UniProt ID" accessions to "Gene Symbol":

In[46]:=
```
GeneTranslation[{"A5PLN9", "A6NGU5"}, {"Gene Symbol"}, geneDictionary, InputID → {"UniProt ID"}]
```

Out[46]=
```
⟨|Gene Symbol → ⟨|A5PLN9 → {TRAPPC13}, A6NGU5 → Missing[]|⟩|⟩
```

We note that an ID might not necessarily be annotated across all databases, as in the above example.

Processing of Proteome Data

We will next preprocess the imported proteome data. We will first perform a transformation on the data towards a normal distribution, then we will re-label the samples with real time and carry out filtering for unique peptides present in each protein identification, as well as for missing data. Finally, we will create the proteomics time series or relative intensities compared to the healthy reference point for each protein.

Power Transformation, Labeling and Filtering

Data Power Transformation

To make the data comparable across time points, and as close to a normal distribution as possible for each sample, we normalize each time point /sample by using `ApplyBoxCoxTransform`.

`ApplyBoxCoxTransform[`*data*`]`	for a given *data* set, computes the Box–Cox transformation at the maximum likelihood λ parameter.

Applying a power transformation (Box-Cox) for an optimized parameter for each dataset.

option name	default value	
`ListIndex`	`Missing[]`	Selection of which list to use in the OmicsObject input.
`ComponentIndex`	`Missing[]`	Selection of which component of a list to use in the OmicsObject input.
`HorizontalSelection`	`False`	Horizontal selection across components for a single level association with multi–list values.

Options for `ApplyBoxCoxTransform`.

We apply a Box-Cox transformation to the proteomics data measurement in the OmicsObject, which is in the first list first component for each identifier. The optimized $\hat{\lambda}$ parameter for each sample is printed out for reference:

In[47]:=
```
transformedProteinData = ApplyBoxCoxTransform[proteinExample, ListIndex → 1, ComponentIndex → 1]
```

```
Calculated Box-Cox parameter λ̂ = -0.152638
Calculated Box-Cox parameter λ̂ = -0.177086
Calculated Box-Cox parameter λ̂ = -0.421581
Calculated Box-Cox parameter λ̂ = -0.292287
Calculated Box-Cox parameter λ̂ = -0.432042
Calculated Box-Cox parameter λ̂ = 0.346673
Calculated Box-Cox parameter λ̂ = 0.368061
Calculated Box-Cox parameter λ̂ = 0.0834073
Calculated Box-Cox parameter λ̂ = 0.13413
Calculated Box-Cox parameter λ̂ = 0.166336
Calculated Box-Cox parameter λ̂ = 0.0866284
Calculated Box-Cox parameter λ̂ = -0.199247
Calculated Box-Cox parameter λ̂ = -0.221778
```

Out[47]=

```
<|7 → <|{A0AVT1, Protein} → {{-0.0653962}, {17.}}, {A0FGR8, Protein} → {{0.0700809}, {24.}},
   … 5221 …, {Q9Y6I9, Protein} → Missing[], {Q9Y6X3, Protein} → Missing[]|>,
   … 11 …, 21 → <|{A0AVT1, Protein} → {{- … 21 …}, … 1 …}, … 5223 …, … 1 …|>|>
```

large output | show less | show more | show all | set size limit...

We can plot the data to see what the resulting distributions look like:

In[48]:=

```
Histogram[#] & /@ (Query[All, Values, 1, 1]@transformedProteinData)
```

Out[48]=

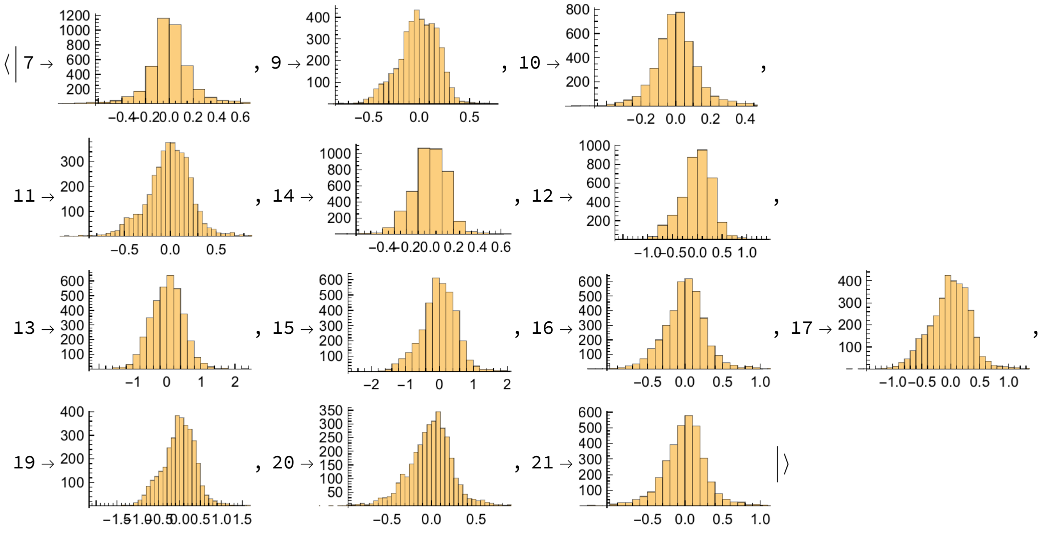

Re-labeling Samples with Times

As with the transcriptome, we notice that the sample numberings do not correspond to actual days, so we may adjust using the sampleToDays association created before and reproduced here for reference:

```
In[49]:= sampleToDays =
  <|"7" → "186", "8" → "255", "9" → "289", "10" → "290", "11" → "292", "12" → "294", "13" → "297", "14" → "301",
   "15" → "307", "16" → "311", "17" → "322", "18" → "329", "19" → "369", "20" → "380", "21" → "400"|>;
```

We can now do a KeyMap to rename the outer keys:

```
In[50]:= proteinLongitudinal = KeyMap[sampleToDays, transformedProteinData]

Out[50]= <|186 → <|{A0AVT1, Protein} → {{-0.0653962}, {17.}},
    {A0FGR8, Protein} → {{0.0700809}, {24.}}, {A0MZ66, Protein} → {{0.057075}, {9.}}, … 5220 …,
    {Q9Y6I9, Protein} → Missing[], {Q9Y6X3, Protein} → Missing[] |>, … 11 …, 400 → <| … 1 … |> |>

large output | show less | show more | show all | set size limit...
```

Now let's check the timepoints in this dataset:

```
In[51]:= timesProteinRawData = TimeExtractor[proteinLongitudinal]

Out[51]= {186, 289, 290, 292, 294, 297, 301, 307, 311, 322, 369, 380, 400}
```

We notice a small complication: there are two timepoints missing, compared to the transcriptome: (i) the reference time point "255" does not appear explicitely in our computation (corresponding to a zero value about which other timepoints are computed for proteins with at least 2 unique peptides). (ii) there is no sample for day "329".

We can use the `ConstantAssociator` function to append these to the transformed data. timepoints "255" (zero measurement assumed to have at least 2 unique peptides available per protein) and "329", assumed to be Missing data:

```
In[52]:= proteinLongitudinalEnlarged =
  ConstantAssociator[proteinLongitudinal, <|"255" → {{0}, {2}}, "329" → Missing[]|>]

Out[52]= <|186 → <|{A0AVT1, Protein} → {{-0.0653962}, {17.}},
    {A0FGR8, Protein} → {{0.0700809}, {24.}}, {A0MZ66, Protein} → {{0.057075}, {9.}}, … 5220 …,
    {Q9Y6I9, Protein} → Missing[], {Q9Y6X3, Protein} → Missing[] |>, … 13 …, 329 → <| … 1 … |> |>

large output | show less | show more | show all | set size limit...
```

We can now check the timepoints again:

```
In[53]:= timesProtein = TimeExtractor[proteinLongitudinalEnlarged]

Out[53]= {186, 255, 289, 290, 292, 294, 297, 301, 307, 311, 322, 329, 369, 380, 400}
```

Filter Unique Peptides

Typically, proteomics data from mass spectrometry is filtered to retain only identifications of proteins that are supported by at least 2 unique peptides having been identified per protein. We can use `FilteringFunction` to implement the filtering:

`FilteringFunction`[*omicsObject*, *cutoff*]	filters OmicsObject data by a chosen comparison (by default greatr or equal) to a *cutoff*.

FilteringFunction can be used to filter data in an OmicsObject.

option name	default value	
ListIndex	Missing[]	Selection of which list to use in the OmicsObject input.
ComponentIndex	Missing[]	Selection of which component of a list to use in the OmicsObject input.
SelectionFunction	GreaterEqual	Selection of comparison to use for filtering.

Options for FilteringFunction .

We filter out proteomics data with less than 2 unique peptides per protein. The unique peptides is reported as the second list, first component in the OmicsObject values in this case:

```
In[54]:= proteinUnique = FilteringFunction[proteinLongitudinalEnlarged, 2, ListIndex → 2, ComponentIndex → 1]
```

```
Out[54]= <|186 → <|{A0AVT1, Protein} → {{-0.0653962}, {17.}},
    {A0FGR8, Protein} → {{0.0700809}, {24.}}, {A0MZ66, Protein} → {{0.057075}, {9.}}, … 5220 …,
    {Q9Y616, Protein} → Missing[], {Q9Y6X3, Protein} → Missing[] |>, … 13 …, 329 → <| … 1 … |> |>
```

large output | show less | show more | show all | set size limit...

Filter Data

We will next remove values that have been tagged as Missing[], retaining data that have at least 3/4 data points available across all samples. Here we use the function FilterMissing:

```
In[55]:= filteredProteinData = FilterMissing[proteinUnique, 3/4]
```

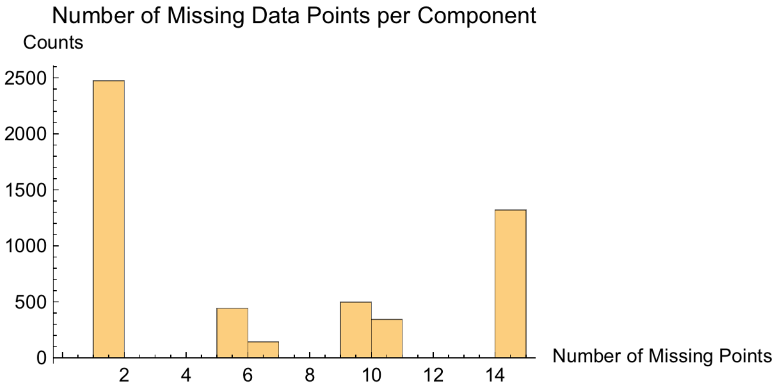

{Missing -> Counts: , <|1 → 2475, 5 → 443, 6 → 143, 9 → 499, 10 → 345, 14 → 1320|>}

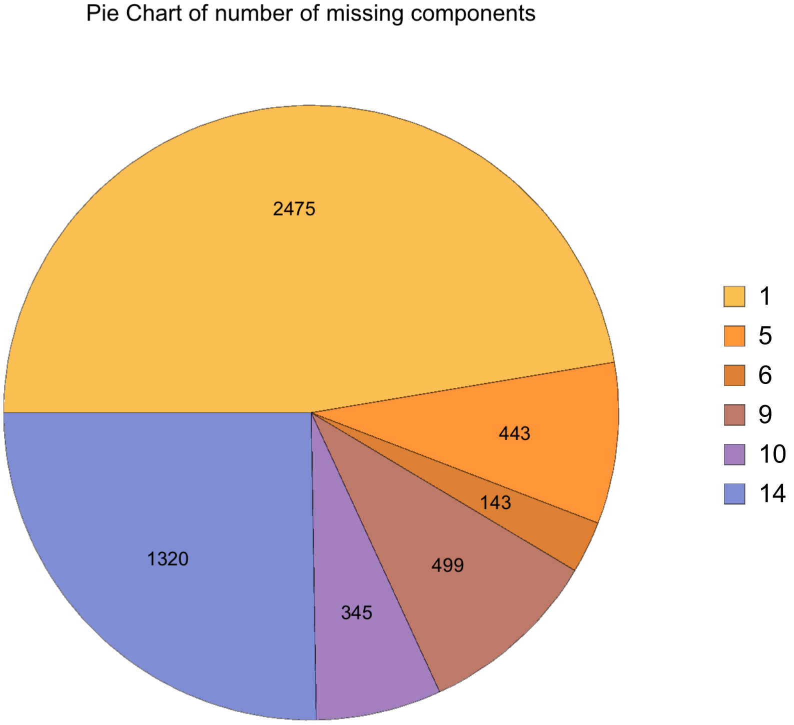

Out[55]=

```
<|186 → <|{A0AVT1, Protein} → {{-0.0653962}, {17.}},
   {A0FGR8, Protein} → {{0.0700809}, {24.}}, ⋯ 2471 ⋯, {Q9Y6W5, Protein} → {{-0.0514946}, {14.}},
   {Q9Y6Y8, Protein} → {{-0.026397}, {10.}}|>, ⋯ 13 ⋯, 329 → <| ⋯ 1 ⋯|>|>
```

large output | show less | show more | show all | set size limit...

Create Proteome Time Series

We can now create time series for each of the proteins.

For each protein we now extract a time series (list of values) corresponding to these times:

```
In[56]:= timeSeriesProtein = CreateTimeSeries[filteredProteinData]

Out[56]= <|{A0AVT1, Protein} → {-0.0653962, 0, 0.00299471, -0.0348449, -0.0182123, 0.0627073, ··· 3 ···,
    0.0829594, 0.0689856, Missing[], -0.050132, -0.137674, -0.0120888}, ··· 2473 ···, ··· 1 ··· |>
large output | show less | show more | show all | set size limit...
```

Take the Norm and Remove Constant Proteome Time Series

Next, we normalize each protein series, using `SeriesApplier` :

```
In[57]:= normedProteinAll = SeriesApplier[Normalize, timeSeriesProtein]

Out[57]= <|{A0AVT1, Protein} → {-0.205122, 0., 0.00939321, -0.109294, -0.0571245, 0.196687, 0.529638,
    0.0740093, -0.539241, 0.26021, 0.21638, Missing[], -0.157244, -0.431828, -0.0379175},
  ··· 2473 ···, {Q9Y6Y8, Protein} → {-0.0502772, 0., 0.0961208, 0.0848518, 0.207372,
    0.188143, ··· 3 ···, 0.134835, -0.133348, Missing[], -0.185135, 0., -0.369519} |>
large output | show less | show more | show all | set size limit...
```

Finally, we use `ConstantSeriesClean` to remove constant series, as we are interested in changing time patterns:

```
In[58]:= proteinFinalTimeSeries = ConstantSeriesClean[normedProteinAll]

Out[58]= <|{A0AVT1, Protein} → {-0.205122, 0., 0.00939321, -0.109294, -0.0571245, 0.196687, 0.529638,
    0.0740093, -0.539241, 0.26021, 0.21638, Missing[], -0.157244, -0.431828, -0.0379175},
  ··· 2473 ···, {Q9Y6Y8, Protein} → {-0.0502772, 0., 0.0961208, 0.0848518, 0.207372,
    0.188143, ··· 3 ···, 0.134835, -0.133348, Missing[], -0.185135, 0., -0.369519} |>
large output | show less | show more | show all | set size limit...
```

Resampling Proteome Data

In addition to the above, we want to create a resampled distribution for the proteome dataset prior to classification and clustering. In this subsection we first resample the imported and labeled proteome dataset, Then, we carry out the full analysis in this "bootstrap" dataset, to create a set of random proteome time series. This bootstrap distribution of time series will be used to provide the cutoffs used in the time series classification in the following subsection.

Resampling the Proteome Data

We create a resampling of 100000 sets:

```
In[59]:= proteinBootstrap = BootstrapGeneral[proteinExample, 100 000]

Out[59]= <|7 → <|1 → {{0.943}, {13}}, 2 → {{1.033}, {4}}, 3 → {{1.048}, {3}}, 4 → Missing[], 5 → Missing[],
    6 → {{0.946}, {31}}, ··· 99 988 ···, 99 995 → Missing[], 99 996 → {{0.948}, {19}}, 99 997 → {{0.993}, {6}},
    99 998 → {{0.876}, {2}}, 99 999 → {{1.102}, {39}}, 100 000 → {{0.906}, {14}} |>, ··· 11 ···, 21 → <| ··· 1 ··· |> |>
large output | show less | show more | show all | set size limit...
```

Processing the Bootstrap Proteome and Creating Bootstrap Time Series

We apply a Box-Cox transformation to the bootstrap set proteomics data measurement in the OmicsObject, which is in the first list first component for each identifier. The optimized $\hat{\lambda}$ parameter for each sample is printed out for reference:

In[60]:=
```
transformedProteinBootstrapData = ApplyBoxCoxTransform[proteinBootstrap, ListIndex → 1, ComponentIndex → 1]
```

```
Calculated Box-Cox parameter λ̂ = -0.210137
Calculated Box-Cox parameter λ̂ = -0.209165
Calculated Box-Cox parameter λ̂ = -0.412662
Calculated Box-Cox parameter λ̂ = -0.28829
Calculated Box-Cox parameter λ̂ = -0.454618
Calculated Box-Cox parameter λ̂ = 0.346507
Calculated Box-Cox parameter λ̂ = 0.385014
Calculated Box-Cox parameter λ̂ = 0.0797884
Calculated Box-Cox parameter λ̂ = 0.165965
Calculated Box-Cox parameter λ̂ = 0.143803
Calculated Box-Cox parameter λ̂ = 0.0989413
Calculated Box-Cox parameter λ̂ = -0.184469
Calculated Box-Cox parameter λ̂ = -0.234572
```

Out[60]=
```
<|7 → <|1 → {{-0.0590524}, {13.}}, 2 → {{0.0323567}, {4.}},
  3 → {{0.0466534}, {3.}}, 4 → Missing[], ··· 99 993 ···, 99 998 → {{-0.134248}, {2.}},
  99 999 → {{0.0961422}, {39.}}, 100 000 → {{-0.099747}, {14.}}|>, ··· 11 ···, 21 → <| ··· 1 ··· |>|>
```

large output | show less | show more | show all | set size limit...

We can now do a KeyMap to rename the outer keys to actual days:

In[61]:=
```
proteinBootstrapLongitudinal = KeyMap[sampleToDays, transformedProteinBootstrapData];
```

Now let's check the timepoints in this dataset:

In[62]:=
```
timesProteinBootstrapData = TimeExtractor[proteinBootstrapLongitudinal]
```

Out[62]=
```
{186, 289, 290, 292, 294, 297, 301, 307, 311, 322, 369, 380, 400}
```

As with the regular protein data above use the `ConstantAssociator` function to append these to the transformed bootstrap data. Timepoints "255" (zero measurement assumed to have at least 2 unique peptides available per protein) and "329", assumed to be Missing data:

In[63]:=
```
proteinBootstrapLongitudinalEnlarged =
  ConstantAssociator[proteinBootstrapLongitudinal, <|"255" → {{0}, {2}}, "329" → Missing[]|>];
```

We can now check the timepoints again:

In[64]:=
```
timesProteinBootstrap = TimeExtractor[proteinBootstrapLongitudinalEnlarged]
```

Out[64]=
```
{186, 255, 289, 290, 292, 294, 297, 301, 307, 311, 322, 329, 369, 380, 400}
```

We filter out proteomics bootstrap data with less than 2 unique peptides per protein. The unique peptides is reported as the second list, first component in the OmicsObject values in this case:

In[65]:=
```
proteinBootstrapUnique =
 FilteringFunction[proteinBootstrapLongitudinalEnlarged, 2, ListIndex → 2, ComponentIndex → 1]
```

Out[65]=
```
<|186 →
  <|1 → {{-0.0590524}, {13.}}, 2 → {{0.0323567}, {4.}}, 3 → {{0.0466534}, {3.}}, 6 → {{-0.0558378}, {31.}},
   ⋯99992⋯, 3321 → Missing[], 28249 → Missing[], 85458 → Missing[], 99991 → Missing[]|>,
 289 → <|⋯1⋯|>, ⋯11⋯, 255 → ⋯1⋯, 329 → <|⋯1⋯|>|>
```

large output | show less | show more | show all | set size limit...

We will next remove values that have been tagged as Missing[], retaining data that have at least 3/4 data points available across all bootstrap samples. Here we use the function `FilterMissing`:

In[66]:=
```
filteredProteinBootstrapData = FilterMissing[proteinBootstrapUnique, 3/4]
```

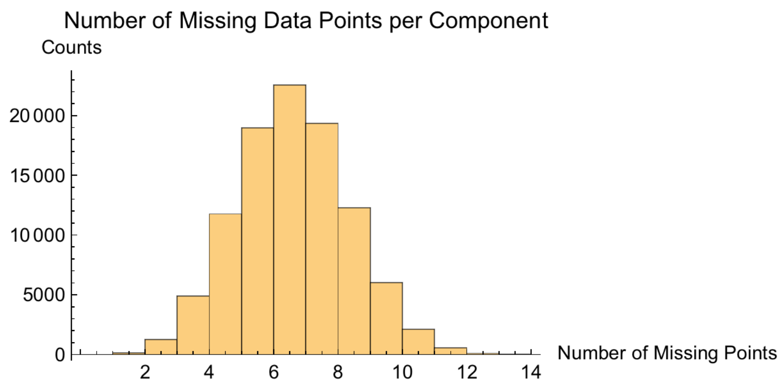

{Missing -> Counts: , <| 1 → 128, 2 → 1268, 3 → 4889, 4 → 11 764, 5 → 18 985, 6 → 22 552, 7 → 19 334, 8 → 12 267, 9 → 6025, 10 → 2112, 11 → 563, 12 → 100, 13 → 13 |> }

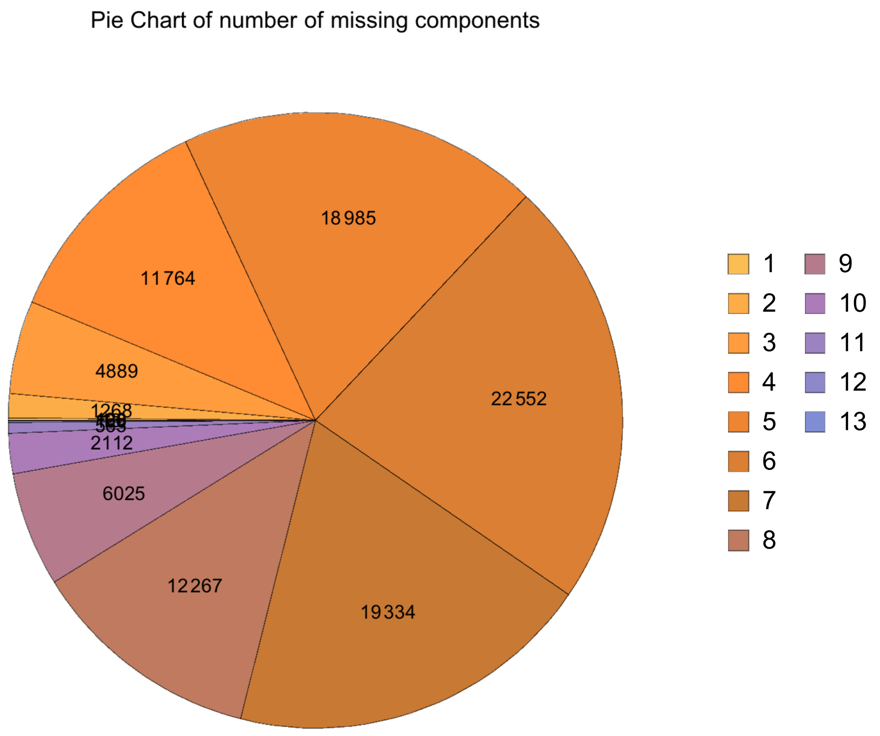

Out[66]=

<| 186 →
<| 2 → {{0.0323567}, {4.}}, 3 → {{0.0466534}, {3.}}, 20 → {{0.124963}, {2.}}, 27 → {{-0.130734}, {3.}},
⋯ 6277 ⋯, 94 071 → Missing[], 94 280 → Missing[], 96 918 → Missing[], 98 744 → Missing[] |>,
⋯ 13 ⋯, 329 → <| 2 → Missing[], ⋯ 6283 ⋯, 98 744 → ⋯ 1 ⋯ |> |>

large output | show less | show more | show all | set size limit...

For each bootstrap protein we now extract a time series (list of values):

In[67]:=
```
timeSeriesProteinBootstrap = CreateTimeSeries[filteredProteinBootstrapData]
```

Out[67]=
```
<|2 → {0.0323567, 0, 0.0178067, 0.0177744, 0.140317, -0.129398, Missing[],
  0.203463, Missing[], 0.288878, -0.169866, Missing[], -0.386924, 0.103362, 0.0735377},
 ··· 6283 ···, 98744 → {Missing[], 0, Missing[], 0.0187488, 0.256245, 0.236313, 0.169999,
  0.0284025, 0.114743, 0.393487, -0.503409, Missing[], 0.272097, 0.0390792, -0.22122}|>
```

large output | show less | show more | show all | set size limit...

Next, we normalize each protein series, using `SeriesApplier`:

In[68]:=
```
normedProteinBootstrapAll = SeriesApplier[Normalize, timeSeriesProteinBootstrap]
```

Out[68]=
```
<|2 → {0.0541072, 0., 0.0297765, 0.0297225, 0.23464, -0.216381, Missing[],
  0.340234, Missing[], 0.483065, -0.284052, Missing[], -0.647019, 0.172843, 0.122971},
 ··· 6283 ···, 98744 → {Missing[], 0., Missing[], 0.0224495, 0.306823, 0.282957, 0.203553,
  0.0340086, 0.137391, 0.471155, -0.602773, Missing[], 0.325805, 0.0467927, -0.264885}|>
```

large output | show less | show more | show all | set size limit...

Finally, we use `ConstantSeriesClean` to remove constant series, as we are interested in changing time patterns:

In[69]:=
```
proteinBootstrapFinalTimeSeries = ConstantSeriesClean[normedProteinBootstrapAll]
```

Out[69]=
```
<| ··· 1 ··· |>
```

large output | show less | show more | show all | set size limit...

Classification of Proteome Time Series

In this subsection we will classify the proteome time series based on patterns in the series. For the classification we will use `TimeSeriesClassification`. We will use `QuantileEstimator` for the "LombScargle" method to provide a cutoff for the `TimeSeriesClassification` inputs.

First, we estimate for the "LombScargle" Method, 0.95 quantile cutoff from the bootstrap proteome data:

In[70]:=
```
q95Protein = QuantileEstimator[proteinBootstrapFinalTimeSeries, timesProteinBootstrap]
```

Out[70]=
```
0.836405
```

Next, we estimate the "Spikes" 0.95 quantile cutoff from the bootstrap proteome data:

In[71]:=
```
q95ProteinSpikes =
  QuantileEstimator[proteinBootstrapFinalTimeSeries, timesProteinBootstrap, Method → "Spikes"]
```

Out[71]=
```
<|12 → {0.819506, -0.832884}, 13 → {0.791436, -0.820784}, 14 → {0.796826, -0.787358}|>
```

Now we can classify the proteome time series data based on these cutoffs:

```
In[72]:= proteinClassification = TimeSeriesClassification[proteinFinalTimeSeries,
   timesProtein, LombScargleCutoff → q95Protein, SpikeCutoffs → q95ProteinSpikes]
```

Method → "LombScargle"

```
Out[72]= <|SpikeMax → <| ··· 1 ··· |>, ··· 4 ···,
  f7 → <| {O14579, Protein} → {{0.129387, 0.0330284, 0.0764435, 0.001172, 0.187055, 0.279572, 0.929071},
      { ··· 1 ··· }}, ··· 16 ···, {Q9HC38, Protein} → { ··· 1 ··· } |> |>

large output   show less   show more   show all   set size limit...
```

As discussed above, the default output for `TimeSeriesClassification` is an `Association` with outer keys being the classification classes, inner keys being the class members, and each class member value being a list of `{{computed classification vector}, {input data list}}`.

If we want the classes produced, we can query the keys:

```
In[73]:= Keys[proteinClassification]
Out[73]= {SpikeMax, SpikeMin, f1, f5, f6, f7}
```

For the number of members in each class we have:

```
In[74]:= Query[All, Length]@proteinClassification
Out[74]= <|SpikeMax → 108, SpikeMin → 75, f1 → 76, f5 → 6, f6 → 36, f7 → 18|>
```

We can obtain the membership list in any class of interest:

```
In[75]:= Query["f1", Keys]@proteinClassification
Out[75]= {{O00160, Protein}, {O00267, Protein}, {O00273, Protein}, {O00571, Protein},
  {O15031, Protein}, {O43143, Protein}, {O43175, Protein}, {O43312, Protein},
  {O43516, Protein}, {O60271, Protein}, {O60879, Protein}, {O75643, Protein},
  {O75792, Protein}, {O95498, Protein}, {P00488, Protein}, {P00915, Protein},
  {P02042, Protein}, {P02671, Protein}, {P04844, Protein}, {P08174, Protein}, {P09326, Protein},
  {P09496, Protein}, {P11021, Protein}, {P12956, Protein}, {P13501, Protein}, {P13611, Protein},
  {P13667, Protein}, {P19387, Protein}, {P23141, Protein}, {P23368, Protein}, {P32119, Protein},
  {P32189, Protein}, {P33176, Protein}, {P40306, Protein}, {P42892, Protein}, {P50225, Protein},
  {P51531, Protein}, {P52888, Protein}, {P54920, Protein}, {P55036, Protein}, {P60660, Protein},
  {P84095, Protein}, {Q01518, Protein}, {Q07021, Protein}, {Q08722, Protein}, {Q09666, Protein},
  {Q13151, Protein}, {Q13217, Protein}, {Q13488, Protein}, {Q14165, Protein}, {Q14653, Protein},
  {Q15084, Protein}, {Q5H9R7, Protein}, {Q6NYC8, Protein}, {Q709C8, Protein}, {Q86YP4, Protein},
  {Q92499, Protein}, {Q96AT9, Protein}, {Q96L92, Protein}, {Q96RT1, Protein}, {Q99439, Protein},
  {Q9BTE3, Protein}, {Q9BTV4, Protein}, {Q9BWS9, Protein}, {Q9C0I1, Protein}, {Q9H0D6, Protein},
  {Q9H2U2, Protein}, {Q9H444, Protein}, {Q9H4Z3, Protein}, {Q9NS69, Protein}, {Q9NUP9, Protein},
  {Q9NVJ2, Protein}, {Q9NYB0, Protein}, {Q9UQ35, Protein}, {Q9Y277, Protein}, {Q9Y2Q0, Protein}}
```

To obtain the possible frequencies we simply run `LombScargle` over the desired times for one of the time series and set the `FrequenciesOnly` option to `True`:

```
In[76]:= LombScargle[proteinFinalTimeSeries[[1]], timesRNA, FrequenciesOnly → True]
Out[76]= <|f1 → 0.00500668, f2 → 0.0104306, f3 → 0.0158545,
  f4 → 0.0212784, f5 → 0.0267023, f6 → 0.0321262, f7 → 0.0375501|>
```

Metabolomic Data

Importing OmicsObject Metabolome Data

We now import the metabolomics data example (for details on how to import such data please refer to `DataImporter`, `DataImporterDirect`, `DataImporterDirectLabeled` and `OmicsObjectCreator` documentation).

We import the metabolomics OmicsObject MathIOmica examples for each of positive and negative mass spectrometry aligned mass features:

```
In[77]:= metabolitesNegativeModeExample =
  Get[FileNameJoin[{ConstantMathIOmicaExamplesDirectory, "metabolomicsNegativeModeExample"}]]
```

```
Out[77]= <|8 -> <|{457.002, 0.34764, Meta} ->
     {{23444, 16317, 1}, { [ C16 H11 N9 S4, db=0.00, overall=47.55, mfg=95.11 ], }},
    ··· 2289 ···, {421.948, 0.392875, Meta} -> {{1, 115528, 130042},
     { [ C11 H12 Cl2 O11 S, db=0.00, overall=48.58, mfg=97.17 ], }}|>, ··· 10 ···, 20 -> <|··· 1 ···|>|>
large output   show less   show more   show all   set size limit...
```

```
In[78]:= metabolitesPositiveModeExample =
  Get[FileNameJoin[{ConstantMathIOmicaExamplesDirectory, "metabolomicsPositiveModeExample"}]]
```

```
Out[78]= <|8 -> <|{202.033, 0.332607, Meta} -> {{263741, 276622, 337241}, {, }},
    {174.038, 0.334514, Meta} -> {{78435, 88529, 121073}, {, }},
    ··· 3670 ···, {422.34, 14.7601, Meta} -> {{1, 36919, 102737}, {, }}|>,
  9 -> <|··· 1 ···|>, ··· 8 ···, 19 -> <|··· 1 ···|>, 20 -> <|··· 1 ···|>|>
large output   show less   show more   show all   set size limit...
```

There are multiple samples given by the outer associations. We can use Query to get any data. For example we can get the outer keys:

```
In[79]:= Query[Keys]@metabolitesNegativeModeExample
Out[79]= {8, 9, 10, 11, 12, 13, 14, 15, 16, 17, 19, 20}
```

```
In[80]:= Query[Keys]@metabolitesPositiveModeExample
Out[80]= {8, 9, 10, 11, 12, 13, 14, 15, 16, 17, 19, 20}
```

We notice that sample 7, 18 and 21 are missing as there was no sample for these time points. This will be addressed further below.

We can get the intensity data from any sample and entry. For example, the 77th and 155th entries in sample 14:

```
In[81]:= Query["14", {77, 155}]@metabolitesNegativeModeExample
Out[81]= <|{322.089, 0.440241, Meta} ->
   {{31950, 29801, 27440}, {Isosorbide-2-glucuronide [ C12 H18 O10, db=60.03, overall=60.67, mfg=61.31,
       KEGG ID=, CAS ID=29542-01-6 ], 29542-01-6}}, {146.059, 0.742692, Meta} -> {{62667, 1, 60382},
    {Adipic acid [ C6 H10 O4, db=45.74, overall=46.59, mfg=47.44, KEGG ID=, CAS ID=124-04-9 ], 124-04-9}}|>
```

The outer keys correspond to the identified features in the form {mass to charge ratio (m/z), retention time, "Meta"}, i.e. each m/z and retention time has been tagged with a "Meta" label as well to indicate these are metabolomics data. The values of all the keys/IDs correspond to `{{measurements}, {metadata}}`, and in this particular example:

```
{{intensity technical replicate 1, intensity technical replicate 2, intensity technical replicate 3},.
 {Annotations, CAS Number}}
```

We would like to combine the positive and negative mode metabolomics data. We will use EnlargeInnerAssociation :

```
In[82]:= metabolitesExample =
          EnlargeInnerAssociation[{metabolitesNegativeModeExample, metabolitesPositiveModeExample}]

Out[82]= ⟨|8 → ⟨|{457.002, 0.34764, Meta} →
              {{23 444, 16 317, 1}, { [ C16 H11 N9 S4, db=0.00, overall=47.55, mfg=95.11 ], }},
            ⋯5962⋯, {422.34, 14.7601, Meta} → {{1, 36 919, 102 737}, {, }}|⟩, ⋯10⋯,
          20 → ⟨|{457.002, 0.34764, Meta} → {{⋯1⋯}, ⋯1⋯}, ⋯5962⋯, ⋯1⋯|⟩|⟩

large output | show less | show more | show all | set size limit...
```

Processing of Metabolome Data

We will next preprocess the imported metabolome data. We will first perform calculate the median of the technical replicates, transform the data towards a normal distribution, then we will re-label the samples with real time and carry out filtering for missing data. Finally, we will create the metabolomics time series or relative intensities compared to the healthy reference point for each mass feature identified.

Medians of Technical Triplicates, Data Transformation, Labeling, Filtering, Matching Mass

Median of Technical Triplicates

The metabolomics intensities have three measurements, corresponding to technical triplicates. Typically we would like to use the median of these values. An additional complication is that some of the triplicates have intensity values of 1, which should be taken as a Missing value. We can use MeasurementApplier to perform the calculation:

MeasurementApplier[*function*, *omicsObject*]	applies a *function* to the measurement list of an *omicsObject*, ignoring missing values.

Applying a function to the measurements in an OmicsObject.

option name	default value	
ComponentIndex	All	ComponentIndex is an option for MathIOmica functions, such as Applier, that allows selection of which component of a list to use in an association or OmicsObject input or output values.
IgnorePattern	_Missing	IgnorePattern is an option for MeasurementApplier specifying a pattern of values to delete prior to applying the function to the measurement list.
ListIndex	1	ListIndex is an option for MathIOmica functions, such as Applier that allows selection of which list to use in the association or OmicsObject input or output values.

Options for MeasurementApplier.

We implement a Median calculation, and ignoring entries with missing and values of 1:

In[83]:=
```
metaboliteMedians = MeasurementApplier[Median, metabolitesExample, IgnorePattern → (_Missing | 1 | 1.)]
```

Out[83]=
```
<|8 →
  <|{457.002, 0.34764, Meta} → {{19880.5}, { [ C16 H11 N9 S4, db=0.00, overall=47.55, mfg=95.11 ], }},
   ··· 5962 ···, {422.34, 14.7601, Meta} → {{69828.}, {, }}|>, ··· 10 ···,
 20 → <|{457.002, 0.34764, Meta} → {{16606.5}, {··· 1 ···}}, ··· 5962 ···, {··· 1 ···} → ··· 1 ···|> |>
```

large output | show less | show more | show all | set size limit...

Data Power Transformation

We apply a Box-Cox transformation to the metabolite median data in the OmicsObject, which is now the first list first component for each identifier. The optimized $\hat{\lambda}$ parameter for each sample is printed out for reference:

In[84]:=
```
transformedMetaboliteData = ApplyBoxCoxTransform[metaboliteMedians, ListIndex → 1, ComponentIndex → 1]
```

Calculated Box-Cox parameter $\hat{\lambda}$ = −0.288857

Calculated Box-Cox parameter $\hat{\lambda}$ = −0.282374

Calculated Box-Cox parameter $\hat{\lambda}$ = −0.276202

Calculated Box-Cox parameter $\hat{\lambda}$ = −0.262075

Calculated Box-Cox parameter $\hat{\lambda}$ = −0.271308

Calculated Box-Cox parameter $\hat{\lambda}$ = −0.27703

Calculated Box-Cox parameter $\hat{\lambda}$ = −0.295395

Calculated Box-Cox parameter $\hat{\lambda}$ = −0.264833

Calculated Box-Cox parameter $\hat{\lambda}$ = −0.278556

Calculated Box-Cox parameter $\hat{\lambda}$ = −0.269513

Calculated Box-Cox parameter $\hat{\lambda}$ = −0.265784

Calculated Box-Cox parameter $\hat{\lambda}$ = −0.262769

Out[84]=
```
<|8 →
  <|{457.002, 0.34764, Meta} → {{3.26345}, { [ C16 H11 N9 S4, db=0.00, overall=47.55, mfg=95.11 ], }},
   ··· 5962 ···, {422.34, 14.7601, Meta} → {{3.32386}, {, }}|>,
 ··· 10 ···, 20 → <|{457.002, 0.34764, Meta} → {··· 1 ···}, ··· 5962 ···, ··· 1 ···|> |>
```

large output | show less | show more | show all | set size limit...

We can plot the data to see what the resulting distributions look like:

In[85]:=
```
Histogram[#] & /@ (Query[All, Values, 1, 1]@transformedMetaboliteData)
```

Out[85]=

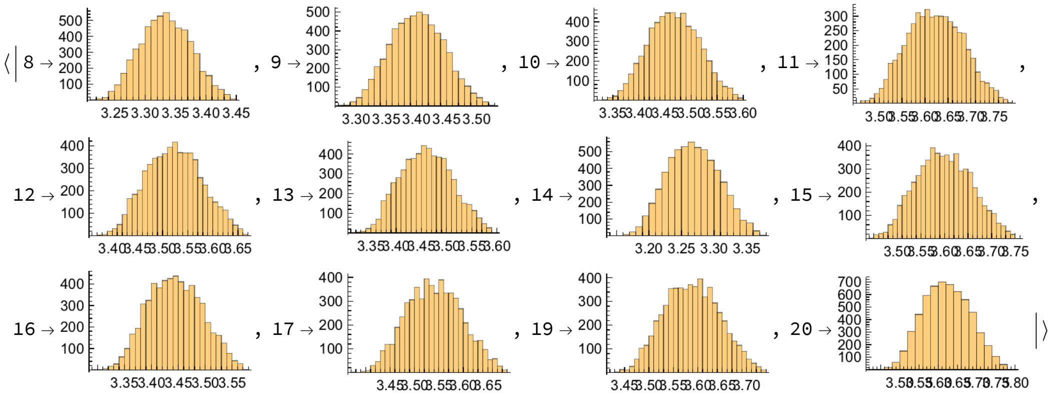

We may also wish to standardize the distributions:

In[86]:=
```
metabolitesStandardized =
 Returner[transformedMetaboliteData, Applier[StandardizeExtended[#, Mean, StandardDeviation] &,
   transformedMetaboliteData, ListIndex → 1, ComponentIndex → 1], ListIndex → 1, ComponentIndex → 1]
```

Out[86]=
```
<|8 →
  <|{457.002, 0.34764, Meta} → {{-1.71178}, { [ C16 H11 N9 S4, db=0.00, overall=47.55, mfg=95.11 ], }},
   ··· 5962 ···, {422.34, 14.7601, Meta} → {{-0.247328}, {, }}|>,
 ··· 10 ···, 20 → <|{457.002, 0.34764, Meta} → {··· 1 ···}, ··· 5962 ···, ··· 1 ···|>|>
```

large output | show less | show more | show all | set size limit...

We can again plot the data to see what the standardized distributions look like:

In[87]:=
```
Histogram[#] & /@ (Query[All, Values, 1, 1]@metabolitesStandardized)
```

Out[87]=

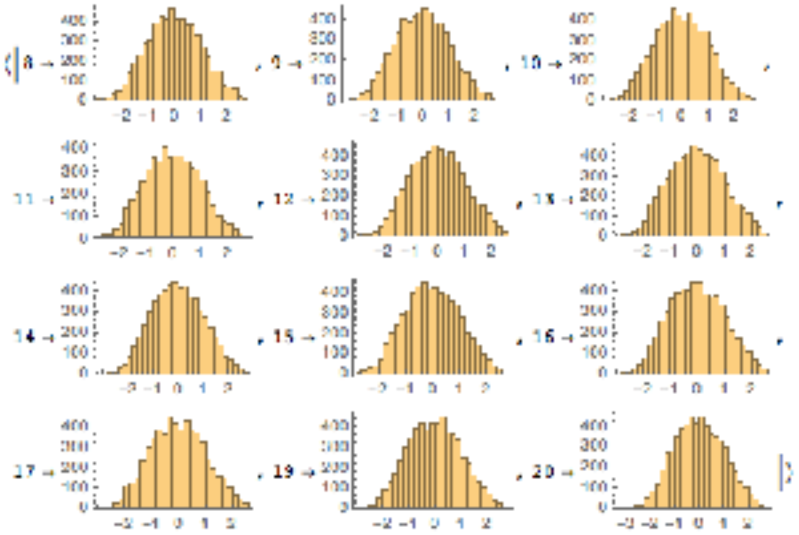

Re-labeling Samples with Times

As with the transcriptome, we notice that the sample numberings do not correspond to actual days, so we may adjust using the sampleToDays association created above:

In[88]:=
```
sampleToDays =
  <|"7" → "186", "8" → "255", "9" → "289", "10" → "290", "11" → "292", "12" → "294", "13" → "297", "14" → "301",
   "15" → "307", "16" → "311", "17" → "322", "18" → "329", "19" → "369", "20" → "380", "21" → "400"|>;
```

We can now do a KeyMap to rename the outer keys:

In[89]:=
```
metabolitesLongitudinal = KeyMap[sampleToDays, metabolitesStandardized]
```

Out[89]=
```
<|255 →
   <|{457.002, 0.34764, Meta} → {{-1.71178}, { [ C16 H11 N9 S4, db=0.00, overall=47.55, mfg=95.11 ], }},
    ··· 5962 ···, {422.34, 14.7601, Meta} → {{-0.247328}, {, }}|>,
  ··· 10 ···, 380 → <|{457.002, 0.34764, Meta} → {··· 1 ···}, ··· 5962 ···, ··· 1 ···|>|>
```
large output | show less | show more | show all | set size limit...

Now let's check the timepoints in this dataset:

In[90]:=
```
timesMetaboliteRawData = TimeExtractor[metabolitesLongitudinal]
```

Out[90]=
```
{255, 289, 290, 292, 294, 297, 301, 307, 311, 322, 369, 380}
```

We notice a complication: there are three timepoints missing, corresponding to the three samples for which we had indicated above that there were no measurements (compared to the transcriptome samples). These are samples on days "186", "329" and "400".

We can use the `ConstantAssociator` function to append these to the transformed data, tagging these data as Missing data:

In[91]:=
```
metabolitesLongitudinalEnlarged =
 ConstantAssociator[metabolitesLongitudinal, <|"186" → Missing[], "329" → Missing[], "400" → Missing[]|>]
```

Out[91]=
```
<|255 →
   <|{457.002, 0.34764, Meta} → {{-1.71178}, { [ C16 H11 N9 S4, db=0.00, overall=47.55, mfg=95.11 ], }},
    ··· 5962 ···, {422.34, 14.7601, Meta} → {{-0.247328}, {, }}|>,
  ··· 13 ···, 400 → <|{457.002, 0.34764, Meta} → ··· 1 ···, ··· 5962 ···, ··· 1 ···|>|>
```
large output | show less | show more | show all | set size limit...

We can now check the timepoints again:

In[92]:=
```
timesMetabolites = TimeExtractor[metabolitesLongitudinalEnlarged]
```

Out[92]=
```
{186, 255, 289, 290, 292, 294, 297, 301, 307, 311, 322, 329, 369, 380, 400}
```

Filter Data

We will next remove values that have been tagged overall as Missing[], retaining data that have at least 3/4 data points available across all samples. Additionally we remove data where the reference healthy sample "255" was missing. We use the function `FilterMissing` for this implementation:

In[93]:=
```
filteredMetaboliteData = FilterMissing[metabolitesLongitudinalEnlarged, 3/4, Reference → "255"]
```

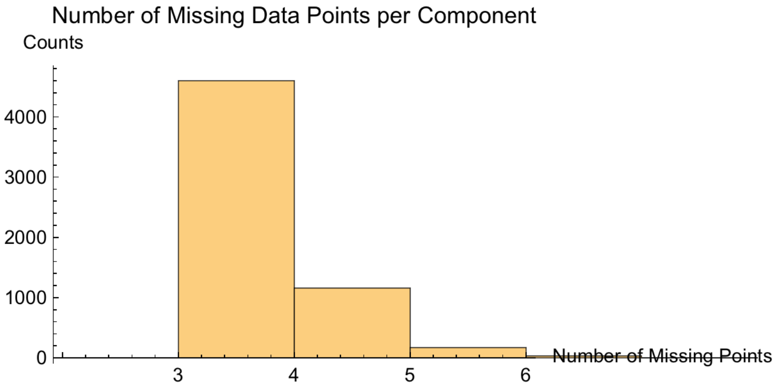

{Missing -> Counts: , <|3 → 4601, 4 → 1158, 5 → 172, 6 → 31, 7 → 2|>}

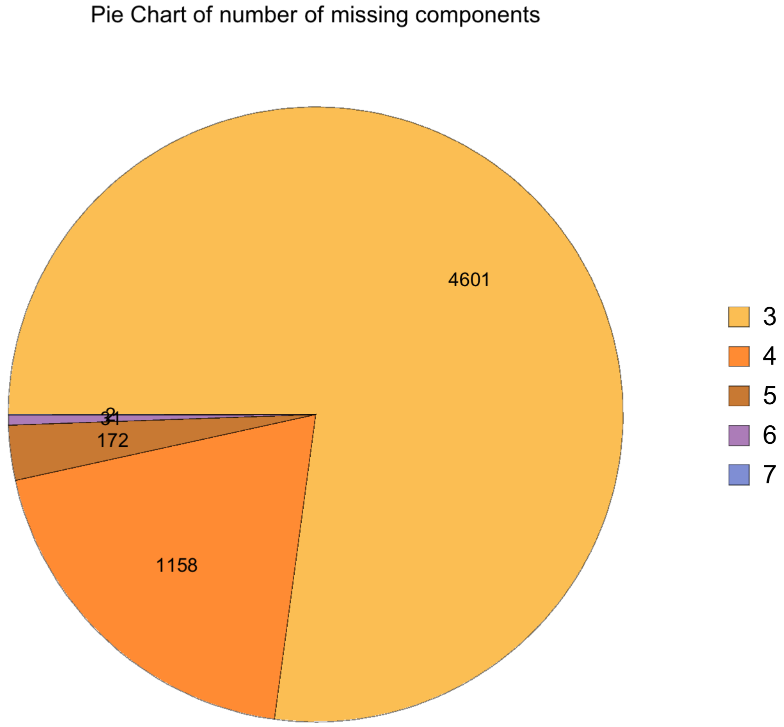


```
Out[93]=
<|255 →
  <|{457.002, 0.34764, Meta} → {{-1.71178}, { [ C16 H11 N9 S4, db=0.00, overall=47.55, mfg=95.11 ], }},
  ··· 4599 ···, {406.381, 14.5609, Meta} →
   {{-1.34842}, {2,4,6-trimethyl-2,15 … Lipid ID=, KEGG ID= ], }}|>, ··· 13 ···, 400 → <|··· 1 ···|>|>
large output | show less | show more | show all | set size limit...
```

Matching Unique Mass

We may want to match a unique mass to the metabolites. This is a putative mass identification based on the uniqueness of the mass feature. If matched, a KEGG compound identity can be prepended to the identifier using `OmicsObjectUniqueMassConverter`.

OmicsObjectUniqueMassConverter[*omicsObject*, *massAccuracy*]	assigns a unique putative mass identification to each of *omicsObject*'s inner association keys, using the *massAccuracy* in parts per million.

Matching putative mass identifications to mass features in an OmicsObject of metabolites.

We match our identities to KEGG compound identifiers, using a 2ppm accuracy (this may take some time depending on the number of matching data):

```
In[94]:= massMatchedFilteredMetabolites = OmicsObjectUniqueMassConverter[filteredMetaboliteData, 2]

Out[94]= <|255 →
  <|{457.002, 0.34764, Meta} → {{-1.71178}, { [ C16 H11 N9 S4, db=0.00, overall=47.55, mfg=95.11 ], }},
   ··· 4599 ···, {406.381, 14.5609, Meta} →
    {{-1.34842}, {2,4,6-trimethyl-2,15 … Lipid ID=, KEGG ID= ], }}|>, ··· 13 ···, 400 → <|··· 1 ···|> |>
large output | show less | show more | show all | set size limit...
```

Create Metabolome Time Series

We can now create time series for each of the proteins.

For each metabolite feature we now extract a time series (list of values) corresponding to the set of times:

```
In[95]:= timeSeriesMetabolites = CreateTimeSeries[massMatchedFilteredMetabolites]

Out[95]= <|{457.002, 0.34764, Meta} → {Missing[], -1.71178, -2.03844, -1.95662, -1.68101, -1.82463, ··· 3 ···,
   -2.35258, -1.87739, Missing[], -2.05224, -1.85568, Missing[]}, ··· 4599 ···, {··· 1 ···} → ··· 1 ···|>
large output | show less | show more | show all | set size limit...
```

Take Difference Compared to Reference in Metabolome Time Series.

Now we need to compare to compare the difference of each intensity for a given metabolite's time series to the intensity of the ratios of expression at any time point compared to a healthy datapoint. We can use the function SeriesInternalCompare:

We compare every value in each series to the healthy "255" time point, which is the second element in each series:

```
In[96]:= metabolitesCompared = SeriesInternalCompare[timeSeriesMetabolites, ComparisonIndex → 2]

Out[96]= <|{457.002, 0.34764, Meta} → {Missing[], 0., -0.326659, -0.244843, 0.0307746, -0.112847, ··· 3 ···,
   -0.640794, -0.165613, Missing[], -0.340455, -0.143904, Missing[]}, ··· 4599 ···, {··· 1 ···} → {··· 1 ···}|>
large output | show less | show more | show all | set size limit...
```

Take the Norm and Remove Constant Metabolome Time Series

Next, we normalize each series, using again `SeriesApplier`:

```
In[97]:= normedMetabolitesCompared = SeriesApplier[Normalize, metabolitesCompared]

Out[97]= <|{457.002, 0.34764, Meta} → {Missing[], 0., -0.343784, -0.25768, 0.032388, -0.118763, … 3 …, -0.674389,
    -0.174295, Missing[], -0.358304, -0.151448, Missing[]}, … 4599 …, {… 1 …} → … 1 … |>
large output   show less   show more   show all   set size limit...
```

Finally, we use `ConstantSeriesClean` to remove constant series, as we are interested in changing time patterns:

```
In[98]:= metabolomeFinalTimeSeries = ConstantSeriesClean[normedMetabolitesCompared]

Out[98]= <|{457.002, 0.34764, Meta} → {Missing[], 0., -0.343784, -0.25768, 0.032388, -0.118763, … 3 …, -0.674389,
    -0.174295, Missing[], -0.358304, -0.151448, Missing[]}, … 4599 …, {… 1 …} → … 1 … |>
large output   show less   show more   show all   set size limit...
```

Resampling Metabolome Data

We also would like to create a resampled distribution for the metabolome dataset prior to classification and clustering. In this subsection we first resample the imported metabolome dataset. Then, we carry out the full analysis in this "bootstrap" dataset, to create a set of random metabolome time series. This bootstrap distribution of time series will be used to provide the cutoffs used in the time series classification in the following subsection.

Resampling the Proteome Data

We create a resampling of 100000 sets:

```
In[99]:= metabolitesBootstrap = BootstrapGeneral[metabolitesExample, 100 000]

Out[99]= <|8 → <|1 → {{59 123, 52 730, 68 584},
      {Benzoquinoneacetic acid [ C8 H6 O4, db=86.34, overall=43.17, HMP ID=HMDB02334, KEGG ID= ], }},
    … 99 998 …, 100 000 → {{76 318, 59 290, 44 033},
      {16-phenyl-tetranor-PGE2 [ C22 … id ID=LMFA03010066, KEGG ID= ], }}|>, … 10 …, 20 → <|… 1 …|>|>
large output   show less   show more   show all   set size limit...
```

Processing the Bootstrap Metabolome and Creating Bootstrap Time Series

We implement a Median calculation, and ignoring entries with missing and values of 1 for the bootstrap set:

```
In[100]:= metaboliteBootstrapMedians =
  MeasurementApplier[Median, metabolitesBootstrap, IgnorePattern → (_Missing | 1 | 1.)];
```

We apply a Box-Cox transformation to the bootstrap metabolite median data in the OmicsObject, which is now the first list first component for each identifier. The optimized $\hat{\lambda}$ parameter for each sample is printed out for reference:

```
In[101]:= transformedBootstrapMetaboliteData =
 ApplyBoxCoxTransform[metaboliteBootstrapMedians, ListIndex → 1, ComponentIndex → 1]
```

```
Calculated Box-Cox parameter λ̂ = -0.288728
Calculated Box-Cox parameter λ̂ = -0.279522
Calculated Box-Cox parameter λ̂ = -0.276162
Calculated Box-Cox parameter λ̂ = -0.26296
Calculated Box-Cox parameter λ̂ = -0.269051
Calculated Box-Cox parameter λ̂ = -0.277505
Calculated Box-Cox parameter λ̂ = -0.294353
Calculated Box-Cox parameter λ̂ = -0.264964
Calculated Box-Cox parameter λ̂ = -0.280633
Calculated Box-Cox parameter λ̂ = -0.268157
Calculated Box-Cox parameter λ̂ = -0.267766
Calculated Box-Cox parameter λ̂ = -0.260673
```

Out[101]=
```
<|8 → <|1 → {{3.31833},
    {Benzoquinoneacetic acid [ C8 H6 O4, db=86.34, overall=43.17, HMP ID=HMDB02334, KEGG ID= ], }},
  2 → {⋯ 1 ⋯}, ⋯ 99 996 ⋯, 99 999 → ⋯ 1 ⋯, 100 000 →
   {{3.31845}, {16-phenyl-tetranor-PGE2 [ C22 … d ID=LMFA03010066, KEGG ID= ], }}|>, ⋯ 10 ⋯, ⋯ 1 ⋯|>
```
large output | show less | show more | show all | set size limit...

We may also wish to standardize the distributions:

In[102]:=
```
metabolitesBootstrapStandardized = Returner[transformedBootstrapMetaboliteData,
  Applier[StandardizeExtended[#, Mean, StandardDeviation] &, transformedBootstrapMetaboliteData,
   ListIndex → 1, ComponentIndex → 1], ListIndex → 1, ComponentIndex → 1]
```

Out[102]=
```
<|8 → <|1 → {{-0.409196},
    {Benzoquinoneacetic acid [ C8 H6 O4, db=86.34, overall=43.17, HMP ID=HMDB02334, KEGG ID= ], }},
  2 → {⋯ 1 ⋯}, ⋯ 99 996 ⋯, 99 999 → ⋯ 1 ⋯, 100 000 →
   {{-0.406328}, {16-phenyl-tetranor-PGE2 [ C … ID=LMFA03010066, KEGG ID= ], }}|>, ⋯ 10 ⋯, ⋯ 1 ⋯|>
```
large output | show less | show more | show all | set size limit...

We can now do a KeyMap to rename the outer keys with labels corresponding to days:

In[103]:=
```
metabolitesBootstrapLongitudinal = KeyMap[sampleToDays, metabolitesBootstrapStandardized];
```

Now let's check the timepoints in this dataset:

In[104]:=
```
timesMetaboliteBootstrapData = TimeExtractor[metabolitesBootstrapLongitudinal]
```

Out[104]=
```
{255, 289, 290, 292, 294, 297, 301, 307, 311, 322, 369, 380}
```

We can use the `ConstantAssociator` function to append the "186", "329" and "400" missing days to the transformed bootstrap data:

In[105]:=
```
metabolitesBootstrapLongitudinalEnlarged = ConstantAssociator[
  metabolitesBootstrapLongitudinal, <|"186" → Missing[], "329" → Missing[], "400" → Missing[]|>];
```

We can now check the timepoints again:

```
In[106]:= timesMetabolitesBootstrap = TimeExtractor[metabolitesBootstrapLongitudinalEnlarged]
Out[106]= {186, 255, 289, 290, 292, 294, 297, 301, 307, 311, 322, 329, 369, 380, 400}
```

We next remove values that have been tagged overall as Missing[], retaining data that have at least 3/4 data points available across all samples. Additionally we remove data where the reference healthy sample "255" was missing. We use the function `FilterMissing` for this implementation:

```
In[107]:= filteredMetaboliteBootstrapData =
            FilterMissing[metabolitesBootstrapLongitudinalEnlarged, 3/4, Reference → "255"];
```

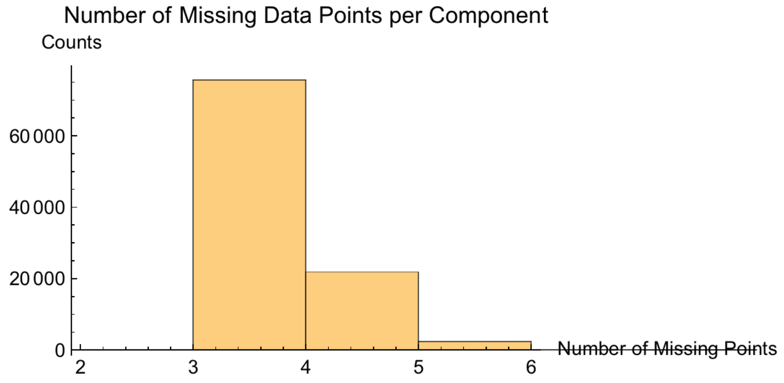

{Missing -> Counts: , <|3 → 75 675, 4 → 21 845, 5 → 2344, 6 → 130, 7 → 6|>}

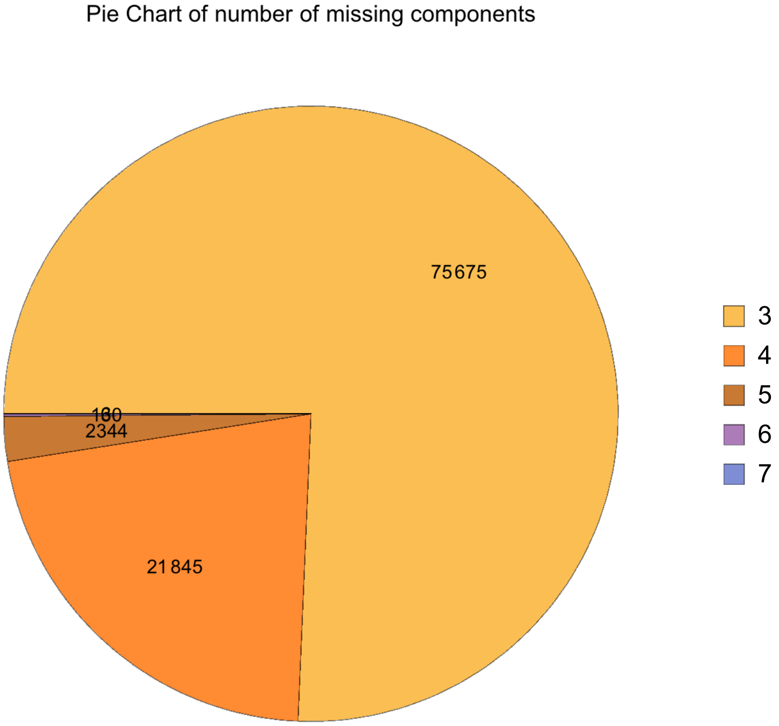

For each bootstrap metabolite feature we now extract a time series (list of values) corresponding to the set of times:

```
In[108]:= timeSeriesMetabolitesBootstrap = CreateTimeSeries[filteredMetaboliteBootstrapData];
```

We compare every value in each bootstrap series to the healthy "255" time point, which is the second element in each series:

In[109]:=
```
metabolitesBootstrapCompared = SeriesInternalCompare[timeSeriesMetabolitesBootstrap, ComparisonIndex → 2];
```

Next, we normalize each series, using again `SeriesApplier`:

In[110]:=
```
normedMetabolitesBootstrapCompared = SeriesApplier[Normalize, metabolitesBootstrapCompared];
```

Finally, we use `ConstantSeriesClean` to remove constant series, as we are interested in changing time patterns:

In[111]:=
```
metabolomeBootstrapFinalTimeSeries = ConstantSeriesClean[normedMetabolitesBootstrapCompared];
```

Classification of Metabolome Time Series

In this subsection we will classify the meetabolome time series based on patterns in the series. For the classification we will use `TimeSeriesClassification`. We will use `QuantileEstimator` for the "LombScargle" method to provide a cutoff for the `TimeSeriesClassification` inputs.

First, we estimate for the "LombScargle" Method, 0.95 quantile cutoff from the bootstrap metabolome data:

In[112]:=
```
q95Metabolites = QuantileEstimator[metabolomeBootstrapFinalTimeSeries, timesMetabolitesBootstrap]
```

Out[112]=
```
0.846716
```

Next, we estimate the "Spikes" 0.95 quantile cutoff from the bootstrap proteome data:

In[113]:=
```
q95MetabolitesSpikes =
 QuantileEstimator[metabolomeBootstrapFinalTimeSeries, timesMetabolitesBootstrap, Method → "Spikes"]
```

Out[113]=
```
<|12 → {0.669189, -0.651331}|>
```

Now we can classify the proteome time series data based on these cutoffs:

In[114]:=
```
metaboliteClassification = TimeSeriesClassification[metabolomeFinalTimeSeries,
  timesMetabolites, LombScargleCutoff → q95Metabolites, SpikeCutoffs → q95MetabolitesSpikes]
 Method → "LombScargle"
```

Out[114]=
```
<|SpikeMax →
  <|{1514.1, 0.366235, Meta} → {{0.150094, 0.150759, 0.336515, 0.197558, 0.430385, 0.667846, 0.41379},
     {Missing[], 0., ··· 12 ···, Missing[]}}, ··· 138 ···, {··· 1 ···} → ··· 1 ···|>, ··· 6 ···|>
large output | show less | show more | show all | set size limit...
```

As discussed above, the default output for TimeSeriesClassification is an Association with outer keys being the classification classes, inner keys being the class members, and each class member value being a list of {{computed classification vector}, {input data list}}.

If we want the classes produced, we can query the keys:

In[115]:=
```
Keys[metaboliteClassification]
```

Out[115]=
```
{SpikeMax, SpikeMin, f1, f2, f5, f6, f7}
```

For the number of members in each class we have:

```
In[116]:= Query[All, Length]@metaboliteClassification

Out[116]= ⟨|SpikeMax → 140, SpikeMin → 717, f1 → 62, f2 → 38, f5 → 43, f6 → 14, f7 → 33|⟩
```

We can obtain the membership list in any class of interest:

```
In[117]:= Query["f1", Keys]@metaboliteClassification

Out[117]= {{373.859, 0.411324, Meta}, {cpd:C11821, 184.024, 0.653444, Meta}, {221.109, 10.3062, Meta},
 {cpd:C18218, 272.235, 12.7737, Meta}, {294.166, 13.0495, Meta}, {631.385, 13.5221, Meta},
 {563.32, 13.7008, Meta}, {779.604, 13.9622, Meta}, {362.266, 14.001, Meta},
 {cpd:C17873, 384.36, 14.2982, Meta}, {390.297, 14.3592, Meta}, {420.361, 14.6658, Meta},
 {434.376, 14.7796, Meta}, {392.366, 15.0173, Meta}, {394.381, 15.1519, Meta}, {1599.15, 15.281, Meta},
 {693.628, 15.6921, Meta}, {874.715, 15.9118, Meta}, {281.986, 0.390455, Meta}, {504.309, 14.3911, Meta},
 {416.313, 14.4627, Meta}, {735.521, 15.1792, Meta}, {571.961, 0.388167, Meta}, {489.958, 0.388912, Meta},
 {325.95, 0.392472, Meta}, {465.913, 0.393056, Meta}, {383.909, 0.397722, Meta},
 {301.906, 0.407861, Meta}, {219.903, 0.412111, Meta}, {161.944, 0.413086, Meta},
 {139.061, 0.458472, Meta}, {115.064, 0.463972, Meta}, {71.074, 0.482559, Meta}, {253.165, 9.12729, Meta},
 {298.132, 9.30967, Meta}, {cpd:C20605, 411.179, 9.3167, Meta}, {440.201, 11.2909, Meta},
 {355.218, 12.7443, Meta}, {1061.15, 13.0612, Meta}, {210.198, 13.1613, Meta}, {501.367, 13.296, Meta},
 {594.375, 13.3701, Meta}, {1538.03, 13.3796, Meta}, {404.314, 13.6028, Meta}, {692.323, 13.7652, Meta},
 {670.265, 13.8732, Meta}, {814.584, 14.1513, Meta}, {366.349, 14.3015, Meta}, {442.402, 14.3568, Meta},
 {406.381, 14.3581, Meta}, {278.152, 14.364, Meta}, {cpd:C19658, 344.271, 14.4331, Meta},
 {420.358, 14.4446, Meta}, {311.319, 14.6119, Meta}, {791.583, 15.4236, Meta}, {1553.18, 15.4429, Meta},
 {1545.17, 15.5017, Meta}, {352.052, 0.53368, Meta}, {cpd:C17237, 254.073, 12.2926, Meta},
 {336.228, 12.5103, Meta}, {638.402, 13.4139, Meta}, {668.324, 13.988, Meta}}
```

To obtain the possible frequencies we simply run `LombScargle` over the desired times for one of the time series and set the `FrequenciesOnly` option to `True`:

```
In[118]:= LombScargle[metabolomeFinalTimeSeries[[1]], timesMetabolites, FrequenciesOnly → True]

Out[118]= ⟨|f1 → 0.00500668, f2 → 0.0104306, f3 → 0.0158545,
 f4 → 0.0212784, f5 → 0.0267023, f6 → 0.0321262, f7 → 0.0375501|⟩
```

Combined Data Clustering

In this section we will combine the omics data classes from the individual classifications above using `JoinNestedAssociations` and hierarchically cluster the information to obtain a second level of classification using `TimeSeriesClusters`. We will visualize the results in the following section.

Combining Multi-omics Classifed Data

`JoinNestedAssociations[`*associationList*`]`	merges the nested associationList (an association of associations) by joining the inner associations for each matching key.

Joining classification data.

We combine the classification data using `JoinNestedAssociations`:

In[119]:=
```
combinedClassification =
 JoinNestedAssociations[{rnaClassification, proteinClassification, metaboliteClassification}]
```

Out[119]=
```
<|SpikeMax →
  <|{LOC100132287, RNA} → {{0.0915681, 0.545226, 0.0943158, 0.512412, 0.115633, 0.239286, 0.59351},
     {0.797569, 0., ⋯ 11 ⋯, 0., 0.}}, ⋯ 846 ⋯, {⋯ 1 ⋯} → ⋯ 1 ⋯|>, ⋯ 8 ⋯|>
```

large output | show less | show more | show all | set size limit...

We can check the keys before and after the combination:

In[120]:=
```
Keys[#] & /@ {rnaClassification, proteinClassification, metaboliteClassification}
```

Out[120]=
```
{{SpikeMax, SpikeMin, f1, f2, f3, f4, f5, f6, f7},
 {SpikeMax, SpikeMin, f1, f5, f6, f7}, {SpikeMax, SpikeMin, f1, f2, f5, f6, f7}}
```

In[121]:=
```
Keys@combinedClassification
```

Out[121]=
```
{SpikeMax, SpikeMin, f1, f2, f3, f4, f5, f6, f7}
```

We can also check the membership counts before and after the combination:

In[122]:=
```
Query[All, Length]@# & /@ {rnaClassification, proteinClassification, metaboliteClassification}
```

Out[122]=
```
{<|SpikeMax → 600, SpikeMin → 8507, f1 → 58, f2 → 3, f3 → 13, f4 → 40, f5 → 14, f6 → 10, f7 → 56|>,
 <|SpikeMax → 108, SpikeMin → 75, f1 → 76, f5 → 6, f6 → 36, f7 → 18|>,
 <|SpikeMax → 140, SpikeMin → 717, f1 → 62, f2 → 38, f5 → 43, f6 → 14, f7 → 33|>}
```

In[123]:=
```
Query[All, Length]@combinedClassification
```

Out[123]=
```
<|SpikeMax → 848, SpikeMin → 9299, f1 → 196, f2 → 41, f3 → 13, f4 → 40, f5 → 63, f6 → 60, f7 → 107|>
```

Clustering of Classified Data

Now that we have combined the classes for the various omics, we can cluster them together to obtain the various trends using `TimeSeriesClusters`. A two-tier hierarchical clustering of the data is performed, using a set of two classification vectors, {{classification $vector_1$}, {classification $vector_2$}} for each time series to cluster the data pairwise. The vectors are typically the output from `TimeSeriesClassification`. Similarities at each clustering tier are then computed using in succession from each time series first {classification $vector_1$}, and subsequently {classification $vector_2$} (which corresponds to the {input data time series} if the input is from `TimeSeriesClassification`).

The number of groups and subgroups for each tier of clustering is automatically determined by using internally the "Silhouette" (default) or "Gap" as "SignificanceTest" methods (see also Partitioning Data into Clusters).

TimeSeriesClusters[*data*]	performs clustering of time series data using two tiers of hierarchical clustering to identify groups and subgroups in the data. TimeSeriesClusters takes as input series data, where each data is comprised of two lists and performs clustering of the data to identify groups and sub–groups based on similarities between the input series. The form of the input data is either an association of classes and members, where each member must have a list of two components, typically two vectors used in classification: {{classification vector$_1$}, {classification vector$_2$}}. In the most common case of using as input data that came from performing a TimeSeriesClassification, the {classification vector$_2$} will correspond to input original data for the corresponding time series.

Clustering of classified time series.

option name	default value	
ClusterLabeling	""	Additional label to append to each cluster being computed to prepend to the inbuilt G#S# labeling.
DendrogramPlotOptions	{}	Options passed to the DendrogramPlot function used internally to generate the dendrograms.
DistanceFunction	EuclideanDistance	Distance function to be used in calculating the similarities between different time series in the first tier of clustering.
LinkageMeasure	"Average"	Which linkage measure to use in computing fusion coefficients.
PrintDendrograms	False	Option to print dendrograms for the clustering computed.
ReturnDendrograms	False	Option to return the dendrograms as output.
SignificanceCriterion	"Silhouette"	Method used in determining the number of groups and subgroups at each tier of clustering.
SingleAssociationLabel	"1"	Label to use in case a list is provided to name the class of data produced.
SubclusteringDistanceFunction	EuclideanDistance	Distance function to be used in calculating the similarities between different time series in the second tier of clustering.

Options for TimeSeriesClusters .

The output of TimeSeriesClusters is always an association of associations, providing a summary of the two tier clustering results for each class provided in the input. The output has the form:

```
output =
 <| Class_1 →  <|"Cluster"→ cluster object_1,
    "InitialSplitCluster"→  {InitialSplitCluster_11, InitialSplitCluster_12 ...},
    "IntermediateClusters"→  {IntermediateCluster_11, IntermediateCluster_12 ...},
    "SubsplitClusters"→  {{SubsplitClusters_11} {SubsplitClusters_12}},
    "Data"→  {{input data vector_11}→ Member_11,  ...,},
    "GroupAssociations"→  <|"G1S1"→{member list G1S1},
       "G1S2"→ {member list for G1S2},
       ...,
       "G2S1"→{ ...}|>|>,
  Class_2 →  <|"Cluster"→ cluster object_2,
    "InitialSplitCluster"→  {InitialSplitCluster_21, InitialSplitCluster_22 ...},
    "IntermediateClusters"→  {IntermediateCluster_21, IntermediateCluster_22 ...},
    "SubsplitClusters"→  {{SubsplitClusters_21} {SubsplitClusters_22}},
    "Data"→  {{input data vector_21}→ Member_21,  ...,},
    "GroupAssociations"→  <|"G1S1"→{member list G1S1},
       "G1S2"→ {member list for G1S2},
       ...,
       "G2S1"→{ ...}|>|>,
  ...,
  Class_M →  <|"Cluster"→ cluster object_M,
    "InitialSplitCluster"→  {InitialSplitCluster_M1, InitialSplitCluster_M2 ...},
    "IntermediateClusters"→  {IntermediateCluster_M1, IntermediateCluster_M2 ...},
    "SubsplitClusters"→  {{subsplitClusters_M1} {subsplitClusters_M2}},
    "Data"→  {{input data vector_M1}→ Member_M1,  ...,},
    "GroupAssociations"→  <|"G1S1"→{member list G1S1},
       "G1S2"→ {member list for G1S2},
       ...,
       "G2S1"→{ ...}|>|>
 |>
```

Method	Description
"Cluster"	Cluster generated using the input {classification $vector_1$} for similarity calculations.
"InitialSplitCluster"	Clusters resulting from splitting the initial cluster (reported by key "Cluster") into groups using the SignificanceCriterion to determine the number of clusters.
"IntermediateClusters"	Aglomerative clustering result of hierarchical clustering of each of the initial split clusters (reported by "InitialSplitCluster")
"SubsplitClusters"	Custers generated from splitting the clusters following the second tier clustering (reported by "IntermediateClusters") into subgroups using the SignificanceCriterion to determine the number of clusters.
"Data"	Data reported in the order of clustering results as rules of {classification $vector_2$}→ label for each time series, sorted in order of the clustering results.
"GroupAssociations"	Association denoting membership of each initial data label to groups and subgroups generated by the two tier clustering.

Output keys for TimeSeriesClusters provide clustering information.

We now cluster our combined data (a printout of the clusters is included as a default option):

In[124]:=
```
combinedClusters = TimeSeriesClusters[combinedClassification]
```

Agglomerate::ties : 226 ties have been detected; reordering input may produce a different result. >>

Agglomerate::ties : 1 ties have been detected; reordering input may produce a different result. >>

Agglomerate::ties : 1 ties have been detected; reordering input may produce a different result. >>

General::stop : Further output of Agglomerate::ties will be suppressed during this calculation. >>

Out[124]=
```
<|SpikeMax → <|Cluster → Cluster[Cluster[Cluster[ … 1 … , … 3 … , 19],
      Cluster[Cluster[Cluster[ … 1 … ], Cluster[ … 1 … ], 0.735647, 142, 183], … 3 … , 110],
      0.795876, 153, 435], Cluster[ … 1 … ], … 19 … , 588, 260], … 4 … , … 1 … |>, … 8 … |>
```

large output | show less | show more | show all | set size limit...

Visualization

After our data have been clustered, we would like to visuzlie the results in heatmaps and dendrograms. For the two-tier clustering we have performed MathIOmica can output all the clusterings in labeled dendrograms and heatmaps using `TimeSeriesDendrogramsHeatmaps`, which iteratively calls `TimeSeriesDendrogramHeatmap` on each class.

`TimeSeriesDendrogramsHeatmaps` [*data*]	generates dendrograms and associated heatmap plots for clustered time series data, typically the output of all classes generated by implementing `TimeSeriesClusters`.
`TimeSeriesDendrogramHeatmap` [*data*]	generates a dendrogram and heatmap plot for one set of time series *data* clusters, typically the output of a single class of `TimeSeriesClusters`.

Visualizing the results of classification.

option name	default value	
FunctionOptions	{ImageSize -> 200}	Options list passed to the internal `TimeSeriesDendrogramHeatmap` function.

Options for `TimeSeriesDendrogramsHeatmaps`.

option name	default value	
ColorBlending	{CMYKColor[1, 0, 1, 0], CMYKColor[0, 1, 1, 0]}	Color scheme for the plot. The color list is passed to an internal Blend function to create a ColorFunction for an internal ArrayPlot function.
DendrogramColor	RGBColor[1, 1, 0]	Color to highlight the dendrograms.
FrameName	"Dendrogram and Heatmap"	Label for plot frame.
GroupSubSize	{0.1, 0.1}	Relative size of group and subgroup reference column in plot.
HorizontalAxisName	"Time (arbitrary units)"	Label for the horizontal heatmap axis.
HorizontalLabels	None	Labels for horizontal axis for each column.
IndexColor	"DeepSeaColors"	Choice of color for labeling the group/subgroup index.
ImageSize	200	ImageSize is an option that specifies the overall size of an image to display for an object.
ScaleShift	None	Option to reset the blend of the colors used overall. The option is a real positive number, and is used as a multiplier for an internal Blend function's second argument.
VerticalLabels	None	Labels for vertical axis for each row.

Options for TimeSeriesDendrogramHeatmap .

For each class a separate plot is generated: dendrograms are represented on the left, and are highlighted to represent the grouping level. The G, S, columns represent the groupings and subgroupings generated by the clustering. The legend shows the corresponding groupings and subgrouping, and the number of elements in each group subgroup.

In[125]:=
```
TimeSeriesDendrogramsHeatmaps[combinedClusters]
```

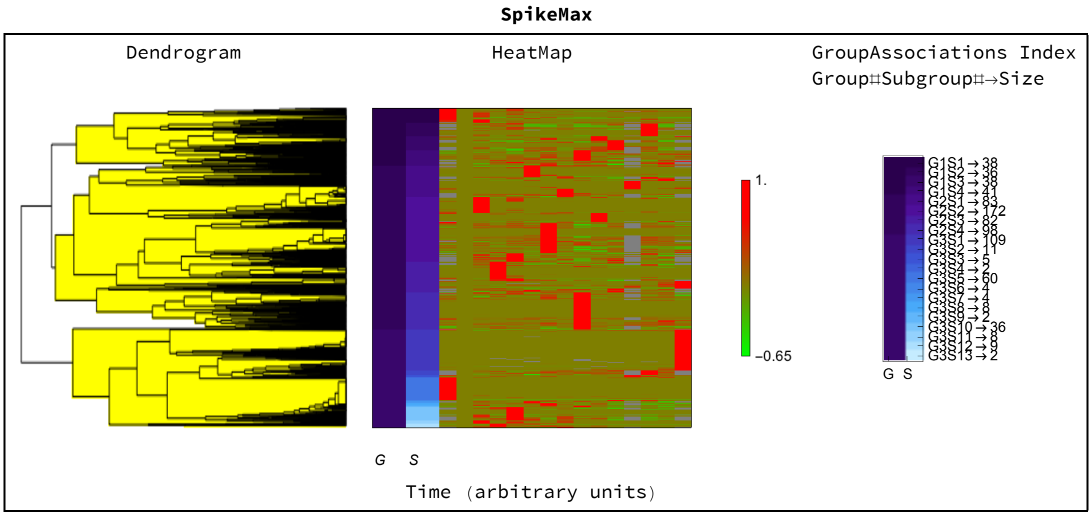

SpikeMin

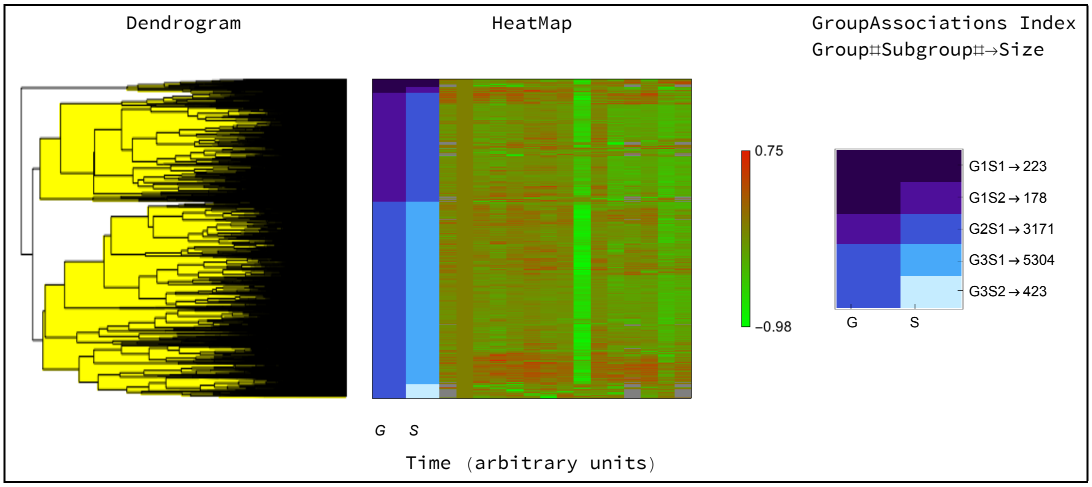

f1

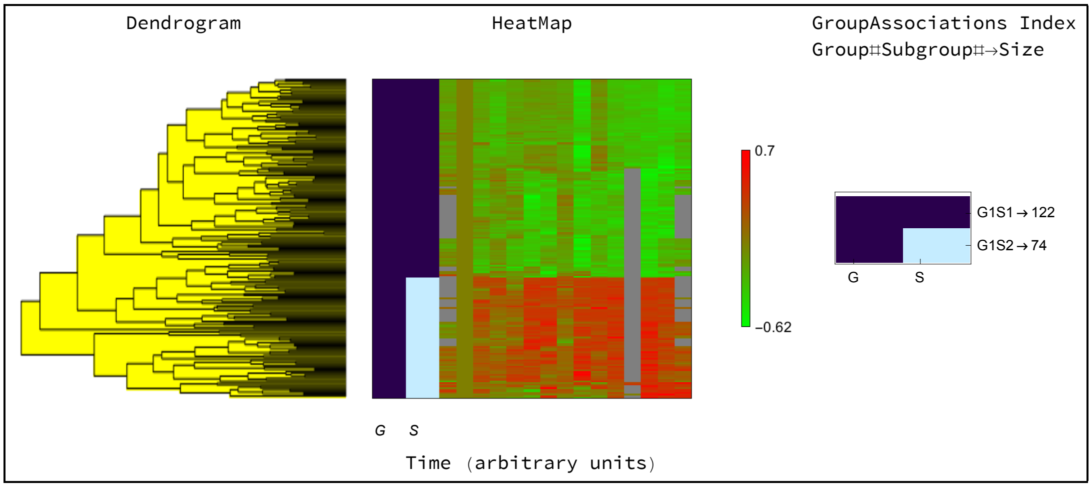

f2

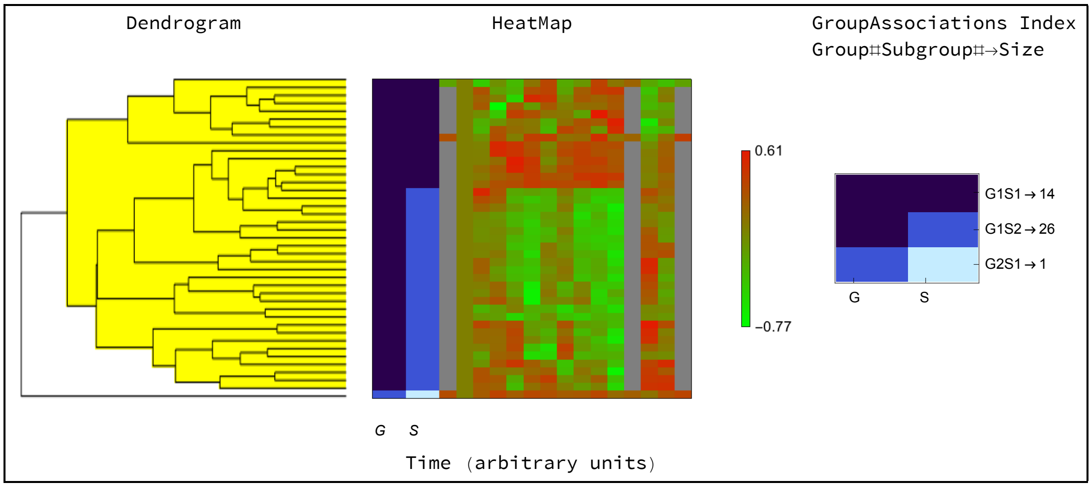

Out[125]=

f3

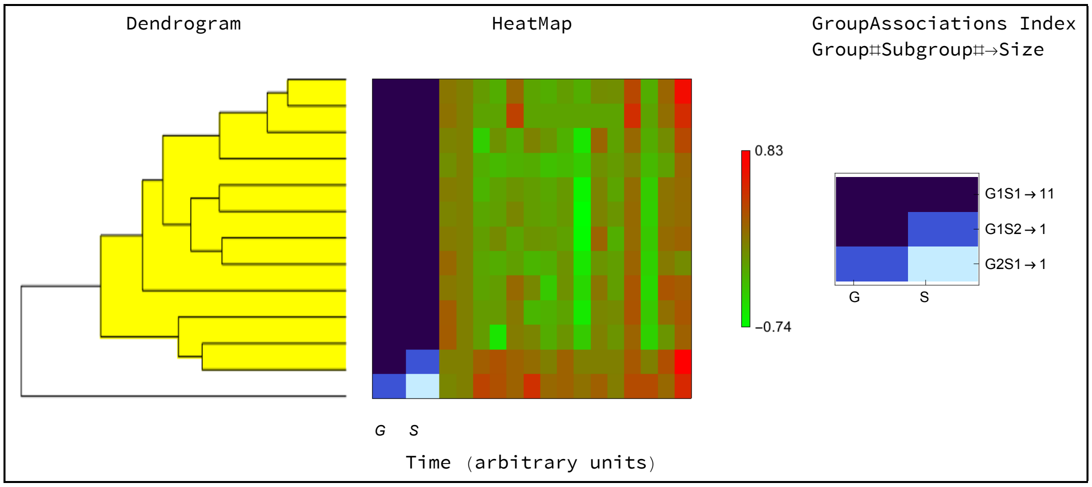

f4

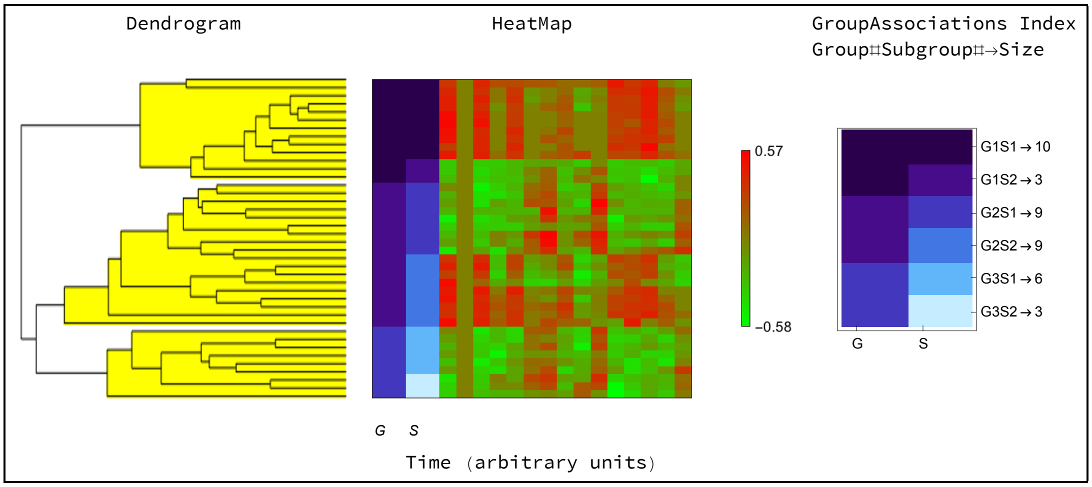

f5

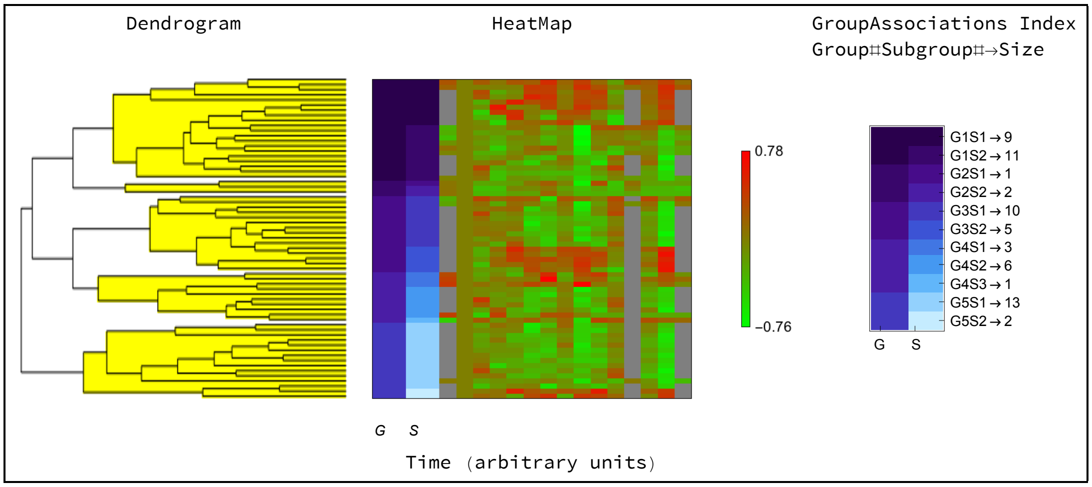

f6

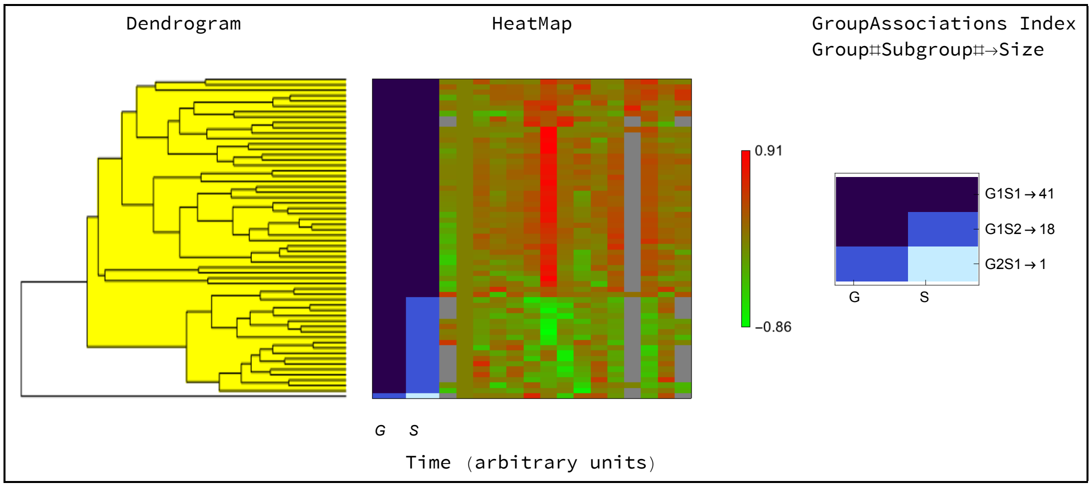

f7

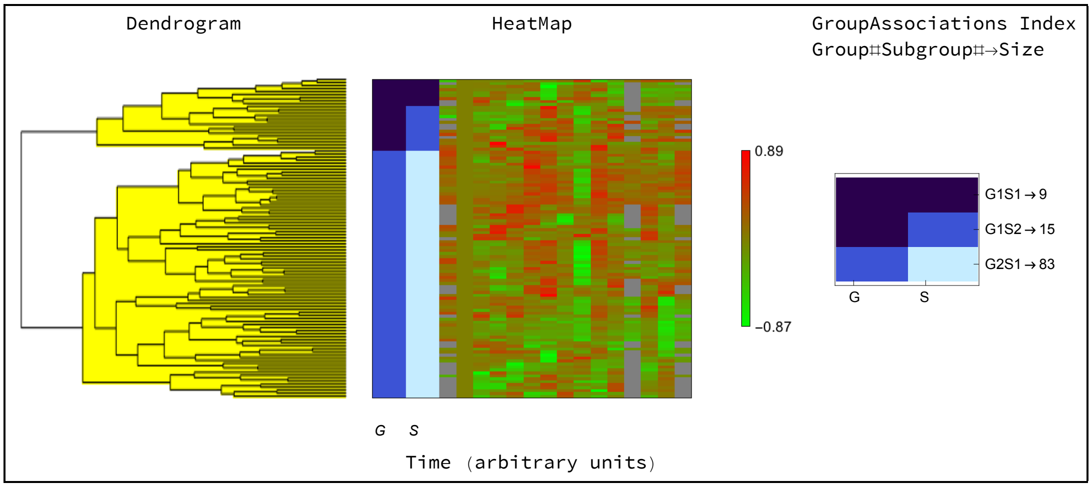

Annotation and Enrichment

Having carried out the classification and clustering of data base on its temporal pattern, we would like to perform annotation of these data for gene ontology (GO) and pathways from KEGG: Kyoto Encyclopedia of Genes and Genomes.

Gene Ontology Analysis

MathIOmica provides a `GOAnalysis` function using annotations (default is for human data) obtained from the Gene Ontology consortium, and by default uses human data annotated with UniProt IDs. The `GOAnalysis` function performs an over-representation (ORA) analysis, providing a "significance" cutoff based on a p-value assessed by a hypergeometric function.

GOAnalysis[*data*]	calculates input data over–representation analysis (ORA) for Gene Ontology (GO) categories. We note that the function utilizes ontologies obtained from the GO Consortium, and by default uses human data annotated with UniProt IDs.

Performing an over representation analysis for Gene Ontology (GO) terms, using clustered data in MathIOmica.

option name	default value	
AdditionalFilter	None	AdditionalFilter provides additional filtering that may be applied to the standard output structure to be returned.
AugmentDictionary	True	AugmentDictionary provides a choice whether or not to augment the current ConstantGeneDictionary variable or create a new one.
BackgroundSet	All	BackgroundSet provides a list of IDs (e.g. gene accessions) that should be considered as the background for the calculation.
FilterSignificant	True	FilterSignificant can be set to True to filter data based on whether the enrichment analysis is statistically significant, or if set to False to return all membership computations.
GeneDictionary	None	GeneDictionary points to an existing variable to use as a gene dictionary in annotations. If set to None the default ConstantGeneDictionary will be used.
GetGeneDictionaryOptions	{}	The GetGeneDictionaryOptions option specifies a list of options that will be passed to the internal GetGeneDictionary function.
GOAnalysisAssignerOptions	{}	The GOAnalysisAssignerOptions option specifies a list of options that will be passed to the internal GOAnalysisAssigner function.
HypothesisFunction	(Query["Results"][BenjaminiHochbergFDR[#1, SignificanceLevel -> #2]] &)	The HypothesisFunction option allows us to chose a function to implement multiple hypothesis testing. The default is using the BenjaminiHochbergFDR function.<br>The user can use any function f with three inputs, of the form f[#1,#2,#3] where the inputs refer to:<br>#1 is the p–value list,<br>#2 is a significance cutoff,<br>#3 is the number of GO associations overall being tested.<br>The function f must output a list of 3 values: {original p–value, adjusted p–value, True or False based on whether this value is considered statistically significant or not respectively}.
InputID	{"UniProt ID", "Gene Symbol"}	The InputID option specifies the kind of identifiers/accessions used as input.

MultipleList	False	MultipleList option specifies whether the input accessions list constituted a multi–omics list input that is annotated so. If this is the case, MultipleList is set to True and each input list ID should have the form {ID,"Omics Type Label"}, e.g. {"NFKB1","Protein"}, and the different omics type are treated as different for each ID. If MultipleList is set to False, and labeled IDs are provided, labels corresponding to the same ID are treated as equivalent to avoid overcounting.
MultipleListCorrection	None	MultipleListCorrection is an option whether or not to correct for multi–omics analysis. The choices are None, Automatic, or a custom number. This essen– tially enlarges the population by this factor to account for additional IDs being considered as the result of a multi–omics cluster analysis. If the value is set to Automatic the number of unique ID labels is used to make the correction.
OBOGODictionaryOptions	{}	OBOGODictionaryOptions specifies a list of options to be passed to the internal OBOGODictionary function that provides the GO annotations.
OBODictionaryVariable	None	OBODictionaryVariable can provide a GO annotation variable. If set to None, OBOGODictionary will be used internally to automatically generate the default GO annotation.
OntologyLengthFilter	2	OntologyLengthFilter can be used to set the value for which terms to consider in the computation, by excluding GO terms that have fewer items com– pared to the OntologyLengthFilter value. It is used by the internal GOAnalysisAssigner function.
OutputID	"UniProt ID"	The OutputID option takes a string value that specifies what kind of IDs/accessions to convert the input IDs to compute the GO enrichment.
pValueCutoff	0.05	pValueCutoff provides a cutoff p–value for adjusted p–values to assess statistical significance.
ReportFilter	1	ReportFilter provides a cutoff for membership in ontologies in selecting which terms/categories to return. It is used in conjunction with ReportFilterFunc- tion.
ReportFilterFunction	GreaterEqualThan	ReportFilterFunction specifies what operator form will be used to compare against ReportFilter option value in selecting which terms/categories to return. The default is to use GreaterEqualThan.
Species	"human"	The Species option specifies the species considered in the calculation.

TestFunction	N[1 - CDF[HypergeometricDistribution[#1, #2, #3], #4 - 1]] &	The TestFunction option provides a function used to calculate the p–values for the enrichment of each term. It can be a function of four inputs, f[#1,#2,#3,#4] (e.g. the default is using a hypergeometric distribution CDF, N[1–CDF[HypergeometricDistribution[#1,#2,#3],#4–1]]]. The four inputs refer to:<br>#1 is number of draws (members in group being tested),<br>#2 is number of successes for category in population,<br>#3 is total number of members in population,<br>#4 is number of successes (or more) in current group being tested for specific category.<br>The output is a p–value (real positive number ≤ 1).

Options for GOAnalysis.

The input data for GOAnalysis be a single list of n genes in the form:

$data = \{ID_1, ID_2, ..., ID_n\}$

The IDs may be provided as ID strings, or as labeled strings in the case of multiple omics being considered. Labeled IDs are provided as $\{\{ID_1, label_1\}, \{ID_2, label_2\}, ... \{ID_3, label_2\}\}$. The labels are typically a string, e.g. typically "RNA" or "Protein".

The default output contains each GO:term that was considered and found to be statistically significant. For each GO term we schematically have an association with keys GO : Term → {{testing outcomes}, {statistics}, {{GO term}, {Membership}}. The output has the following structures: for a single list input:

```
listOutput = <|
  GO : Term_1 → {{p - value_1, multiple hypothesis adjusted p - value_1, True / False for statistical significance},
    {{number of members in group being tested, number of successes for term_1 in population, total number of
       members in population, number of members (or more) in current group being tested associated to term_1},
     {{GO term_1 description, ontology category for term_1}, {input IDs associated to Term_1}}}},
  GO : Term_2 → {{p - value_2, multiple hypothesis adjusted p - value_2, True / False for statistical significance},
    {{number of members in group being tested, number of successes for term_2 in population, total number of
       members in population, number of members (or more) in current group being tested associated to term_2},
     {{GO term_2 description, ontology category for term_2}, {input IDs associated to Term_2}}}}, ...,
  GO : Term_n → {{p - value_n, multiple hypothesis adjusted p - value_n, True / False for statistical significance},
    {{number of members in group being tested, number of successes for term_n in population, total number of
       members in population, number of members (or more) in current group being tested associated to term_n},
     {{GO term_n description, ontology category for term_n}, {input IDs associated to term_n}}}}
 |>
```

GOAnalysis can also take as input the output of clustering of time series classification data, e.g. TimeSeriesClusters or TimeSeriesSingleClusters association of associations. The groups for each class will then have keys labeled "GroupAssociations", that include the labels used in the clustering. The labels must correspond to protein or gene accessions/IDs. For each class and group the corresponding GOAnalysis enrichment is computed and returned.

We also note that GOAnalysis provides a multiple-hypothesis adjusted p-value. By default, it utilizes a Benjamini-Hochberg false discovery rate (FDR) using BenjaminiHochbergFDR.

BenjaminiHochbergFDR[*pValues*]	calculates for a list of *pValues*, {p_1, p_2, ... p_N}, the Benjamini Hochberg approach false discovery rates (FDR).

Calculating a false discovery rate (FDR).

We carry out our GOAnalysis for all the classes and groups/subgroups. We only report terms for which there are at least 3 members, and additionally correct for multiple omics (2 sets of GO terms, one each for proteomics and transcriptomics). Please note that this is a time consuming computation.

```
In[126]:= goAnalysisCombined = GOAnalysis[combinedClusters, OntologyLengthFilter → 3,
   ReportFilter → 3, MultipleList → True, MultipleListCorrection → 2];
```

We see that the classification is maintained:

```
In[127]:= Keys@goAnalysisCombined
Out[127]= {SpikeMax, SpikeMin, f1, f2, f3, f4, f5, f6, f7}
```

Let us extract the top 3 results from all the "SpikeMax" data:

```
In[128]:= Query["SpikeMax", All, 1 ;; 3]@goAnalysisCombined
Out[128]= ⟨|G1S1 →
  ⟨|GO:0006351 → {{1.44719×10^-6, 0.000256153, True}, {25, 4570, 94 482, 9}, {{transcription, DNA-templated,
      biological_process}, {{{ZNF234, RNA}}, {{TP53INP2, RNA}}, {{O75175, Protein}}, {{ZNF841, RNA}},
      {{SCML1, RNA}}, {{ZNF514, RNA}}, {{ZNF169, RNA}}, {{ZSCAN30, RNA}}, {{ZNF436, RNA}}}}},
   GO:0003700 → {{0.0000156342, 0.00138363, True}, {25, 3246, 94 482, 7},
     {{transcription factor activity, sequence-specific DNA binding, molecular_function},
      {{{ZNF234, RNA}}, {{ZNF841, RNA}}, {{SCML1, RNA}}, {{ZNF514, RNA}},
       {{ZSCAN30, RNA}}, {{ZNF436, RNA}}, {{ZNF814, RNA}}}}},
   GO:0006355 → {{0.0000294529, 0.00173772, True}, {25, 6622, 94 482, 9},
     {{regulation of transcription, DNA-templated, biological_process},
      {{{ZNF234, RNA}}, {{O75175, Protein}}, {{ZNF841, RNA}}, {{SCML1, RNA}}, {{ZNF514, RNA}},
       {{ZNF169, RNA}}, {{ZSCAN30, RNA}}, {{ZNF436, RNA}}, {{ZNF814, RNA}}}}}|⟩,
 G1S2 → ⟨|GO:0005515 → {{0.000548629, 0.00537052, True}, {10, 17 602, 94 482, 7},
     {{protein binding, molecular_function}, {{{PLXNB3, RNA}}, {{PRKCDBP, RNA}},
       {{NUMBL, RNA}}, {{HIC1, RNA}}, {{HES1, RNA}}, {{UCN, RNA}}, {{C19orf44, RNA}}}}},
   GO:0005737 → {{0.00748941, 0.0146543, True}, {10, 13 296, 94 482, 5}, {{cytoplasm, cellular_component},
      {{{PRKCDBP, RNA}}, {{NUMBL, RNA}}, {{HIC1, RNA}}, {{HES1, RNA}}, {{HSD17B1, RNA}}}}},
   GO:0003677 → {{0.0112598, 0.0177077, True}, {10, 4688, 94 482, 3},
     {{DNA binding, molecular_function}, {{{ZDHHC1, RNA}}, {{HES1, RNA}}, {{TIGD3, RNA}}}}}|⟩,
 G1S3 → ⟨|GO:0046872 → {{0.00339359, 0.0356327, True}, {17, 6020, 94 482, 5},
     {{metal ion binding, molecular_function},
      {{{ZNF404, RNA}}, {{MOB3B, RNA}}, {{MMEL1, RNA}}, {{PHYHD1, RNA}}, {{LMTK3, RNA}}}}},
   GO:0005515 → {{0.00704381, 0.0376605, True}, {17, 17 602, 94 482, 8},
     {{protein binding, molecular_function}, {{{ZNF404, RNA}}, {{CEP70, RNA}}, {{MOB3B, RNA}},
       {{IL17RE, RNA}}, {{C19orf73, RNA}}, {{TMEM171, RNA}}, {{PHYHD1, RNA}}, {{PKN3, RNA}}}}}|⟩,
 G1S4 → ⟨|GO:0005743 → {{3.91396×10^-6, 0.000489245, True}, {25, 920, 94 482, 5},
     {{mitochondrial inner membrane, cellular_component}, {{{P10606, Protein}},
       {{P10809, Protein}}, {{Q9Y6N5, Protein}}, {{Q9H9B4, Protein}}, {{P51970, Protein}}}}},
   GO:0005739 → {{0.00015269, 0.0127242, True}, {25, 3200, 94 482, 6},
     {{mitochondrion, cellular_component}, {{{P10606, Protein}}, {{P10809, Protein}}, {{O95571, Protein}},
       {{Q9H9B4, Protein}}, {{P51970, Protein}}, {{Q96I99, Protein}}}}}, GO:0000139 →
    {{0.000613348, 0.0200226, True}, {25, 1500, 94 482, 4}, {{Golgi membrane, cellular_component},
      {{{Q8NF37, Protein}}, {{O75396, Protein}}, {{Q8WWP7, Protein}}, {{Q13439, Protein}}}}}|⟩,
 G2S1 → ⟨|GO:0005829 → {{0.00420485, 0.0395925, True}, {44, 6952, 94 482, 9},
     {{cytosol, cellular_component}, {{{STAP2, RNA}}, {{SNX16, RNA}}, {{SH3BGR, RNA}}, {{TCAP, RNA}},
       {{RILPL1, RNA}}, {{ACSBG1, RNA}}, {{HSD17B14, RNA}}, {{MYL4, RNA}}, {{LRRC16A, RNA}}}}}, GO:0005515 →
    {{0.00425849, 0.0395925, True}, {44, 17 602, 94 482, 16}, {{protein binding, molecular_function},
      {{{BIK, RNA}}, {{SLC25A4, RNA}}, {{LRRC20, RNA}}, {{CIB2, RNA}}, {{TLDC1, RNA}}, {{STAP2, RNA}},
```

[illegible]
{{ARMCX1, RNA}}, {{MEOX1, RNA}}, {{OLFM1, RNA}}, {{ARHGEF39, RNA}}, {{RGS16, RNA}},
{{TCAP, RNA}}, {{HSD17B14, RNA}}, {{EPB42, RNA}}, {{LRRC16A, RNA}}, {{PRX, RNA}}}}}, GO:0005886 →
{{0.0100487, 0.0432511, True}, {44, 9422, 94 482, 10}, {{plasma membrane, cellular_component},
{{{STAP2, RNA}}, {{ARHGEF39, RNA}}, {{RGS16, RNA}}, {{RILPL1, RNA}}, {{RAB40B, RNA}},
{{MCOLN3, RNA}}, {{EPB42, RNA}}, {{LRRC16A, RNA}}, {{PRX, RNA}}, {{SLC14A1, RNA}}}}}} |〉,
G2S2 → 〈| GO:0005515 → {{8.06733 $\times 10^{-19}$, 7.55102 $\times 10^{-16}$, True}, {112, 17 602, 94 482, 63},
{{protein binding, molecular_function}, {{{TONSL, RNA}}, {{C17orf67, RNA}}, {{PKD2, RNA}},
{{TRIM74, RNA}}, {{KCNH2, RNA}}, {{TXNDC16, RNA}}, {{PBLD, RNA}}, {{TMEM30B, RNA}}, {{BMPR1A, RNA}},
{{SPSB1, RNA}}, {{GSTCD, RNA}}, {{ZNF2, RNA}}, {{P61457, Protein}}, {{Q9HC16, Protein}},
{{SPRY1, RNA}}, {{P54136, Protein}}, {{Q13596, Protein}}, {{P25098, Protein}}, {{P41227, Protein}},
{{Q13043, Protein}}, {{O14732, Protein}}, {{Q8N8A2, Protein}}, {{Q7Z4H3, Protein}},
{{O60826, Protein}}, {{Q9UBE0, Protein}}, {{ZNF772, RNA}}, {{PHOSPHO2, RNA}}, {{LIN9, RNA}},
{{POP1, RNA}}, {{O94979, Protein}}, {{Q9Y3D0, Protein}}, {{P35998, Protein}}, {{P25788, Protein}},
{{Q13347, Protein}}, {{Q9Y2V2, Protein}}, {{Q5JSL3, Protein}}, {{Q92888, Protein}},
{{O75534, Protein}}, {{O60841, Protein}}, {{O43813, Protein}}, {{DNAJB4, RNA}}, {{Q13148, Protein}},
{{Q2TAY7, Protein}}, {{O94776, Protein}}, {{P52756, Protein}}, {{P06127, Protein}},
{{P19474, Protein}}, {{Q02818, Protein}}, {{P07766, Protein}}, {{Q9Y333, Protein}},
{{P13861, Protein}}, {{Q9Y285, Protein}}, {{P60900, Protein}}, {{P13612, Protein}},
{{Q7L2H7, Protein}}, {{Q07812, Protein}}, {{O14745, Protein}}, {{Q86UP2, Protein}},
{{Q8N1G4, Protein}}, {{Q9UEU0, Protein}}, {{Q01082, Protein}}, {{TNFRSF6B, RNA}}, {{ZNF543, RNA}}}}},
GO:0005829 → {{1.51933 $\times 10^{-14}$, 7.11047 $\times 10^{-12}$, True}, {112, 6952, 94 482, 36},
{{cytosol, cellular_component}, {{{NT5DC3, RNA}}, {{PKD2, RNA}}, {{PGAM2, RNA}}, {{SPSB1, RNA}},
{{P61457, Protein}}, {{Q9HC16, Protein}}, {{SPRY1, RNA}}, {{P55263, Protein}},
{{P54136, Protein}}, {{Q13596, Protein}}, {{P25098, Protein}}, {{Q13043, Protein}},
{{O14732, Protein}}, {{Q9UBE0, Protein}}, {{KLHL14, RNA}}, {{O94979, Protein}},
{{P35998, Protein}}, {{P25788, Protein}}, {{Q13347, Protein}}, {{Q9Y2V2, Protein}},
{{Q5JSL3, Protein}}, {{P63220, Protein}}, {{Q92888, Protein}}, {{O60841, Protein}},
{{DNAJB4, RNA}}, {{P19474, Protein}}, {{Q9Y333, Protein}}, {{P13861, Protein}},
{{Q9Y285, Protein}}, {{O43252, Protein}}, {{P60900, Protein}}, {{Q7L2H7, Protein}},
{{P56192, Protein}}, {{Q07812, Protein}}, {{Q9UEU0, Protein}}, {{Q01082, Protein}}}}}},
GO:0005737 → {{1.04762 $\times 10^{-11}$, 3.26857 $\times 10^{-9}$, True}, {112, 13 296, 94 482, 45},
{{cytoplasm, cellular_component}, {{{TONSL, RNA}}, {{PKD2, RNA}}, {{SLC46A1, RNA}}, {{STPG1, RNA}},
{{PARM1, RNA}}, {{RDH10, RNA}}, {{PBLD, RNA}}, {{C9orf3, RNA}}, {{TMSB15B, RNA}}, {{SAPCD2, RNA}},
{{GSTCD, RNA}}, {{P61457, Protein}}, {{Q9HC16, Protein}}, {{SPRY1, RNA}}, {{P55263, Protein}},
{{P54136, Protein}}, {{Q13596, Protein}}, {{P25098, Protein}}, {{P41227, Protein}},
{{Q13043, Protein}}, {{O14732, Protein}}, {{C11orf82, RNA}}, {{RALGAPA1, RNA}}, {{O94979, Protein}},
{{Q9Y3D0, Protein}}, {{P35998, Protein}}, {{P25788, Protein}}, {{P63220, Protein}},
{{Q92888, Protein}}, {{O75534, Protein}}, {{O60841, Protein}}, {{Q32P44, Protein}},
{{O43813, Protein}}, {{DNAJB4, RNA}}, {{Q13148, Protein}}, {{Q2TAY7, Protein}}, {{P19474, Protein}},
{{P13861, Protein}}, {{Q9Y285, Protein}}, {{P60900, Protein}}, {{P56192, Protein}},
{{O14745, Protein}}, {{Q9UEU0, Protein}}, {{Q01082, Protein}}, {{O43402, Protein}}}}}} |〉,
G2S3 → 〈| GO:0005515 → {{3.79873 $\times 10^{-6}$, 0.00213109, True}, {48, 17 602, 94 482, 23},
{{protein binding, molecular_function},
{{{NTNG2, RNA}}, {{LDHD, RNA}}, {{IFIT3, RNA}}, {{BCL2A1, RNA}}, {{SAMD4A, RNA}}, {{TGM2, RNA}},
{{KCNJ15, RNA}}, {{APOL4, RNA}}, {{PRR16, RNA}}, {{ETV7, RNA}}, {{SLC6A12, RNA}}, {{BATF2, RNA}},
{{OSGIN1, RNA}}, {{TMEM51, RNA}}, {{C1QB, RNA}}, {{WASF1, RNA}}, {{APOE, RNA}}, {{TUBB2B, RNA}},
{{IGFBP2, RNA}}, {{KRT18, RNA}}, {{PTK7, RNA}}, {{C8orf44-SGK3, RNA}}, {{NBL1, RNA}}}}}},
GO:0043065 → {{0.0000277955, 0.00779663, True}, {48, 694, 94 482, 5},
{{positive regulation of apoptotic process, biological_process},
{{{BCL2A1, RNA}}, {{TGM2, RNA}}, {{PLA2G4A, RNA}}, {{OSGIN1, RNA}}, {{SFRP2, RNA}}}}}},
GO:0051384 → {{0.0000869136, 0.0097517, True}, {48, 166, 94 482, 3},
{{response to glucocorticoid, biological_process},
{{{PLA2G4A, RNA}}, {{MDK, RNA}}, {{IGFBP2, RNA}}}}}} |〉,
G2S4 → 〈| GO:0005739 → {{1.25378 $\times 10^{-16}$, 4.65152 $\times 10^{-14}$, True}, {58, 3200, 94 482, 21},
{{mitochondrion, cellular_component},
{{{P22695, Protein}}, {{P83111, Protein}}, {{Q8N4H5, Protein}}, {{Q99798, Protein}},
{{P38646, Protein}}, {{O75323, Protein}}, {{P06576, Protein}}, {{P55084, Protein}},
{{P49411, Protein}}, {{Q9NUJ1, Protein}}, {{AS3MT, RNA}}, {{Q9NSE4, Protein}}, {{P10515, Protein}},
{{O96008, Protein}}, {{P42126, Protein}}, {{P22033, Protein}}, {{P13804, Protein}},
{{Q16822, Protein}}, {{P40939, Protein}}, {{Q02218, Protein}}, {{P22307, Protein}}}}}},
GO:0005759 → {{1.45524 $\times 10^{-11}$, 2.69947 $\times 10^{-9}$, True}, {58, 686, 94 482, 10},
{{mitochondrial matrix, cellular_component}, {{{Q99798, Protein}}, {{P06576, Protein}},

[illegible]
{{Q9NUJ1, Protein}}, {{Q9NSE4, Protein}}, {{P10515, Protein}}, {{P42126, Protein}},
{{P22033, Protein}}, {{P13804, Protein}}, {{Q16822, Protein}}, {{Q02218, Protein}}}}},
GO:0042645 → {{2.75241×10^{-9}, 3.40381×10^{-7}, True}, {58, 88, 94482, 5},
{{mitochondrial nucleoid, cellular_component}, {{{P38646, Protein}},
{{P06576, Protein}}, {{P55084, Protein}}, {{P49411, Protein}}, {{P40939, Protein}}}}}|⟩,
G3S1 → ⟨|GO:0005515 → {{1.79745×10^{-20}, 1.91069×10^{-17}, True}, {93, 17602, 94482, 58},
{{protein binding, molecular_function}, {{{ALPL, RNA}}, {{PMEL, RNA}}, {{PAWR, RNA}}, {{FAM64A, RNA}},
{{CDH2, RNA}}, {{TEAD2, RNA}}, {{CBS, RNA}}, {{CDC20, RNA}}, {{CRABP2, RNA}}, {{CDK1, RNA}},
{{CDCA5, RNA}}, {{FOXM1, RNA}}, {{MAP1B, RNA}}, {{USP44, RNA}}, {{PODXL, RNA}}, {{SLC7A8, RNA}},
{{NUSAP1, RNA}}, {{CCNB2, RNA}}, {{TNFRSF12A, RNA}}, {{PLK1, RNA}}, {{NQO1, RNA}}, {{GINS2, RNA}},
{{TUBB3, RNA}}, {{SEMA6A, RNA}}, {{AURKB, RNA}}, {{MYH10, RNA}}, {{TOP2A, RNA}}, {{BIRC5, RNA}},
{{PYCR1, RNA}}, {{GPC3, RNA}}, {{RBPMS, RNA}}, {{DMKN, RNA}}, {{HMGB3, RNA}}, {{RRM2, RNA}},
{{FZD7, RNA}}, {{GFPT2, RNA}}, {{TPX2, RNA}}, {{DNAJB5, RNA}}, {{HMGA2, RNA}}, {{KRT8, RNA}},
{{MYCN, RNA}}, {{FBLN1, RNA}}, {{THY1, RNA}}, {{CRMP1, RNA}}, {{CD276, RNA}}, {{DNMT3B, RNA}},
{{ELOVL6, RNA}}, {{KIAA0101, RNA}}, {{SPATC1L, RNA}}, {{CCNB1, RNA}}, {{FGFR1, RNA}}, {{FANCL, RNA}},
{{DPPA4, RNA}}, {{PCGF2, RNA}}, {{UCHL1, RNA}}, {{IGF2BP3, RNA}}, {{UBE2C, RNA}}, {{BEX1, RNA}}}}},
GO:0007067 → {{7.67752×10^{-12}, 4.0806×10^{-9}, True}, {93, 540, 94482, 11},
{{mitotic nuclear division, biological_process},
{{{FAM64A, RNA}}, {{CDC20, RNA}}, {{CDK1, RNA}}, {{CDCA5, RNA}}, {{USP44, RNA}}, {{CCNB2, RNA}},
{{PLK1, RNA}}, {{TUBB3, RNA}}, {{BIRC5, RNA}}, {{TPX2, RNA}}, {{HMGA2, RNA}}}}},
GO:0005829 → {{6.98517×10^{-11}, 2.47508×10^{-8}, True}, {93, 6952, 94482, 28},
{{cytosol, cellular_component}, {{{PTMS, RNA}}, {{NXN, RNA}}, {{CBS, RNA}}, {{CDC20, RNA}},
{{CRABP2, RNA}}, {{CDK1, RNA}}, {{CDCA5, RNA}}, {{MAP1B, RNA}}, {{BCAT1, RNA}}, {{POLR3G, RNA}},
{{CCNB2, RNA}}, {{PLK1, RNA}}, {{NQO1, RNA}}, {{AURKB, RNA}}, {{MYH10, RNA}}, {{BIRC5, RNA}},
{{RRM2, RNA}}, {{GFPT2, RNA}}, {{TPX2, RNA}}, {{DNAJB5, RNA}}, {{THY1, RNA}}, {{CRMP1, RNA}},
{{CCNB1, RNA}}, {{FGFR1, RNA}}, {{QPRT, RNA}}, {{UCHL1, RNA}}, {{IGF2BP3, RNA}}, {{UBE2C, RNA}}}}}|⟩,
G3S2 → ⟨||⟩, G3S3 → ⟨|GO:0005886 → {{0.00849073, 0.0280194, True}, {5, 9422, 94482, 3},
{{plasma membrane, cellular_component}, {{{BAMBI, RNA}}, {{TMEM88, RNA}}, {{EFNA3, RNA}}}}}|⟩,
G3S4 → ⟨||⟩, G3S5 → ⟨|GO:0006351 → {{0.00012294, 0.0257683, True}, {41, 4570, 94482, 9},
{{transcription, DNA-templated, biological_process},
{{{HOXC4, RNA}}, {{ZNF532, RNA}}, {{ZNF823, RNA}}, {{ZNF441, RNA}}, {{ZNF440, RNA}},
{{ZBTB26, RNA}}, {{ZSCAN22, RNA}}, {{ZNF577, RNA}}, {{TBX19, RNA}}}}},
GO:0046872 → {{0.000196704, 0.0257683, True}, {41, 6020, 94482, 10},
{{metal ion binding, molecular_function},
{{{B3GAT1, RNA}}, {{ZNF532, RNA}}, {{ZNF823, RNA}}, {{ZNF441, RNA}}, {{ZNF440, RNA}},
{{ADHFE1, RNA}}, {{ZBTB26, RNA}}, {{ZSCAN22, RNA}}, {{ZNF577, RNA}}, {{ENPP5, RNA}}}}},
GO:0005515 → {{0.000596511, 0.0391301, True}, {41, 17602, 94482, 17},
{{protein binding, molecular_function},
{{{E2F2, RNA}}, {{HOXC4, RNA}}, {{CEP152, RNA}}, {{ZNF440, RNA}}, {{NLRP2, RNA}}, {{STK36, RNA}},
{{C1QTNF6, RNA}}, {{SLC22A5, RNA}}, {{KLHL3, RNA}}, {{ZBTB26, RNA}}, {{OCRL, RNA}}, {{DLG5, RNA}},
{{ZSCAN22, RNA}}, {{AGPAT4, RNA}}, {{DAPK2, RNA}}, {{ITIH4, RNA}}, {{PACSIN1, RNA}}}}}|⟩,
G3S6 → ⟨||⟩, G3S7 → ⟨||⟩, G3S8 → ⟨|GO:0005515 → {{0.0000521081, 0.00505449, True}, {8, 17602, 94482, 7},
{{protein binding, molecular_function}, {{{O14933, Protein}}, {{Q9Y6Y8, Protein}},
{{Q15819, Protein}}, {{P19784, Protein}}, {{P01732, Protein}}, {{RFX3, RNA}}, {{O95218, Protein}}}}},
GO:0005654 → {{0.00213687, 0.0188433, True}, {8, 7498, 94482, 4}, {{nucleoplasm, cellular_component},
{{{O14933, Protein}}, {{Q15819, Protein}}, {{P19784, Protein}}, {{O95218, Protein}}}}},
GO:0006355 → {{0.0147442, 0.0332601, True}, {8, 6622, 94482, 3},
{{regulation of transcription, DNA-templated, biological_process},
{{{P19784, Protein}}, {{RFX3, RNA}}, {{O95218, Protein}}}}}|⟩,
G3S9 → ⟨||⟩, G3S10 → ⟨|GO:0005737 → {{0.000313059, 0.0255235, True}, {18, 13296, 94482, 9},
{{cytoplasm, cellular_component}, {{{RIMKLB, RNA}}, {{TSSK6, RNA}}, {{SEC14L2, RNA}},
{{ZKSCAN3, RNA}}, {{IFT140, RNA}}, {{SMTN, RNA}}, {{NOS3, RNA}}, {{AIFM2, RNA}}, {{RFX2, RNA}}}}},
GO:0070062 → {{0.00319044, 0.0257389, True}, {18, 5572, 94482, 5},
{{extracellular exosome, cellular_component},
{{{GPRC5C, RNA}}, {{C1QC, RNA}}, {{SEC14L2, RNA}}, {{HIST4H4, RNA}}, {{LAMB2, RNA}}}}},
GO:0005576 → {{0.00548073, 0.0258897, True}, {18, 3882, 94482, 4},
{{extracellular region, cellular_component},
{{{C1QC, RNA}}, {{HIST4H4, RNA}}, {{TFPI, RNA}}, {{LAMB2, RNA}}}}}|⟩,
G3S11 → ⟨||⟩, G3S12 → ⟨|GO:0005829 → {{0.00672211, 0.0131387, True}, {6, 6952, 94482, 3},
{{cytosol, cellular_component}, {{{HTRA1, RNA}}, {{MSR1, RNA}}, {{SPR, RNA}}}}},
GO:0005886 → {{0.0157255, 0.0208061, True}, {6, 9422, 94482, 3}, {{plasma membrane, cellular_component},
{{{HTRA1, RNA}}, {{MMP14, RNA}}, {{MSR1, RNA}}}}}|⟩, G3S13 → ⟨||⟩|⟩

Let us extract the names of the top 10 ontology group results from all the "f1" Group1 subgroup 1 data (G1S1). These are in the 3rd list, first component for GOAnalysis outputs (see above and documentation:

In[129]:= **Query["f1", "G1S1", All, 3, 1]@goAnalysisCombined**

```
Out[129]= <|GO:0005515 → {protein binding, molecular_function},
 GO:0070062 → {extracellular exosome, cellular_component},
 GO:0016020 → {membrane, cellular_component}, GO:0005783 → {endoplasmic reticulum, cellular_component},
 GO:0007049 → {cell cycle, biological_process}, GO:0005737 → {cytoplasm, cellular_component},
 GO:0036498 → {IRE1-mediated unfolded protein response, biological_process},
 GO:0048208 → {COPII vesicle coating, biological_process},
 GO:0035257 → {nuclear hormone receptor binding, molecular_function},
 GO:0005741 → {mitochondrial outer membrane, cellular_component},
 GO:0009986 → {cell surface, cellular_component}, GO:0042493 → {response to drug, biological_process},
 GO:0005829 → {cytosol, cellular_component}, GO:0005634 → {nucleus, cellular_component},
 GO:0044255 → {cellular lipid metabolic process, biological_process},
 GO:0050714 → {positive regulation of protein secretion, biological_process},
 GO:0031982 → {vesicle, cellular_component}, GO:0030331 → {estrogen receptor binding, molecular_function},
 GO:1901215 → {negative regulation of neuron death, biological_process},
 GO:0000139 → {Golgi membrane, cellular_component},
 GO:0030521 → {androgen receptor signaling pathway, biological_process},
 GO:0005080 → {protein kinase C binding, molecular_function},
 GO:0007155 → {cell adhesion, biological_process},
 GO:0005791 → {rough endoplasmic reticulum, cellular_component},
 GO:0004402 → {histone acetyltransferase activity, molecular_function},
 GO:0003713 → {transcription coactivator activity, molecular_function},
 GO:0051592 → {response to calcium ion, biological_process},
 GO:0005886 → {plasma membrane, cellular_component},
 GO:0043022 → {ribosome binding, molecular_function}, GO:0005654 → {nucleoplasm, cellular_component},
 GO:0030335 → {positive regulation of cell migration, biological_process},
 GO:0006888 → {ER to Golgi vesicle-mediated transport, biological_process},
 GO:0005788 → {endoplasmic reticulum lumen, cellular_component},
 GO:0045893 → {positive regulation of transcription, DNA-templated, biological_process},
 GO:0051087 → {chaperone binding, molecular_function},
 GO:0042470 → {melanosome, cellular_component}, GO:0019886 →
  {antigen processing and presentation of exogenous peptide antigen via MHC class II, biological_process},
 GO:0005925 → {focal adhesion, cellular_component}, GO:0030496 → {midbody, cellular_component},
 GO:0005789 → {endoplasmic reticulum membrane, cellular_component},
 GO:0043066 → {negative regulation of apoptotic process, biological_process},
 GO:0045944 → {positive regulation of transcription from RNA polymerase II promoter, biological_process},
 GO:0007229 → {integrin-mediated signaling pathway, biological_process},
 GO:0000122 → {negative regulation of transcription from RNA polymerase II promoter, biological_process},
 GO:0005802 → {trans-Golgi network, cellular_component},
 GO:0046872 → {metal ion binding, molecular_function},
 GO:0005911 → {cell-cell junction, cellular_component}, GO:0007568 → {aging, biological_process},
 GO:0008017 → {microtubule binding, molecular_function},
 GO:0007420 → {brain development, biological_process},
 GO:0005102 → {receptor binding, molecular_function}, GO:0007411 → {axon guidance, biological_process},
 GO:0045087 → {innate immune response, biological_process},
 GO:0005516 → {calmodulin binding, molecular_function}, GO:0005769 → {early endosome, cellular_component},
 GO:0006351 → {transcription, DNA-templated, biological_process},
 GO:0005739 → {mitochondrion, cellular_component}, GO:0004872 → {receptor activity, molecular_function},
 GO:0019904 → {protein domain specific binding, molecular_function},
 GO:0003674 → {molecular_function, molecular_function},
 GO:0006457 → {protein folding, biological_process}, GO:0005794 → {Golgi apparatus, cellular_component}|>
```

Let us extract the corresponding p-values/test results of the top 10 ontology group results from all the "SpikeMin" Group1 subgroup 1 data (G1S1). These are in the 1st list for `GOAnalysis` outputs (see above and documentation:

In[130]:=
```
Query["f1", "G1S1", All, 1]@goAnalysisCombined
```

Out[130]=
```
⟨|GO:0005515 → {4.60247×10^-22, 4.36774×10^-19, True}, GO:0070062 → {3.1437×10^-13, 1.49169×10^-10, True},
 GO:0016020 → {7.30541×10^-13, 2.31094×10^-10, True}, GO:0005783 → {3.74009×10^-7, 0.0000887337, True},
 GO:0007049 → {5.56461×10^-7, 0.000105616, True}, GO:0005737 → {1.03344×10^-6, 0.000163455, True},
 GO:0036498 → {3.68563×10^-6, 0.000499666, True}, GO:0048208 → {8.42947×10^-6, 0.000999946, True},
 GO:0035257 → {0.0000122247, 0.00128903, True}, GO:0005741 → {0.0000174722, 0.00154095, True},
 GO:0009986 → {0.0000178613, 0.00154095, True}, GO:0042493 → {0.0000316072, 0.00249961, True},
 GO:0005829 → {0.0000462412, 0.00272312, True}, GO:0005634 → {0.0000466061, 0.00272312, True},
 GO:0044255 → {0.0000487809, 0.00272312, True}, GO:0050714 → {0.0000487809, 0.00272312, True},
 GO:0031982 → {0.0000522955, 0.00275714, True}, GO:0030331 → {0.0000568608, 0.00284005, True},
 GO:1901215 → {0.0000612096, 0.00290439, True}, GO:0000139 → {0.0000703303, 0.00317826, True},
 GO:0030521 → {0.000086228, 0.00371956, True}, GO:0005080 → {0.000110447, 0.00455716, True},
 GO:0007155 → {0.000125175, 0.00494961, True}, GO:0005791 → {0.000131254, 0.00498239, True},
 GO:0004402 → {0.000162743, 0.00594013, True}, GO:0003713 → {0.000171329, 0.00602189, True},
 GO:0051592 → {0.000198769, 0.00628771, True}, GO:0005886 → {0.000252997, 0.00732351, True},
 GO:0043022 → {0.000350386, 0.00950045, True}, GO:0005654 → {0.000380395, 0.0100276, True},
 GO:0030335 → {0.000413397, 0.0106031, True}, GO:0006888 → {0.000447197, 0.0110947, True},
 GO:0005788 → {0.000455946, 0.0110947, True}, GO:0045893 → {0.000489656, 0.0114245, True},
 GO:0051087 → {0.000660253, 0.0133315, True}, GO:0042470 → {0.000864631, 0.0170945, True},
 GO:0019886 → {0.000940696, 0.0177944, True}, GO:0005925 → {0.00119546, 0.0203355, True},
 GO:0030496 → {0.00182027, 0.0278619, True}, GO:0005789 → {0.00185159, 0.0278915, True},
 GO:0043066 → {0.00229835, 0.031664, True}, GO:0045944 → {0.00230223, 0.031664, True},
 GO:0007229 → {0.00242647, 0.0324327, True}, GO:0000122 → {0.00263589, 0.0342666, True},
 GO:0005802 → {0.00303474, 0.0378403, True}, GO:0046872 → {0.00307029, 0.0378403, True},
 GO:0005911 → {0.00398012, 0.0407733, True}, GO:0007568 → {0.00437576, 0.0407733, True},
 GO:0008017 → {0.00491032, 0.0407733, True}, GO:0007420 → {0.00653278, 0.0407733, True},
 GO:0005102 → {0.00740983, 0.0407733, True}, GO:0007411 → {0.00752518, 0.0407733, True},
 GO:0045087 → {0.00771692, 0.0407733, True}, GO:0005516 → {0.00829993, 0.0407733, True},
 GO:0005769 → {0.00839995, 0.0407733, True}, GO:0006351 → {0.00860491, 0.0407733, True},
 GO:0005739 → {0.00878947, 0.0407733, True}, GO:0004872 → {0.0117346, 0.0451985, True},
 GO:0019904 → {0.0118571, 0.0451985, True}, GO:0003674 → {0.0119248, 0.0451985, True},
 GO:0006457 → {0.0164626, 0.0495234, True}, GO:0005794 → {0.0164904, 0.0495234, True}|⟩
```

Pathway Analysis

Enrichment of Genomic KEGG Pathways (KEGG: Kyoto Encyclopedia of Genes and Genomes)

MathIOmica provides a `KEGGAnalysis` function using annotations (default is for human data) obtained from KEGG: Kyoto Encyclopedia of Genes and Genomes, and by default uses human data annotated with KEGG Gene IDs. The `KEGGAnalysis` function performs an over-representation (ORA) analysis, providing a "significance" cutoff based on a p-value assessed by a hypergeometric function.

`KEGGAnalysis[`*data*`]`	calculates input data over–representation analysis for KEGG: Kyoto Encyclopedia of Genes and Genomes pathways. We note that the function utilizes data obtained from the KEGG databases, and by default uses human data annotated by "KEGG Gene ID".

Performing an over representation analysis for KEGG:Kyoto Encyclopedia of Genes and Genenomes pathways, using clustered data in MathIOmica.

option name	default value

AdditionalFilter	None	AdditionalFilter provides additional filtering that may be applied to the standard output structure to be returned.
AnalysisType	"Genomic"	AnalysisType provides a selection for the type of analysis to perform. "Genomic" analysis (default) uses gene identifier based analysis. "Molecular" analysis uses molecular analysis. Setting the option to All carries out all possible analysis types for the input data.
AugmentDictionary	True	AugmentDictionary provides a choice whether or not to augment the current ConstantGeneDictionary variable or create a new one.
BacgroundSet	All	BackgroundSet provides a list of IDs (e.g. gene accessions) that should be considered as the background for the calculation.
FilterSignificant	True	FilterSignificant can be set to True to filter data based on whether the enrichment analysis is statistically significant, or if set to False to return all membership computations.
GeneDictionary	None	GeneDictionary points to an existing variable to use as a gene dictionary in annotations. If set to None the default ConstantGeneDictionary will be used.
GetGeneDictionaryOptions	{}	The GetGeneDictionaryOptions option specifies a list of options that will be passed to the internal GetGeneDictionary function.
HypothesisFunction	(Query["Results"][BenjaminiHochbergFDR[#1, SignificanceLevel -> #2]] &) &	The HypothesisFunction option allows us to chose a function to implement multiple hypothesis testing. The default is using the BenjaminiHochbergFDR function.<br>The user can use any function f with three inputs, of the form f[#1,#2,#3] where the inputs refer to:<br>#1 is the p−value list,<br>#2 is a significance cutoff,<br>#3 is the number of GO associations overall being tested.<br>The function f must output a list of 3 values: {original p−value, adjusted p−value, True or False based on whether this value is considered statistically significant or not respectively}.
InputID	{"UniProt ID", "Gene Symbol"}	The InputID option specifies the kind of identifiers/accessions used as input.
KEGGAnalysisAssignerOptions	{}	The KEGGAnalysisAssignerOptions option specifies a list of options that will be passed to the internal KEGGAnalysisAssigner function.
KEGGDatabase	"pathway"	KEGGDatabase value indicates which KEGG database to use as the target database.
KEGGDictionaryOptions	{}	KEGGDictionaryOptions specifies a list of options to be passed to the internal KEGGDictionary function that provides the KEGG annotations.
KEGGDictionaryVariable	None	KEGGDictionaryVariable can provide a KEGG annotation variable. If set to None, KEGGDictionary will be used internally to automatically generate the default KEGG annotation.

KEGGMolecular	"cpd"	KEGGMolecular specifies which database to use for molecular analysis. The default is the compound database ("cpd").
KEGGOrganism	"hsa"	KEGGOrganism indicates which organism (org) to use for "Genomic" type of analysis. The default is human analysis org="hsa".
MathIOmicaDataDirectory	ConstantMathIOmica-DataDirectory	MathIOmicaDataDirectory option specifies the directory where the default MathIOmica package data are stored. By default the option is set to create the standard directory if it does not exist already.
MolecularInputID	{"cpd"}	MolecularInputID is a string list to indicate the kind of ID to use for the input molecule entries.
MolecularOutputID	"cpd"	MolecularOutputID is a string to indicate the kind of ID to convert input molecule entries. The default is "cpd" consistently with use of the "cpd" database as the default molecular analysis.
MolecularSpecies	"compound"	MolecularSpecies specifies the kind of molecular input.
MultipleList	False	MultipleList option specifies whether the input accessions list constituted a multi-omics list input that is annotated so. Each ID j input must be a list form, i.e. enclosed as $\{ID_j\}$. If this is the case, MultipleList is set to True and each input list ID should have the form {ID,"Omics Type Label"}, e.g. {"NFKB1","Protein"}, and the different omics type are treated as different for each ID. If MultipleList is set to False, and labeled IDs are provided, labels corresponding to the same ID are treated as equivalent to avoid overcounting.
MultipleListCorrection	None	MultipleListCorrection is an option whether or not to correct for multi-omics analysis. The choices are None, Automatic, or a custom number. This essentially enlarges the population by this factor to account for additional IDs being considered as the result of a multi-omics cluster analysis. If the value is set to Automatic the number of unique ID labels is used to make the correction.
NonUCSC	False	NonUCSC option set to False assumes UCSC browser was used in determining an internal GeneDictionary used in ID translations where the KEGG identifiers for genes are number strings (e.g. 4790).<br>The NonUCSC option can be set to True if standard KEGG accessions are used in a user provided GeneDictionary variable, in the form OptionValue[KEGGOrganism] <>":"<>"number string", e.g. "hsa:4790"
OutputID	"KEGG Gene ID"	OutputID is a string to indicate the kind of ID to convert input genomic analysis entries. The default is "KEGG Gene ID" consistently with use of the "pathway" database as the default genomic analysis.

PathwayLengthFilter	2	PathwayLengthFilter can be used to set the value for which terms to consider in the computation, by excluding KEGG pathways that have fewer items compared to the PathwayLengthFilter value. It is used by the internal KEGGAnalysisAssigner function.
pValueCutoff	0.05	pValueCutoff provides a cutoff p–value for adjusted p–values to assess statistical significance.
ReportFilter	1	ReportFilter provides a cutoff for membership in pathways in selecting which terms/pathways to return. It is used in conjunction with ReportFilterFunction.
ReportFilterFunction	GreaterEqualThan	ReportFilterFunction specifies what operator form will be used to compare against ReportFilter option value in selecting which terms/pathways to return. The default is to use GreaterEqualThan
Species	"human"	The Species option specifies the species considered in the calculation.
TestFunction	N[1 - CDF[HypergeometricDistribution[#1, #2, #3], #4 - 1]] &	The TestFunction option calculates the p–values for the enrichment of each term. It can be a function of four inputs, f[#1,#2,#3,#4] (e.g. the default is using a hypergeometric distribution CDF, N[1–CDF[HypergeometricDistribution[#1,#2,#3],#4–1]]]. The four inputs refer to:<br>#1 is number of draws (members in group being tested),<br>#2 is number of successes for category in population,<br>#3 is total number of members in population,<br>#4 is number of successes (or more) in current group being tested for specific category.<br>The output is a p–value (real positive number ≤ 1).

Options for KEGGAnalysis .

The input data can be a single list of n genes in the form:

data = {ID_1, ID_2, ..., ID_n}

The IDs may be provided as ID strings, ID_j (e.g. "NFKB1") as strings enclosed in list brackets {ID_j}, (e.g. {"NFKB1"} or as labeled strings in the case of multiple omics being considered. Labeled IDs are typically provided as:

{{ID_1, ... optional label $items_1$, $label_1$},
{ID_2, ... optional label $items_2$, ..., $label_2$}, ... {ID_n, ..., optional label $items_n$, ..., $label_n$}}.

The ID labels are typically a string, e.g. typically "RNA" or "Protein", (e.g. {"NFKB1","Protein"}) or for a molecular ID obtained from metabolomics experiments, can also contain other optional label items such as mass and retention time {"cpd:C00449", 276.133, 11.0041, "Meta"}. The main label must always be the last element in the list.

The output has the following structures: for a single list input:

```
listOutput = <|KEGG : pathway1 →
    {{p - value1, multiple hypothesis adjusted p - value1, True / False for statistical significance},
     {{number of members in group being tested, number of successes for term1 in population,
        total number of members in population, number of members (or more) in current group being tested
         associated to pathway1}, {KEGG pathway1 description, {input IDs associated to pathway1}}}},
   KEGG : pathway2 → {{p - value2, multiple hypothesis adjusted p - value2,
       True / False for statistical significance}, {{number of members in group being tested,
        number of successes for term2 in population, total number of members in population,
        number of members (or more) in current group being tested associated to pathway2},
       {KEGG pathway1 description, {input IDs associated to pathway2}}}},  ..., KEGG : pathwayn →
    {{p - valuen, multiple hypothesis adjusted p - valuen, True / False for statistical significance},
     {{number of members in group being tested, number of successes for termn in population,
        total number of members in population, number of members (or more) in current group being tested
         associated to pathwayn}, {KEGG pathwayn description, {input IDs associated to pathwayn}}}}
  |>
```

The input data can also be an association of multiple `L` groups to be tested:

```
data = <|Group1 → {ID11, ID12, ..., ID1n1},
    Group2 → {ID21, ID22, ..., ID2n2}, ...,
    GroupL → {ID11, ID12, ..., ID1nL}|>.
```

In this case the output for each group has the `listOutput` format described above:

```
associationOutput = <|Group1 → listOutput1,
   Group2 → listOutput2, ...,
   GroupL → listOutputL|>
```

`KEGGAnalysis` can also take as input the output of clustering of time series classification data, e.g. `TimeSeriesClusters` or `TimeSeriesSingleClusters` association of associations. The groups for each class will then have keys labeled `"GroupAssociations"`, that include the labels used in the clustering. The labels must correspond to protein or gene accessions/IDs. For each class and group the corresponding KEGGAnalysis enrichment is computed and returned.

There are two types of analyses that are carried out, which can be set by the AnalysisType option value. The default "Genomic" analysis is based on input gene symbols. The "Molecular" analysis is based on molecular input accessions (e.g. compounds "cpd" databases). For multi-omic input the user may select to do All analyses. In this case an additional outer association is created with labels indicating each of "Genomic" or "Molecular" analysis carried out.

The enrichment analysis is an over-representation calculation, using a hypergeometric test. For a given a given group (e.g. members of a cluster after classification), we try to identify which KEGG pathway terms are over-representated by membership of IDs to that cluster. The KEGGAnalysis function allows us to select the background, and hence address selection bias. Additionally a Benjamini-Hochberg procedure false discovery rate (FDR) may be calculated for each representation.

We carry out our KEGGAnalysis for all the classes and groups/subgroups. We only report terms for which there are at least 2 members, and additionally correct for multiple omics (2 sets of KEGG terms, one each for proteomics and transcriptomics). Please note that this is a time consuming computation.

```
In[131]:= keggAnalysisCombined = KEGGAnalysis[combinedClusters,
             ReportFilter → 2, MultipleList → True, MultipleListCorrection → 2 , AnalysisType → All];
```

We see that both "Molecular" and "Genomic" analysis is performed:

```
In[132]:= Keys@keggAnalysisCombined

Out[132]= {Molecular, Genomic}
```

We can extract both Genomic and molecular analysis:

```
In[133]:= keggAnalysisCombined["Genomic"]
```

```
Out[133]= <|SpikeMax → <|G1S1 → <||>, G1S2 → <|path:hsa04330 → {{0.000270063, 0.00189044, True}, {4, 96, 14172, 2},
        {Notch signaling pathway - Homo sapiens (human), {{{NUMBL, RNA}}, {{HES1, RNA}}}}}|>,
    G1S3 → <||>, ··· 16 ···, G3S12 → <||>, G3S13 → <||>|>, ··· 7 ···, f7 → <| ··· 1 ··· |>|>

large output   show less   show more   show all   set size limit...
```

```
In[134]:= keggAnalysisCombined["Molecular"]
```

```
Out[134]= <|SpikeMax → <|G1S1 → <||>, G1S2 → <||>, G1S3 → <||>, G1S4 → <||>, G2S1 → <||>, G2S2 → <||>, G2S3 → <||>, G2S4 → <||>,
    G3S1 → <||>, G3S2 → <||>, G3S3 → <||>, G3S4 → <||>, G3S5 → <||>, G3S6 → <||>, G3S7 → <||>, G3S8 → <||>,
    G3S9 → <||>, G3S10 → <||>, G3S11 → <||>, G3S12 → <||>, G3S13 → <||>|>, SpikeMin → <|G1S1 → <||>, G1S2 → <||>,
    G2S1 → <|path:map00120 → {{0.00172071, 0.0275314, True}, {8, 47, 5841, 2}, {Primary bile acid biosynthesis,
          {{{cpd:C01921, 465.309, 11.8056, Meta}}, {{cpd:C05446, 436.355, 14.3015, Meta}}}}},
      path:map04976 → {{0.00730513, 0.0435924, True}, {8, 98, 5841, 2},
        {Bile secretion, {{{cpd:C04555, 368.165, 12.0826, Meta}, {cpd:C04555, 368.166, 12.6899, Meta},
            {cpd:C04555, 368.166, 12.3718, Meta}}, {{cpd:C01921, 465.309, 11.8056, Meta}}}}}|>,
    G3S1 → <||>, G3S2 → <||>|>, f1 → <|G1S1 → <||>, G1S2 → <||>|>, f2 → <|G1S1 → <||>, G1S2 → <||>,
    G2S1 → <||>|>,
  f3 → <|G1S1 → <||>, G1S2 → <||>, G2S1 → <||>|>,
  f4 →
   <|G1S1 → <||>, G1S2 → <||>, G2S1 → <||>, G2S2 → <||>, G3S1 → <||>, G3S2 → <||>|>,
  f5 → <|G1S1 → <||>, G1S2 → <||>, G2S1 → <||>, G2S2 → <||>, G3S1 → <||>, G3S2 → <||>,
    G4S1 → <||>, G4S2 → <||>, G4S3 → <||>, G5S1 → <||>, G5S2 → <||>|>,
  f6 → <|G1S1 → <||>, G1S2 → <||>, G2S1 → <||>|>,
  f7 → <|G1S1 → <||>, G1S2 → <||>, G2S1 → <||>|>|>
```

Let us extract the names of the pathways found for the "SpikeMin" data:

```
In[135]:= Query["SpikeMin", All, All, 3, 1]@keggAnalysisCombined["Genomic"]
```

```
Out[135]= <|G1S1 → <|path:hsa03010 → Ribosome - Homo sapiens (human),
    path:hsa04270 → Vascular smooth muscle contraction - Homo sapiens (human)|>, G1S2 → <||>,
  G2S1 → <|path:hsa04662 → B cell receptor signaling pathway - Homo sapiens (human),
    path:hsa05161 → Hepatitis B - Homo sapiens (human),
    path:hsa05142 → Chagas disease (American trypanosomiasis) - Homo sapiens (human),
    path:hsa05200 → Pathways in cancer - Homo sapiens (human),
    path:hsa04120 → Ubiquitin mediated proteolysis - Homo sapiens (human),
    path:hsa04144 → Endocytosis - Homo sapiens (human), path:hsa04142 → Lysosome - Homo sapiens (human),
    path:hsa04620 → Toll-like receptor signaling pathway - Homo sapiens (human),
    path:hsa05132 → Salmonella infection - Homo sapiens (human),
    path:hsa05215 → Prostate cancer - Homo sapiens (human),
    path:hsa04010 → MAPK signaling pathway - Homo sapiens (human),
    path:hsa05120 → Epithelial cell signaling in Helicobacter pylori infection - Homo sapiens (human),
    path:hsa05162 → Measles - Homo sapiens (human),
    path:hsa04722 → Neurotrophin signaling pathway - Homo sapiens (human),
    path:hsa04071 → Sphingolipid signaling pathway - Homo sapiens (human),
    path:hsa04660 → T cell receptor signaling pathway - Homo sapiens (human),
    path:hsa05169 → Epstein-Barr virus infection - Homo sapiens (human),
    path:hsa04062 → Chemokine signaling pathway - Homo sapiens (human),
    path:hsa04210 → Apoptosis - Homo sapiens (human),
    path:hsa01521 → EGFR tyrosine kinase inhibitor resistance - Homo sapiens (human),
    path:hsa05145 → Toxoplasmosis - Homo sapiens (human),
    path:hsa05212 → Pancreatic cancer - Homo sapiens (human),
    path:hsa04066 → HIF-1 signaling pathway - Homo sapiens (human),
    path:hsa04621 → NOD-like receptor signaling pathway - Homo sapiens (human),
    path:hsa04668 → TNF signaling pathway - Homo sapiens (human),
    path:hsa05205 → Proteoglycans in cancer - Homo sapiens (human),
    path:hsa05220 → Chronic myeloid leukemia - Homo sapiens (human),
    path:hsa05166 → HTLV-I infection - Homo sapiens (human),
```

path:hsa04912 → GnRH signaling pathway - Homo sapiens (human),
path:hsa04380 → Osteoclast differentiation - Homo sapiens (human),
path:hsa05223 → Non-small cell lung cancer - Homo sapiens (human),
path:hsa04064 → NF-kappa B signaling pathway - Homo sapiens (human),
path:hsa04666 → Fc gamma R-mediated phagocytosis - Homo sapiens (human),
path:hsa04611 → Platelet activation - Homo sapiens (human),
path:hsa05164 → Influenza A - Homo sapiens (human),
path:hsa04211 → Longevity regulating pathway - Homo sapiens (human),
path:hsa04810 → Regulation of actin cytoskeleton - Homo sapiens (human),
path:hsa05231 → Choline metabolism in cancer - Homo sapiens (human),
path:hsa05140 → Leishmaniasis - Homo sapiens (human), path:hsa05131 → Shigellosis - Homo sapiens (human),
path:hsa04068 → FoxO signaling pathway - Homo sapiens (human),
path:hsa04012 → ErbB signaling pathway - Homo sapiens (human),
path:hsa05110 → Vibrio cholerae infection - Homo sapiens (human),
path:hsa05152 → Tuberculosis - Homo sapiens (human),
path:hsa05203 → Viral carcinogenesis - Homo sapiens (human),
path:hsa04664 → Fc epsilon RI signaling pathway - Homo sapiens (human),
path:hsa04014 → Ras signaling pathway - Homo sapiens (human),
path:hsa05160 → Hepatitis C - Homo sapiens (human),
path:hsa03440 → Homologous recombination - Homo sapiens (human),
path:hsa05133 → Pertussis - Homo sapiens (human), path:hsa03450 →
Non-homologous end-joining - Homo sapiens (human), path:hsa05214 → Glioma - Homo sapiens (human),
path:hsa04915 → Estrogen signaling pathway - Homo sapiens (human),
path:hsa04725 → Cholinergic synapse - Homo sapiens (human),
path:hsa05130 → Pathogenic Escherichia coli infection - Homo sapiens (human),
path:hsa04110 → Cell cycle - Homo sapiens (human),
path:hsa04917 → Prolactin signaling pathway - Homo sapiens (human),
path:hsa05211 → Renal cell carcinoma - Homo sapiens (human),
path:hsa05213 → Endometrial cancer - Homo sapiens (human),
path:hsa04520 → Adherens junction - Homo sapiens (human),
path:hsa05168 → Herpes simplex infection - Homo sapiens (human),
path:hsa04650 → Natural killer cell mediated cytotoxicity - Homo sapiens (human),
path:hsa04150 → mTOR signaling pathway - Homo sapiens (human),
path:hsa04213 → Longevity regulating pathway - multiple species - Homo sapiens (human),
path:hsa04145 → Phagosome - Homo sapiens (human),
path:hsa04330 → Notch signaling pathway - Homo sapiens (human),
path:hsa04670 → Leukocyte transendothelial migration - Homo sapiens (human),
path:hsa01100 → Metabolic pathways - Homo sapiens (human),
path:hsa04640 → Hematopoietic cell lineage - Homo sapiens (human),
path:hsa04730 → Long-term depression - Homo sapiens (human),
path:hsa04933 → AGE-RAGE signaling pathway in diabetic complications - Homo sapiens (human),
path:hsa04962 → Vasopressin-regulated water reabsorption - Homo sapiens (human),
path:hsa01522 → Endocrine resistance - Homo sapiens (human),
path:hsa05210 → Colorectal cancer - Homo sapiens (human),
path:hsa05222 → Small cell lung cancer - Homo sapiens (human),
path:hsa05221 → Acute myeloid leukemia - Homo sapiens (human),
path:hsa04728 → Dopaminergic synapse - Homo sapiens (human),
path:hsa04151 → PI3K-Akt signaling pathway - Homo sapiens (human),
path:hsa04540 → Gap junction - Homo sapiens (human),
path:hsa00562 → Inositol phosphate metabolism - Homo sapiens (human),
path:hsa04918 → Thyroid hormone synthesis - Homo sapiens (human),
path:hsa04720 → Long-term potentiation - Homo sapiens (human),
path:hsa03430 → Mismatch repair - Homo sapiens (human),
path:hsa04070 → Phosphatidylinositol signaling system - Homo sapiens (human),
path:hsa04960 → Aldosterone-regulated sodium reabsorption - Homo sapiens (human),
path:hsa04919 → Thyroid hormone signaling pathway - Homo sapiens (human),
path:hsa04910 → Insulin signaling pathway - Homo sapiens (human),
path:hsa01200 → Carbon metabolism - Homo sapiens (human),
path:hsa04622 → RIG-I-like receptor signaling pathway - Homo sapiens (human),
path:hsa04931 → Insulin resistance - Homo sapiens (human),
path:hsa00512 → Mucin type O-Glycan biosynthesis - Homo sapiens (human),
path:hsa04350 → TGF-beta signaling pathway - Homo sapiens (human),
path:hsa05100 → Bacterial invasion of epithelial cells - Homo sapiens (human),
path:hsa05340 → Primary immunodeficiency - Homo sapiens (human),
path:hsa04750 → Inflammatory mediator regulation of TRP channels - Homo sapiens (human),
path:hsa04630 → Jak-STAT signaling pathway - Homo sapiens (human),

[illegible]
path:hsa05134 → Legionellosis - Homo sapiens (human),
path:hsa04966 → Collecting duct acid secretion - Homo sapiens (human),
path:hsa04530 → Tight junction - Homo sapiens (human),
path:hsa03410 → Base excision repair - Homo sapiens (human),
path:hsa04510 → Focal adhesion - Homo sapiens (human),
path:hsa01524 → Platinum drug resistance - Homo sapiens (human),
path:hsa04320 → Dorso-ventral axis formation - Homo sapiens (human) |⟩,
G3S1 → ⟨| path:hsa01100 → Metabolic pathways - Homo sapiens (human),
path:hsa05169 → Epstein-Barr virus infection - Homo sapiens (human),
path:hsa03040 → Spliceosome - Homo sapiens (human),
path:hsa05016 → Huntington's disease - Homo sapiens (human),
path:hsa01200 → Carbon metabolism - Homo sapiens (human),
path:hsa00230 → Purine metabolism - Homo sapiens (human),
path:hsa05010 → Alzheimer's disease - Homo sapiens (human),
path:hsa04660 → T cell receptor signaling pathway - Homo sapiens (human),
path:hsa04142 → Lysosome - Homo sapiens (human),
path:hsa00240 → Pyrimidine metabolism - Homo sapiens (human),
path:hsa04120 → Ubiquitin mediated proteolysis - Homo sapiens (human),
path:hsa00510 → N-Glycan biosynthesis - Homo sapiens (human),
path:hsa05012 → Parkinson's disease - Homo sapiens (human),
path:hsa04910 → Insulin signaling pathway - Homo sapiens (human),
path:hsa04722 → Neurotrophin signaling pathway - Homo sapiens (human),
path:hsa03030 → DNA replication - Homo sapiens (human), path:hsa04210 → Apoptosis - Homo sapiens (human),
path:hsa04932 → Non-alcoholic fatty liver disease (NAFLD) - Homo sapiens (human),
path:hsa04662 → B cell receptor signaling pathway - Homo sapiens (human),
path:hsa05220 → Chronic myeloid leukemia - Homo sapiens (human),
path:hsa00280 → Valine, leucine and isoleucine degradation - Homo sapiens (human),
path:hsa00190 → Oxidative phosphorylation - Homo sapiens (human),
path:hsa04146 → Peroxisome - Homo sapiens (human),
path:hsa00520 → Amino sugar and nucleotide sugar metabolism - Homo sapiens (human),
path:hsa03020 → RNA polymerase - Homo sapiens (human),
path:hsa00051 → Fructose and mannose metabolism - Homo sapiens (human),
path:hsa03050 → Proteasome - Homo sapiens (human),
path:hsa00562 → Inositol phosphate metabolism - Homo sapiens (human),
path:hsa05210 → Colorectal cancer - Homo sapiens (human),
path:hsa05131 → Shigellosis - Homo sapiens (human),
path:hsa04666 → Fc gamma R-mediated phagocytosis - Homo sapiens (human),
path:hsa04130 → SNARE interactions in vesicular transport - Homo sapiens (human),
path:hsa05221 → Acute myeloid leukemia - Homo sapiens (human),
path:hsa04110 → Cell cycle - Homo sapiens (human),
path:hsa04650 → Natural killer cell mediated cytotoxicity - Homo sapiens (human),
path:hsa00020 → Citrate cycle (TCA cycle) - Homo sapiens (human),
path:hsa05161 → Hepatitis B - Homo sapiens (human),
path:hsa00630 → Glyoxylate and dicarboxylate metabolism - Homo sapiens (human),
path:hsa01230 → Biosynthesis of amino acids - Homo sapiens (human),
path:hsa04070 → Phosphatidylinositol signaling system - Homo sapiens (human),
path:hsa04370 → VEGF signaling pathway - Homo sapiens (human),
path:hsa05152 → Tuberculosis - Homo sapiens (human),
path:hsa03420 → Nucleotide excision repair - Homo sapiens (human),
path:hsa04012 → ErbB signaling pathway - Homo sapiens (human),
path:hsa03410 → Base excision repair - Homo sapiens (human),
path:hsa05130 → Pathogenic Escherichia coli infection - Homo sapiens (human),
path:hsa05213 → Endometrial cancer - Homo sapiens (human),
path:hsa04071 → Sphingolipid signaling pathway - Homo sapiens (human),
path:hsa00640 → Propanoate metabolism - Homo sapiens (human),
path:hsa04064 → NF-kappa B signaling pathway - Homo sapiens (human),
path:hsa01212 → Fatty acid metabolism - Homo sapiens (human),
path:hsa00480 → Glutathione metabolism - Homo sapiens (human),
path:hsa04664 → Fc epsilon RI signaling pathway - Homo sapiens (human),
path:hsa05166 → HTLV-I infection - Homo sapiens (human),
path:hsa01524 → Platinum drug resistance - Homo sapiens (human),
path:hsa04066 → HIF-1 signaling pathway - Homo sapiens (human),
path:hsa05212 → Pancreatic cancer - Homo sapiens (human),
path:hsa00030 → Pentose phosphate pathway - Homo sapiens (human),
path:hsa05211 → Renal cell carcinoma - Homo sapiens (human),

```
path:hsa05214 → Glioma - Homo sapiens (human),
path:hsa04152 → AMPK signaling pathway - Homo sapiens (human), path:hsa05162 →
 Measles - Homo sapiens (human), path:hsa00052 → Galactose metabolism - Homo sapiens (human),
path:hsa00071 → Fatty acid degradation - Homo sapiens (human),
path:hsa00010 → Glycolysis / Gluconeogenesis - Homo sapiens (human), path:hsa00532 →
 Glycosaminoglycan biosynthesis - chondroitin sulfate / dermatan sulfate - Homo sapiens (human),
path:hsa01040 → Biosynthesis of unsaturated fatty acids - Homo sapiens (human),
path:hsa03430 → Mismatch repair - Homo sapiens (human),
path:hsa05100 → Bacterial invasion of epithelial cells - Homo sapiens (human),
path:hsa04144 → Endocytosis - Homo sapiens (human),
path:hsa00533 → Glycosaminoglycan biosynthesis - keratan sulfate - Homo sapiens (human),
path:hsa05215 → Prostate cancer - Homo sapiens (human),
path:hsa04810 → Regulation of actin cytoskeleton - Homo sapiens (human),
path:hsa01210 → 2-Oxocarboxylic acid metabolism - Homo sapiens (human),
path:hsa04611 → Platelet activation - Homo sapiens (human),
path:hsa00310 → Lysine degradation - Homo sapiens (human),
path:hsa00970 → Aminoacyl-tRNA biosynthesis - Homo sapiens (human),
path:hsa05223 → Non-small cell lung cancer - Homo sapiens (human),
path:hsa04062 → Chemokine signaling pathway - Homo sapiens (human),
path:hsa00620 → Pyruvate metabolism - Homo sapiens (human),
path:hsa05230 → Central carbon metabolism in cancer - Homo sapiens (human),
path:hsa04380 → Osteoclast differentiation - Homo sapiens (human),
path:hsa04668 → TNF signaling pathway - Homo sapiens (human),
path:hsa00563 → Glycosylphosphatidylinositol(GPI)-anchor biosynthesis - Homo sapiens (human),
path:hsa01522 → Endocrine resistance - Homo sapiens (human),
path:hsa00270 → Cysteine and methionine metabolism - Homo sapiens (human),
path:hsa03022 → Basal transcription factors - Homo sapiens (human),
path:hsa03060 → Protein export - Homo sapiens (human),
path:hsa04620 → Toll-like receptor signaling pathway - Homo sapiens (human),
path:hsa04622 → RIG-I-like receptor signaling pathway - Homo sapiens (human),
path:hsa04623 → Cytosolic DNA-sensing pathway - Homo sapiens (human)|>, G3S2 → <||>|>
```

The results from a MathIOmica time series clustering enrichment analysis can be exported to spreadsheets using `EnrichmentReportExport`.

`EnrichmentReportExport`[*results*]	exports results from enrichment analyses to Excel spreadsheets, particularly suited for exporting multi–omics `TimeSeriesClusters` enrichment analysis results (via `KEGGAnalysis` or `GOAnalysis`). An excel spreadsheet is generated for each Class, named after the Class key, with sheets created for and named after each Group in that Class containing the enrichment output for that Group.

Exporting the enrichment analysis results to spreadsheets.

option name	default value	
`AppendString`	""	String that will be appended to the file name after the class name. If a string is not provided the current Date is appended.
`OutputDirectory`	None	`OutputDirectory` specifies the location of a directory to output the Excel spreadsheets generated by the function. If it is set to None the `NotebookDirectory[]` will be used as a default output directory.

Options for `EnrichmentReportExport`.

We can export the reports, for example to the `$UserDocumentDirectory`:

```
In[136]:= EnrichmentReportExport[keggAnalysisCombined["Genomic"],
  OutputDirectory → $UserDocumentsDirectory, AppendString → "KEGGAnalysisCombined"];
```

We can export the GO analysis results as well, for example to the `$UserDocumentDirectory`:

```
In[137]:= EnrichmentReportExport[goAnalysisCombined,
  OutputDirectory → $UserDocumentsDirectory, AppendString → "GOAnalysisCombined"];
```

Visualization of Pathways from KEGG

MathIOmica allows visualization and coloring of KEGG pathways using `KEGGPathwayVisual`.

`KEGGPathwayVisual[pathway]`	generates a visual representation for a KEGG: Kyoto Encyclopedia of Genes and Genomes *pathway*.

Visualizing KEGG pathways.

option name	default value	
AnalysisType	"Genomic"	AnalysisType provides a selection for the type of analysis to perform. "Genomic" analysis (default) uses gene identifier based pathway visualization. "Molecular" analysis uses molecular analysis map visualization.
AugmentDictionary	True	AugmentDictionary provides a choice whether or not to augment the current `ConstantGeneDictionary` variable or create a new one.
BlendColors	{RGBColor[0, 0, 1], RGBColor[0, 0, 1], RGBColor[0.5, 0.5, 0.5], RGBColor[1, 0, 0], RGBColor[1, 0, 0]}	BlendColors provides a list of colors to be used in coloring intensities provided and is used by the IntensityFunction as its first argument. The colors must be provided as RGBColor[] specification.
ColorSelection	<|"RNA" → "bg", "Protein" → "fg"|>	ColorSelection assigns foreground and background colors in the KEGG pathway through an association. The Keys point to labels for multi–omics data, and the values "bg" and "fg" can point to background and foreground representations respectively for each key.
DefaultColors	{"fg" -> RGBColor[0, 0, 0], "bg" -> RGBColor[0, 1, 0]}	DefaultColors provides a list of rules for setting the colors to be used as default values for the fore–ground "fg" and background "bg" respectively in the generated pathways. The colors must be provided as RGBColor[] specification.
ExportMovieOptions	{"VideoEncoding"→"MPEG-4 Video", "FrameRate"→1}	ExportMovieOptions provides options for the Export function used internally to export the pathway list when Intensities have been provided for a time series representation of data.
FileExtend	".mov"	FileExtend provides a string to be appended to the file name if the ResultsFormat is set to "Movie".

GeneDictionary	None	GeneDictionary points to an existing variable to use as a gene dictionary in annotations. The gene dictionary is used to convert MemberSet identities provided to corresponding KEGG identifiers. If GeneDictionary is set to None the default ConstantGeneDictionary will be created or augmented through the use of GetGeneDictionary .
GetGeneDicitonaryOptions	{}	The GetGeneDictionaryOptions option specifies a list of options that will be passed to the internal GetGeneDictionary function.
InputID	{"UniProt ID", "Gene Symbol"}	The InputID option specifies the kind of identifiers/accessions used as input when identifiers are provided through setting the MemberSet values.
Intensities	None	Intensities may be used to provide a set of intensities that will be used for coloring components of the pathway. The intensities are provided as an association for each ID as single values, or as a list of values in the case of series data: <| ID_1 → {intensity list for ID_1}, ID_2 → {intensity list for ID_2}, ..., ID_N → {intensity list for ID_N}|>. Intensities must be scaled from −1 to 1, or selected such that the IntensityFunction can convert them to a number between 0 to 1.
IntensityFunction	(Blend[#1, (#2+1)/2]&)	IntensityFunction is a function of two arguments that allows customization of the coloring for the intensities. The IntensityFunction value can be any function which outputs a color, I(#1,#2), (*where#1 is the BlendColors option value, and #2 is an intensity vector, that has values typically ranging from [−1,1].
KEGGAnalysisAssignerOptions	{}	The KEGGAnalysisAssignerOptions option specifies a list of options that will be passed to the internal KEGGAnalysisAssigner function.
KEGGDatabase	"pathway"	KEGGDatabase value indicates which KEGG database to use as the target database.
KEGGMolecular	"cpd"	KEGGMolecular specifies which database to use for molecular analysis. The default is the compound database ("cpd").
KEGGOrganism	"hsa"	KEGGOrganism indicates which organism (org) to use for "Genomic" type of analysis. The default is human analysis org="hsa".
MathIOmicaDataDirectory	ConstantMathIOmica-DataDirectory	MathIOmicaDataDirectory option specifies the directory where the default MathIOmica package data are stored. By default the option is set to create the standard directory if it does not exist already.

MemberSet	All	MemberSet selects which members of the pathway are to be considered. The choices are:<br>All: return the pathway only.<br>{list of identifiers}: a list of identifiers that will be highlighted. If ORA is set to True the list must be the output from an over representation analysis, and the identifiers will be selected from the last list, second sublist.<br>Only IDs that are found to match in the pathway are colored.<br>An internal gene dictionary (see GetGeneDictionary) is used to convert IDs to KEGG IDs.
MissingValueColor	RGBColor[0.4, 0.4, 0.4]	MissingValueColor provides a color to be used when Intensities are provided to represent values that are tagged as Missing[]. The color must be provided as RGBColor[] specification.
MolecularInputID	{"cpd"}	MolecularInputID is a string list to indicate the kind of ID to use for the input molecule entries.
MolecularOutputID	"cpd"	MolecularOutputID is a string to indicate the kind of ID to convert input molecule entries. The default is "cpd" consistently with use of the "cpd" database as the default molecular analysis.
MolecularSpecies	"compound"	MolecularSpecies specifies the kind of molecular input.
MovieFilePath	None	MovieFilePath indicates the path (including file name) where if ResultsFormat is set to "Movie" the movie generated will be saved. The default value None will generate a file named after the pathway with extension set by the FileExtend option in the current directory.
NonUCSC	False	NonUCSC option set to False assumes UCSC browser was used in determining an internal GeneDictionary used in ID translations where the KEGG identifiers for genes are number strings (e.g. 4790).<br>The NonUCSC option can be set to True if standard KEGG accessions are used in a user provided GeneDictionary variable, in the form OptionValue[KEGGOrganism] <>":"<>"number string", e.g. "hsa:4790"
ORA	False	ORA can be set to True or False depending on whether the input is from an over representation analysis (e.g. output from KEGGAnalysis), or not respectively.
OutputID	"KEGG Gene ID"	OutputID is a string to indicate the kind of ID to convert input genomic analysis entries. The default is "KEGG Gene ID" consistently with use of the "pathway" database as the default genomic analysis.
ResultsFormat	"URL"	ResultsFormat provides a choice of output format, the choices are:<br>"URL": returns a URL of the pathway,<br>"Figure": returns figure output(s) for the pathway,<br>"Movie": in the case of series data returns a movie/animation of the series pathway snapshots.

SingleColorPlace	"bg"	SingleColorPlace selects in the case of a single identifier input whether to place the color to the foreground, ("fg") or background ("bg" set by default).
Species	"human"	The Species option specifies the species considered in the calculation.
StandardHighlight	{"fg" -> RGBColor[1, 0, 0], "bg" -> RGBColor[0.5, 0.7, 1]}	StandardHighlight provides a list of rules for setting the highlight colors for the IDs represented in the pathway (when no intensities are provided). The list specifies color rules for foregroung, "fg", and background, "bg", respectively. The colors must be provided as RGBColor[] specification.

Options for KEGGPathwayVisual .

ResultsFormat option setting	"Results" value for returned data
"URL"	Browser URL pointing to pathway on KEGG database, or if a list of Intensities was provided a series of URLs corresponding to each time point or sequential data in the series.
"Figure"	Pathway figure downloaded from the KEGG database, or if a list of Intensities was provided a series of figures corresponding to each time point or sequential data in the series.
"Movie"	Name of the output file that contains the generated movie/animation that is based on the list of Intensities provided.

ResultsFormat option output for KEGGPathwayVisual

For example, we can look at the B-cell receptor pathway:

In[138]:=
```
exampleBCellReceptor = KEGGPathwayVisual["path:hsa04662"]
```

Out[138]=
```
<|Pathway → path:hsa04662, Results → {http://www.kegg.jp/kegg-bin/show_pathway?map=hsa04662}|>
```

We can open this in a browser:

In[139]:=
```
SystemOpen[exampleBCellReceptor["Results"][[1]]]
```

We can import directly the pathway:

In[140]:=
```
exampleBCellReceptorFigure = KEGGPathwayVisual["path:hsa04662", ResultsFormat → "Figure"]
```

Out[140]= <|Pathway → path:hsa04662, Results → {

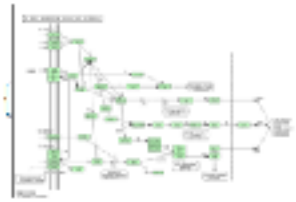

}|>

We can zoom in:

In[141]:= `Show[exampleBCellReceptorFigure["Results"][[1]], ImageSize → 500]`

Out[141]=

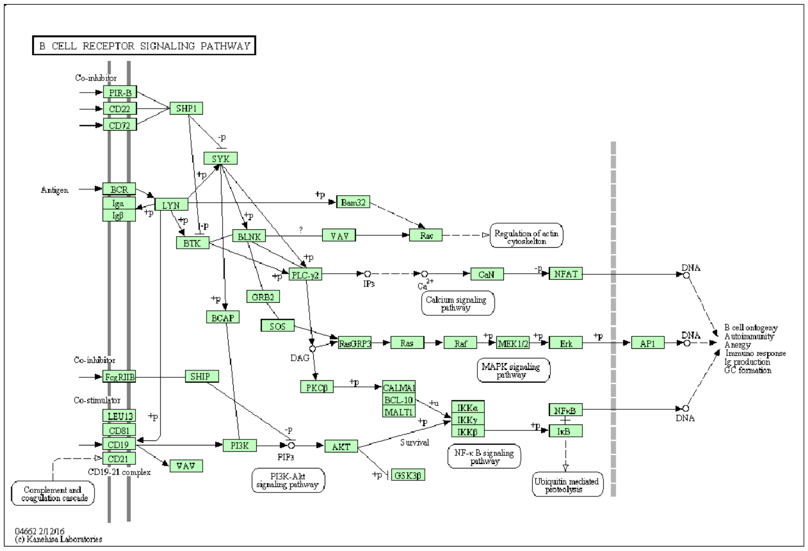

We can highlight the components:

In[154]:= `exampleBCellReceptorFigureHighlight = KEGGPathwayVisual["path:hsa04662", ResultsFormat → "Figure", MemberSet → Query["SpikeMin", 3, 1]@keggAnalysisCombined["Genomic"], ORA → True]`

Out[154]=

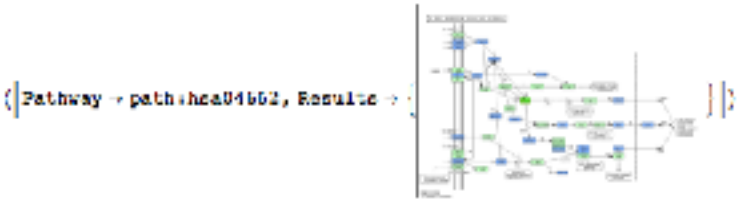

We can zoom in:

In[155]:= `Show[exampleBCellReceptorFigureHighlight["Results"][[1]], ImageSize → 500]`

Out[155]=

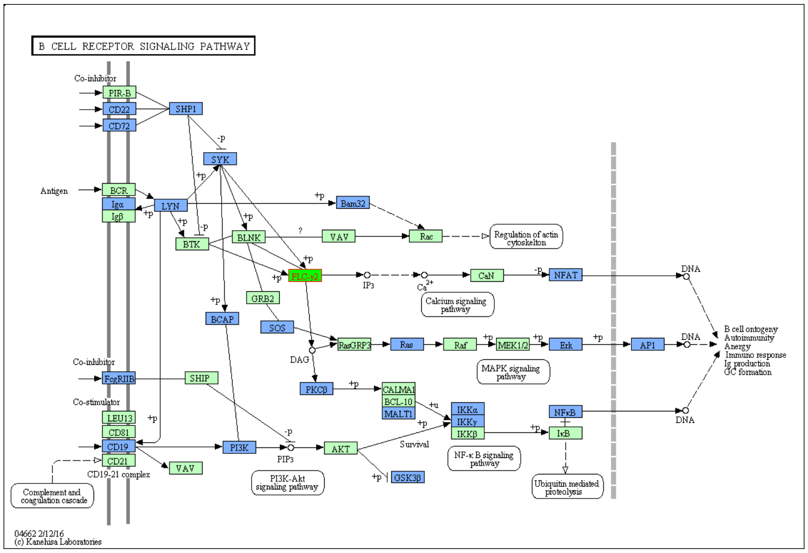

We can also create snapshots and an animation of this data.

First, let's extract the members of the pathway in the analysis:

```
In[156]:= membersBCellReceptor = (Query["SpikeMin", 3, 1, 3, 2]@keggAnalysisCombined["Genomic"])[[All, 1]]

Out[156]= {{CD72, RNA}, {CD19, RNA}, {CD22, RNA}, {IKBKG, RNA}, {CD79A, RNA}, {PIK3R1, RNA}, {PIK3CG, RNA},
 {MAPK1, RNA}, {PRKCB, RNA}, {CHUK, RNA}, {SOS1, RNA}, {MALT1, RNA}, {NFATC2, RNA}, {KRAS, RNA}, {NRAS, RNA},
 {NFKB1, RNA}, {DAPP1, RNA}, {P16885, Protein}, {JUN, RNA}, {FOS, RNA}, {NFATC3, RNA}, {SOS2, RNA},
 {GSK3B, RNA}, {PIK3CA, RNA}, {SYK, RNA}, {PIK3AP1, RNA}, {LYN, RNA}, {FCGR2B, RNA}, {PTPN6, RNA}}
```

First, let's extract the members of the pathway in the analysis:

```
In[157]:= intensitiesRNABCellReceptor = DeleteMissing[Query[Key[#] & /@ membersBCellReceptor] @ rnaFinalTimeSeries];
intensitiesproteinBCellReceptor =
  DeleteMissing[Query[Key[#] & /@ membersBCellReceptor] @ proteinFinalTimeSeries];
intensitiesAll = Join[intensitiesRNABCellReceptor, intensitiesproteinBCellReceptor]
```

```
Out[159]= <|{CD72, RNA} → {0.369636, 0., 0.261993, 0.0820577, 0.224604, 0.0603929, 0.192008,
    0.260405, -0.693646, -0.0767903, -0.0257001, 0.168882, 0.282635, 0.174763, 0.0507434},
  {CD19, RNA} → {0.172249, 0., 0.258907, 0.0927547, 0.159745, 0.143044, 0.190808, 0.164894,
    -0.764163, 0.0772221, 0.123483, 0.16204, 0.338617, 0.161597, 0.113703},
  {CD22, RNA} → {0.155878, 0., 0.232584, 0.0114955, 0.149804, 0.0200342, 0.150032,
    0.219199, -0.799313, 0.07276, 0.212261, 0.121103, 0.26138, 0.23603, 0.0113412},
  {IKBKG, RNA} → {0.131827, 0., 0.105417, 0.140722, 0.235222, 0.147945, -0.0841993, 0.0529117,
    -0.888898, 0.0401513, 0.07162, 0.161643, 0.0883864, -0.124592, -0.135113},
  {CD79A, RNA} → {-0.189432, 0., -0.175651, -0.28551, -0.2934, -0.174296, -0.0405483,
    -0.0199819, -0.831633, -0.0175177, 0.0924073, -0.118086, 0.0441898, 0.128381, 0.0200428},
  {PIK3R1, RNA} → {-0.106517, 0., -0.17533, -0.296155, -0.141936, -0.0746977, -0.0847119,
    -0.155054, -0.569881, -0.0518873, -0.286679, -0.252989, -0.296549, -0.38416, -0.322641},
  {PIK3CG, RNA} → {-0.158765, 0., -0.239046, -0.245415, -0.189176, -0.121221, -0.0934737,
    -0.225435, -0.437243, -0.0514633, -0.334416, -0.292936, -0.270547, -0.407895, -0.341075},
  {MAPK1, RNA} → {-0.112538, 0., -0.193801, -0.188377, -0.15521, -0.116643, -0.139735,
    -0.179571, -0.536617, -0.0987596, -0.293139, -0.29907, -0.265028, -0.427059, -0.314833},
  {PRKCB, RNA} → {-0.140604, 0., -0.195521, -0.235728, -0.136136, -0.181346, -0.19531,
    -0.223034, -0.484427, -0.121888, -0.302139, -0.297782, -0.318102, -0.359512, -0.295294},
  {CHUK, RNA} → {-0.120907, 0., -0.15677, -0.137952, -0.0930449, -0.0225444, -0.0361759,
    -0.110594, -0.512861, -0.00526353, -0.362858, -0.330897, -0.264454, -0.480021, -0.338377},
  {SOS1, RNA} → {-0.0606504, 0., -0.111287, -0.178781, -0.0982303, -0.136136, -0.0846214,
    -0.134155, -0.586671, -0.0780006, -0.291524, -0.308802, -0.29339, -0.383405, -0.367348},
  {MALT1, RNA} → {-0.125888, 0., -0.101329, -0.21489, -0.108416, -0.0884985, -0.102949,
    -0.11088, -0.605948, -0.022327, -0.241551, -0.307442, -0.311018, -0.381066, -0.350352},
  {NFATC2, RNA} → {-0.104342, 0., -0.243953, -0.423115, -0.199442, -0.147515, -0.0916005,
    -0.183006, -0.424971, -0.0677965, -0.279945, -0.212719, -0.295223, -0.398534, -0.303897},
  {KRAS, RNA} → {-0.147324, 0., -0.158095, -0.164835, -0.0995704, -0.0135417, 0.00217258,
    -0.109387, -0.635953, 0.0117908, -0.337228, -0.278949, -0.253493, -0.394209, -0.297144},
  {NRAS, RNA} → {-0.178825, 0., -0.212762, -0.184755, -0.15832, 0.0157535, 0.0122249, -0.187022,
    -0.561628, 0.0714721, -0.267732, -0.298607, -0.251936, -0.432289, -0.310569},
  {NFKB1, RNA} → {-0.185885, 0., -0.1307, -0.13342, -0.147088, 0.021066, -0.00359583,
    -0.0668426, -0.594161, -0.0765257, -0.34297, -0.185951, -0.22097, -0.404316, -0.424988},
  {DAPP1, RNA} → {0.0520285, 0., -0.13698, -0.0284381, -0.0512643, -0.0872277, -0.0297875,
    -0.139989, -0.780904, -0.0375401, -0.35231, -0.1713, -0.278768, -0.192916, -0.259589},
  {JUN, RNA} → {-0.424343, 0., -0.21732, 0.145587, 0.168895, -0.0237648, -0.249424, -0.195597,
    -0.0490174, -0.0730759, 0.118224, -0.420685, -0.321629, -0.366567, -0.430624},
  {FOS, RNA} → {-0.140955, 0., -0.211122, 0.0904651, 0.0962169, -0.0509185, -0.106822,
    -0.321221, -0.187712, -0.196718, -0.361961, -0.365164, -0.319539, -0.328524, -0.502597},
  {NFATC3, RNA} → {-0.0651857, 0., -0.369667, -0.571392, -0.197483, -0.0939828, 0.264015,
    -0.291313, -0.288798, 0.116837, -0.0860154, -0.0699613, -0.0924046, -0.460048, -0.0260302},
  {SOS2, RNA} → {-0.0877297, 0., -0.159281, -0.173221, -0.143391, -0.139202, -0.0968374,
    -0.163688, -0.464941, -0.0838152, -0.325788, -0.403366, -0.277122, -0.423623, -0.335453},
  {GSK3B, RNA} → {-0.13407, 0., -0.131964, -0.177952, -0.140756, -0.133105, -0.104376, -0.16351,
    -0.524221, -0.0780582, -0.291452, -0.326255, -0.312979, -0.418583, -0.335157},
  {PIK3CA, RNA} → {-0.0806376, 0., -0.131049, -0.198564, -0.101695, -0.0233558, -0.0230476,
    -0.102728, -0.524415, -0.0617142, -0.341706, -0.246028, -0.302644, -0.418104, -0.438642},
  {SYK, RNA} → {-0.101852, 0., -0.124811, -0.0403809, -0.129592, -0.0922402, -0.102789,
    -0.154618, -0.435298, -0.082928, -0.36212, -0.403721, -0.257497, -0.433169, -0.410146},
  {PIK3AP1, RNA} → {-0.106304, 0., -0.162172, -0.0574579, -0.0868849, -0.0842937, -0.0780237,
    -0.194464, -0.480954, -0.0515741, -0.303023, -0.389961, -0.259407, -0.449938, -0.391068},
  {LYN, RNA} → {-0.0499215, 0., -0.184941, 0.064726, -0.0729396, -0.0748165, -0.0733593,
    -0.215647, -0.450748, -0.00640494, -0.353891, -0.408684, -0.193201, -0.498398, -0.339276},
  {FCGR2B, RNA} → {0.0304604, 0., 0.0526883, 0.285755, 0.0131493, 0.0632271, 0.0761535,
    0.0337862, -0.82869, 0.0258942, -0.168364, -0.280762, -0.0882869, -0.25397, -0.191474},
  {PTPN6, RNA} → {-0.0374181, 0., 0.116968, 0.180645, 0.159753, 0.0516791, -0.10847, 0.0037562,
    -0.751868, -0.169679, -0.273978, -0.191332, 0.140941, -0.292848, -0.317682},
  {P16885, Protein} → {-0.0327188, 0., -0.212903, 0.160126, -0.189055, -0.0720865, 0.118981,
    -0.0828107, -0.787692, -0.0359545, -0.200248, Missing[], -0.395315, -0.0492386, -0.213213}|>
```

We can now extract and plot the sequence of figures:

```
In[160]:= exampleBCellReceptorFigureTimeSet = KEGGPathwayVisual["path:hsa04662",
    ResultsFormat → "Figure", MemberSet → membersBCellReceptor, Intensities → intensitiesAll]
```

Out[160]=

{Pathway → path:hsa04662,

Results → {

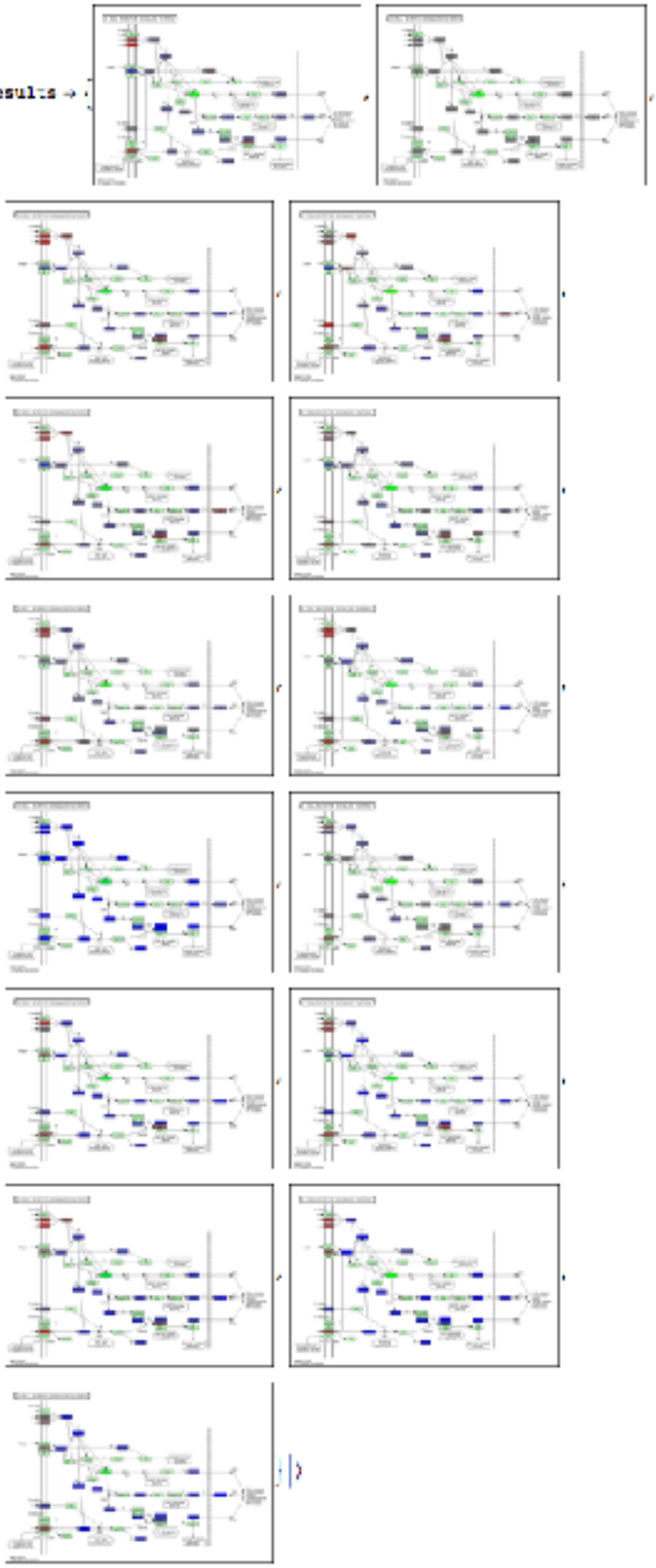

}}

We can use ListAnimate to generate a movie/animation of the results

In[82]:= `ListAnimate[exampleBCellReceptorFigureTimeSet["Results"], ImageSize → Automatic]`

Out[82]=

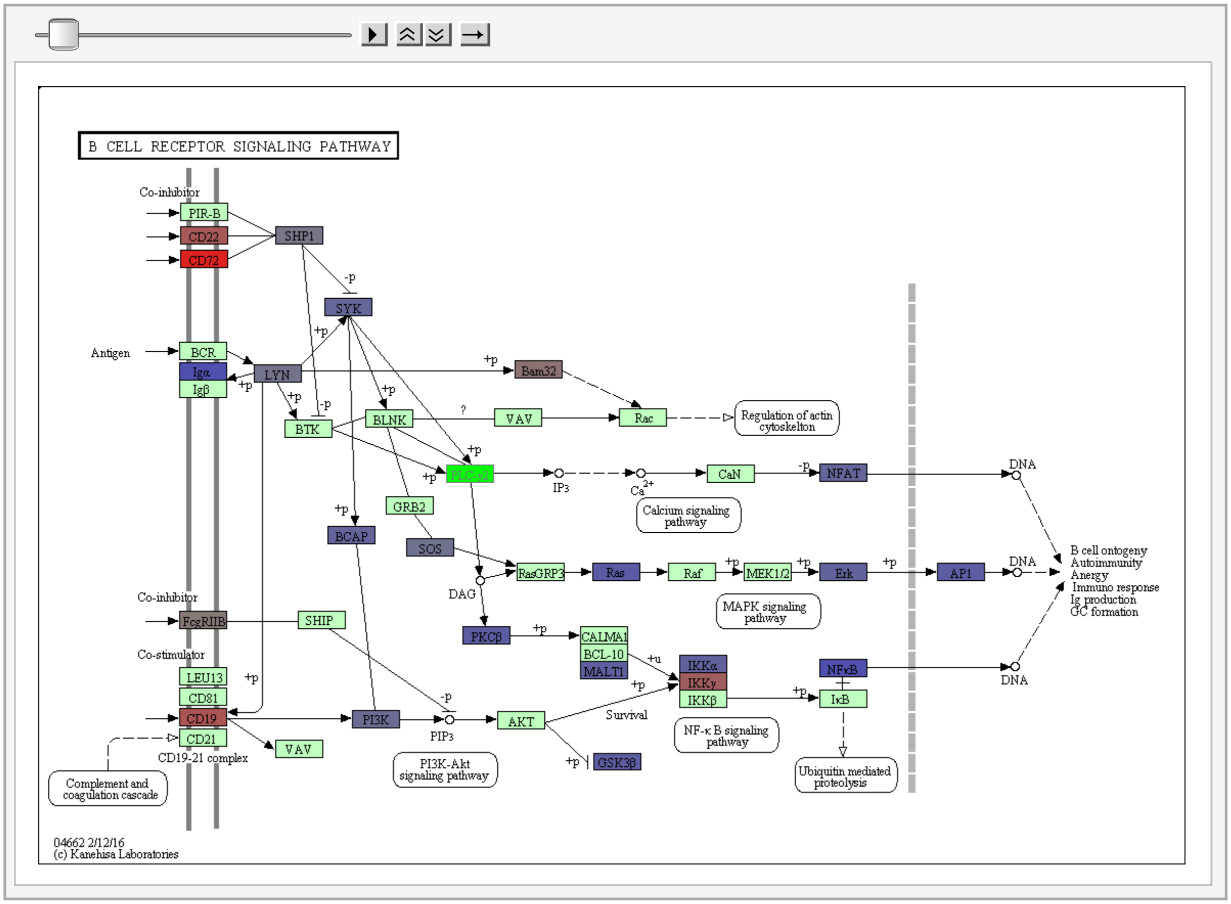

We can set the ResultsFormat to "Movie" to output a movie version:

In[161]:= `KEGGPathwayVisual["path:hsa04662", ResultsFormat → "Movie", MemberSet → membersBCellReceptor, Intensities → intensitiesAll]`

Out[161]= `<|Pathway → path:hsa04662, Results → path_hsa04662.mov|>`

Related Tutorials

- MathIOmica Dynamic Transcriptome
- MathIOmica Overview
- MathIOmica Guide
