# Supplementary material for: MathIOmica: An Integrative Platform for Dynamic Omics
Source: Sci Rep. 2016 Nov 24;6:37237. doi: 10.1038/srep37237 (PMC5121649; doi:10.1038/srep37237)
Supplement: Supplementary Note 2 [file srep37237-s2.pdf]

MathIOmica: An Integrative Platform for Dynamic Omics

George I. Mias[1,*], Tahir Yusufaly[2], Raeuf Roushangar[1], Lavida R. K. Brooks[1], Vikas V. Singh[1], and Christina Christou[3]

[1]Michigan State University, Biochemistry and Molecular Biology, East Lansing, MI 48824, USA
[2]University of Southern California, Department of Physics and Astronomy, Los Angeles, CA, 90089, USA
[3]Mercy Cancer Center, Department of Radiation Oncology, Mason City, IA 50401, USA
[*]gmias@msu.edu

SUPPLEMENTARY NOTE 2

MathIOmica: Dynamic Transcriptome

- Loading the MathIOmica Package
- Importing OmicsObject Transcriptome Data
- Processing OmicsObject Transcriptome Data
- Resampling Transcriptome Data
- Classification, Clustering and Visualization of Transcriptome Time Series
- Annotation and Enrichment
- Appendix: All Commands Up to Enrichment Analysis in One Step

The MathIOmica Dynamic Transcriptome is a brief guide to analyzing the dynamics of transcriptome data. The presentation is streamlined, without discussion of the functions used, but with links to each function provided at each step of the calculation. For more details consult MathIOmica's documentation of each funciton, and the MathIOmica Tutorial for a deeper presentation of a multiple omics analysis.

Loading the MathIOmica Package

The functions defined in the `MathIOmica`` context provide support for conducting analyses of omics data (See also the MathIOmica Overview).

This loads the package:

```
In[1]:= << MathIOmica`
```

Importing OmicsObject Transcriptome Data

We first import the transcriptomics data example (for details on how to import such data please refer to `DataImporter`, `DataImporterDirect`, `DataImporterDirectLabeled` and `OmicsObjectCreator` documentation).

We import the transcriptomics OmicsObject

```
In[1]:= rnaExample = Get[FileNameJoin[{ConstantMathIOmicaExamplesDirectory, "rnaExample"}]]

Out[1]= <|7 → <|{FAM138A, RNA} → {{0}, {OK}}, {OR4F5, RNA} → {{0}, {OK}},
    {LOC729737, RNA} → {{2.73998}, {OK}}, … 25 262 …, {LOC100507412, RNA} → {{0}, {OK}},
    {RNA45S5, RNA} → {{0}, {OK}}, {DUX4L, RNA} → {{0}, {OK}}|>,
  8 → <| … 1 … |>, … 11 …, 20 → … 1 …, 21 → <| … 1 … |> |>

large output | show less | show more | show all | set size limit...
```

There are multiple samples given by the outer associations. We can use Query to get any data. For example we can get the outer keys:

```
In[2]:= Query[Keys]@rnaExample

Out[2]= {7, 8, 9, 10, 11, 12, 13, 14, 15, 16, 17, 18, 19, 20, 21}
```

We form an association between samples to actual days of the study:

```
In[3]:= sampleToDays =
  <|"7" → "186", "8" → "255", "9" → "289", "10" → "290", "11" → "292", "12" → "294", "13" → "297", "14" → "301",
   "15" → "307", "16" → "311", "17" → "322", "18" → "329", "19" → "369", "20" → "380", "21" → "400"|>;
```

We can now do a KeyMap to rename the outer keys:

```
In[4]:= rnaLongitudinal = KeyMap[sampleToDays, rnaExample]

Out[4]= <|186 → <|{FAM138A, RNA} → {{0}, {OK}},
   {OR4F5, RNA} → {{0}, {OK}}, {LOC729737, RNA} → {{2.73998}, {OK}}, … 25 262 …,
   {LOC100507412, RNA} → {{0}, {OK}}, {RNA45S5, RNA} → {{0}, {OK}}, {DUX4L, RNA} → {{0}, {OK}}|>,
  255 → <| … 1 … |>, … 11 …, 380 → … 1 …, 400 → <| … 1 … |>|>

large output | show less | show more | show all | set size limit...
```

Processing OmicsObject Transcriptome Data

We normalize the transcriptome data using the `QuantileNormalization` function.

```
In[5]:= rnaQuantileNormed = QuantileNormalization[rnaLongitudinal]

Out[5]= <|186 → <|{FAM138A, RNA} → {{0.}, {OK}}, {OR4F5, RNA} → {{0.}, {OK}},
   {LOC729737, RNA} → {{2.73998}, {OK}}, … 25 262 …, {LOC100507412, RNA} → {{0.}, {OK}},
   {RNA45S5, RNA} → {{0.}, {OK}}, {DUX4L, RNA} → {{0.}, {OK}}|>, … 13 …, 400 → <| … 1 … |>|>

large output | show less | show more | show all | set size limit...
```

We first use `LowValueTag` to tag values of 0 as Missing[]:

```
In[6]:= rnaZeroTagged = LowValueTag[rnaQuantileNormed, 0]

Out[6]= <|186 → <|{FAM138A, RNA} → {{Missing[]}, {OK}},
   {OR4F5, RNA} → {{Missing[]}, {OK}}, {LOC729737, RNA} → {{2.73998}, {OK}}, … 25 263 …,
   {RNA45S5, RNA} → {{Missing[]}, {OK}}, {DUX4L, RNA} → {{Missing[]}, {OK}}|>,
  255 → <| … 1 … |>, … 11 …, 380 → … 1 …, 400 → <| … 1 … |>|>

large output | show less | show more | show all | set size limit...
```

We next use `LowValueTag` again to set all FPKM values <1 to unity:

```
In[7]:= rnaNoiseAdjusted = LowValueTag[rnaZeroTagged, 1, ValueReplacement → 1]

Out[7]= <|186 → <|{FAM138A, RNA} → {{Missing[]}, {OK}},
   {OR4F5, RNA} → {{Missing[]}, {OK}}, {LOC729737, RNA} → {{2.73998}, {OK}}, … 25 263 …,
   {RNA45S5, RNA} → {{Missing[]}, {OK}}, {DUX4L, RNA} → {{Missing[]}, {OK}}|>,
  255 → <| … 1 … |>, … 11 …, 380 → … 1 …, 400 → <| … 1 … |>|>

large output | show less | show more | show all | set size limit...
```

We filter out data using `FilterMissing` where the reference healhty point "255" is missing and retain data with at least 3/4 poings available:

```
In[8]:= rnaFiltered = FilterMissing[rnaNoiseAdjusted, 3/4, Reference → "255"]
```

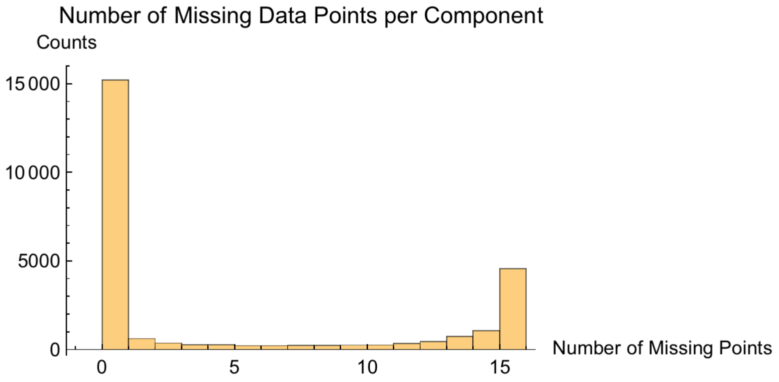


```
{Missing -> Counts: ,
 <|0 → 15 203, 1 → 615, 2 → 365, 3 → 279, 4 → 276, 5 → 217, 6 → 217, 7 → 231, 8 → 238,
  9 → 248, 10 → 255, 11 → 334, 12 → 440, 13 → 737, 14 → 1058, 15 → 4555|>}
```

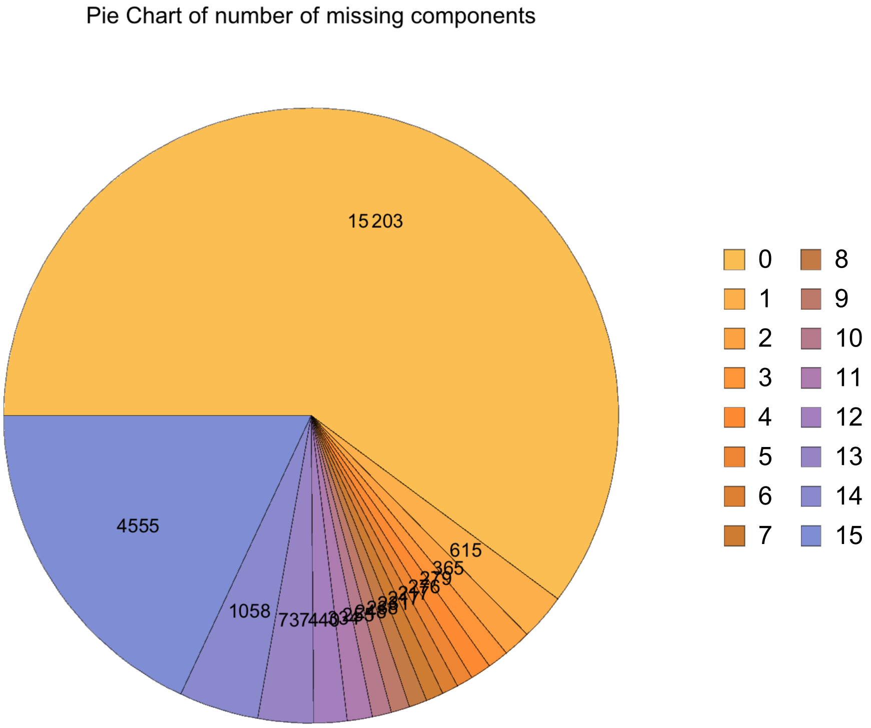


```
Out[8]= <|186 → <|{LOC729737, RNA} → {{2.73998}, {OK}},
   {DDX11L1, RNA} → {{6.75461}, {OK}}, {WASH7P, RNA} → {{11.8883}, {OK}},
   ⋯ 16 457 ⋯, {KDM5D, RNA} → {{7.73125}, {OK}}, {UTY, RNA} → {{3.16532}, {OK}}|>,
  255 → <| ⋯ 1 ⋯ |>, ⋯ 11 ⋯, 380 → <| ⋯ 1 ⋯ |>, 400 → <| ⋯ 1 ⋯ |>|>

large output    show less    show more    show all    set size limit...
```

We extract the times for the filtered RNA data using `TimeExtractor`:

In[9]:=
```
timesRNA = TimeExtractor[rnaFiltered]
```

Out[9]=
```
{186, 255, 289, 290, 292, 294, 297, 301, 307, 311, 322, 329, 369, 380, 400}
```

For each gene we now extract a time series (list of values) corresponding to these times using `CreateTimeSeries`:

In[10]:=
```
timeSeriesRNA = CreateTimeSeries[rnaFiltered]
```

Out[10]=
```
<|{LOC729737, RNA} → {2.73998, 1, 5.15563, 4.53362,
   5.71829, 1, 1.413, 2.38838, 1, 1, 2.2049, 2.18935, 4.05165, 2.70102, 1.22675},
 ··· 16460 ···, {UTY, RNA} → {3.16532, 3.28427, 3.06644, 1.77757, 2.90109, 2.48543,
   2.49979, 2.65107, 1, 2.24661, 1.49351, 1.56608, 1.59413, 1.13702, 1}|>
```

large output | show less | show more | show all | set size limit...

We use `SeriesApplier` to implement a logarithm transformation:

In[11]:=
```
timeSeriesRNALog = SeriesApplier[Log, timeSeriesRNA]
```

Out[11]=
```
<|{LOC729737, RNA} → {1.00795, 0, 1.64009, 1.51152, 1.74367, 0, 0.345715, 0.870615,
   0, 0, 0.790682, 0.783605, 1.39912, 0.993629, 0.204368}, ··· 16460 ···, ··· 1 ··· → ··· 1 ···|>
```

large output | show less | show more | show all | set size limit...

We compare every value in each series to the healthy "255" time point, which is the second element in each series. We use `SeriesInternalCompare`:

In[12]:=
```
rnaCompared = SeriesInternalCompare[timeSeriesRNALog, ComparisonIndex → 2]
```

Out[12]=
```
<|{LOC729737, RNA} → {1.00795, 0, 1.64009, 1.51152, 1.74367, 0, 0.345715, 0.870615,
   0, 0, 0.790682, 0.783605, 1.39912, 0.993629, 0.204368}, ··· 16378 ···, ··· 1 ··· → ··· 1 ···|>
```

large output | show less | show more | show all | set size limit...

Next, we normalize each series, using again `SeriesApplier`:

In[13]:=
```
normedRNACompared = SeriesApplier[Normalize, rnaCompared]
```

Out[13]=
```
<|{LOC729737, RNA} → {0.268104, 0., 0.436246, 0.402048, 0.463797, 0.,
   0.0919564, 0.231574, 0., 0., 0.210313, 0.20843, 0.372152, 0.264294, 0.0543597},
 ··· 16378 ···, {UTY, RNA} → {-0.0145984, 0., -0.0271576, -0.242935, -0.049093, -0.110288,
   ··· 3 ···, -0.150266, -0.311839, -0.293063, -0.286038, -0.41976, -0.470576}|>
```

large output | show less | show more | show all | set size limit...

Finally, we use `ConstantSeriesClean` to remove constant series, as we are interested in changing time patterns:

```
In[14]:= rnaFinalTimeSeries = ConstantSeriesClean[normedRNACompared]
```

```
Removed series and returning filtered
  list. If you would like a list of removed keys run the
  command ConstantSeriesClean[data,ReturnDropped → True].
```

```
Out[14]= <|{LOC729737, RNA} → {0.268104, 0., 0.436246, 0.402048, 0.463797, 0.,
   0.0919564, 0.231574, 0., 0., 0.210313, 0.20843, 0.372152, 0.264294, 0.0543597},
   ⋯ 11628 ⋯, {UTY, RNA} → {-0.0145984, 0., -0.0271576, -0.242935, -0.049093, -0.110288,
   ⋯ 3 ⋯, -0.150266, -0.311839, -0.293063, -0.286038, -0.41976, -0.470576}|>

large output | show less | show more | show all | set size limit...
```

Resampling Transcriptome Data

In addition to the above, we want to create a resampled distribution for the transcriptome dataset prior to classification and clustering. We repeat the steps in the processing section above using a resampled set of measurements.

We create a resampling of 100000 sets using `BootstrapGeneral`:

```
In[15]:= rnaBootstrap = BootstrapGeneral[rnaLongitudinal, 100 000]
```

```
Out[15]= <|186 → <|1 → {{8.0501}, {OK}}, 2 → {{0}, {OK}}, 3 → {{0.153937}, {OK}}, 4 → {{0.0190801}, {OK}},
   5 → {{0}, {OK}}, ⋯ 99 991 ⋯, 99 997 → {{0.0798867}, {OK}}, 99 998 → {{3.78551}, {OK}},
   99 999 → {{2.0606}, {OK}}, 100 000 → {{0}, {OK}}|>, 255 → <| ⋯ 1 ⋯ |>, ⋯ 12 ⋯, 400 → <| ⋯ 1 ⋯ |>|>

large output | show less | show more | show all | set size limit...
```

As with the regular data we: 1. normalize , 2. tag zero values, 3. tag values of FPKM <1, 4. filter missing data, 5. create a time series, 6. take a logarithm, 7. compare to "255" reference, 8. take the norm of each time series, 9. clean out constant series

```
In[16]:= (*1*)rnaBootstrapQuantileNormed = QuantileNormalization[rnaBootstrap];
(*2*)rnaBootstrapZeroTagged = LowValueTag[rnaBootstrapQuantileNormed, 0];
(*3*) rnaBootstrapNoiseAdjusted = LowValueTag[rnaBootstrapZeroTagged, 1, ValueReplacement → 1];
(*4*) rnaBootstrapFiltered = FilterMissing[rnaBootstrapNoiseAdjusted, 3/4, Reference → "255"];
(*5*) timeSeriesBootstrapRNA = CreateTimeSeries[rnaBootstrapFiltered];
(*6*) timeSeriesBootstrapRNALog = SeriesApplier[Log, timeSeriesBootstrapRNA];
(*7*)rnaBootstrapCompared = SeriesInternalCompare[timeSeriesBootstrapRNALog, ComparisonIndex → 2];
(*8*)normedBootstrapRNACompared = SeriesApplier[Normalize, rnaBootstrapCompared];
(*9*)rnaBootstrapFinalTimeSeries = ConstantSeriesClean[normedBootstrapRNACompared];
```

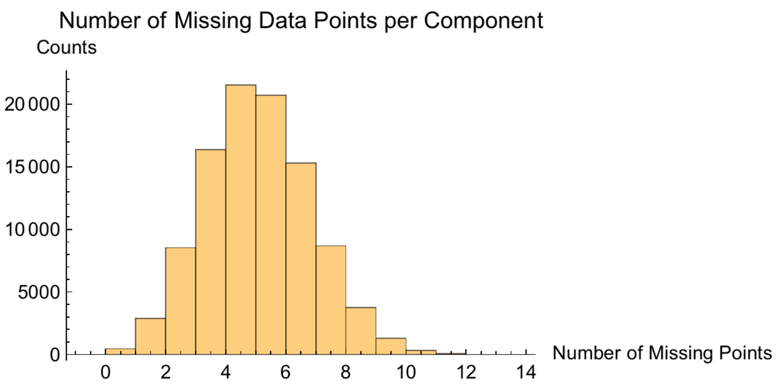


```
{Missing -> Counts: ,
 <|0 → 462, 1 → 2900, 2 → 8539, 3 → 16 384, 4 → 21 533, 5 → 20 724, 6 → 15 314,
  7 → 8686, 8 → 3752, 9 → 1305, 10 → 323, 11 → 72, 12 → 5, 13 → 1|>}
```

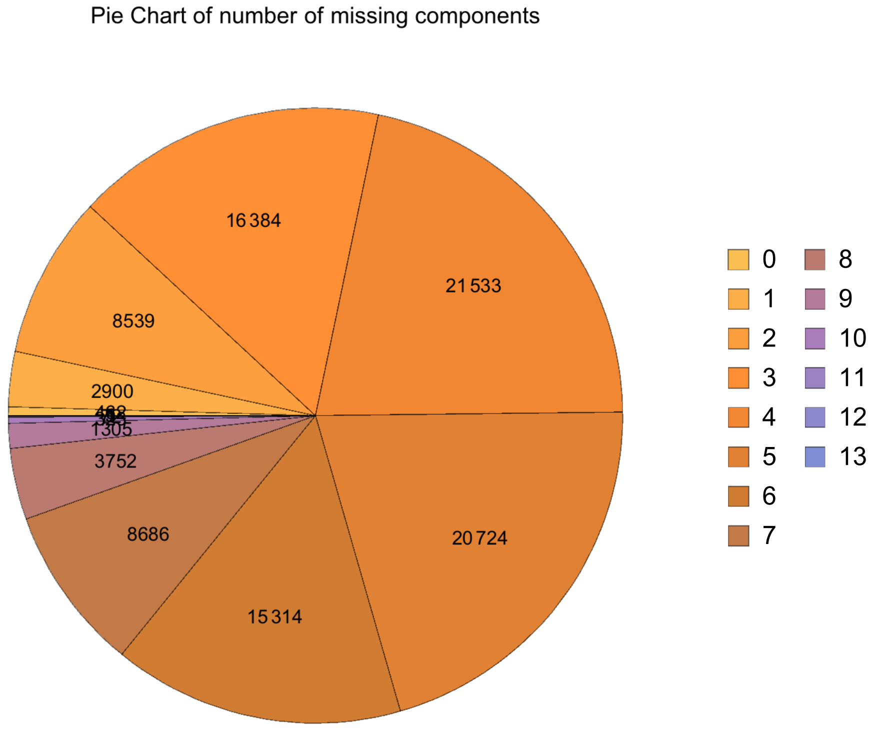

Classification, Clustering and Visualization of Transcriptome Time Series

In this section we will classify the transcriptome time series based on patterns in the series. For the classification we will use `TimeSeriesClassification`.

Before we classify our transcriptome data, we estimate for the "LombScargle" Method a 0.95 quantile cutoff from the bootstrap transcriptome data using `QuantileEstimator` :

In[25]:=
```
q95RNA = QuantileEstimator[rnaBootstrapFinalTimeSeries, timesRNA]
```

Out[25]=
```
0.85761
```

Next, we estimate the "Spikes" 0.95 quantile cutoff from the bootstrap transcriptome data:

In[26]:=
```
q95RNASpikes = QuantileEstimator[rnaBootstrapFinalTimeSeries, timesRNA, Method → "Spikes"]
```

Out[26]=
```
<|12 → {0.815473, -0.443516}, 13 → {0.803768, -0.422306},
 14 → {0.787953, -0.402833}, 15 → {0.769828, -0.388446}|>
```

Now we can classify the transcriptome time series data based on these cutoffs using `TimeSeriesClassification` :

In[27]:=
```
rnaClassification = TimeSeriesClassification[rnaFinalTimeSeries,
  timesRNA, LombScargleCutoff → q95RNA, SpikeCutoffs → q95RNASpikes]
```

```
Method → "LombScargle"
```

Out[27]=
```
<|SpikeMax → <|⋯ 1 ⋯|>, ⋯ 7 ⋯,
 f7 → <|{MIR6723, RNA} → {{0.123496, 0.0627112, 0.00126796, 0.0704294, 0.162169, 0.401532, 0.887878},
     {⋯ 1 ⋯}}, ⋯ 55 ⋯, {CLIC2, RNA} → {⋯ 1 ⋯}|>|>
```

large output | show less | show more | show all | set size limit...

To obtain the possible frequencies we simply run `LombScargle` over the desired times for one of the time series and set the `FrequenciesOnly` option to `True` :

In[28]:=
```
LombScargle[rnaFinalTimeSeries[[1]], timesRNA, FrequenciesOnly → True]
```

Out[28]=
```
<|f1 → 0.00500668, f2 → 0.0104306, f3 → 0.0158545,
 f4 → 0.0212784, f5 → 0.0267023, f6 → 0.0321262, f7 → 0.0375501|>
```

We now cluster our RNA data using `TimeSeriesClusters` :

In[29]:=
```
rnaClusters = TimeSeriesClusters[rnaClassification, PrintDendrograms → True]
```

Agglomerate::ties : 226 ties have been detected; reordering input may produce a different result. ≫

Agglomerate::ties : 1 ties have been detected; reordering input may produce a different result. ≫

Agglomerate::ties : 1 ties have been detected; reordering input may produce a different result. ≫

General::stop : Further output of Agglomerate::ties will be suppressed during this calculation. ≫

<|SpikeMax →

<|G1 → <|{G1S1} → 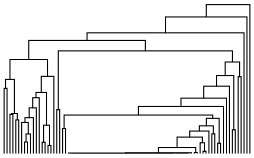, {G1S2} → 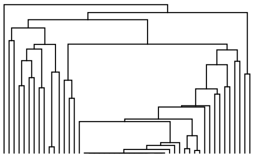, {G1S3} →

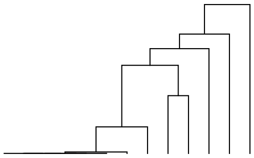, {G1S4} → 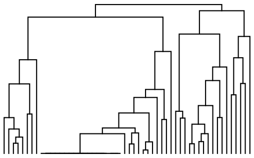, {G1S5} → 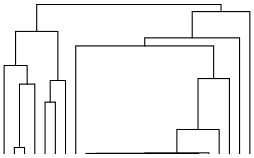,

{G1S6} → 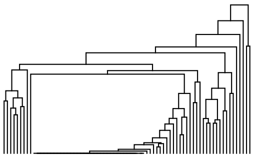, {G1S7} → 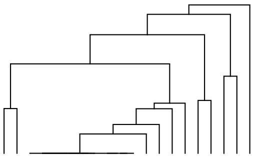,

{G1S8} → 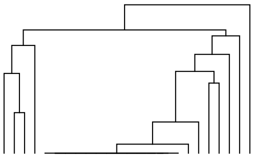, {G1S9} → 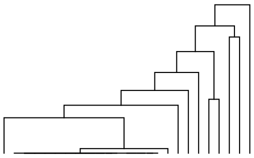,

{G1S10} → 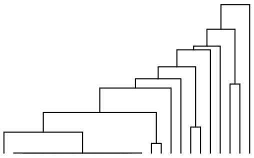, {G1S11} → 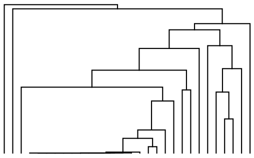,

{G1S12} → 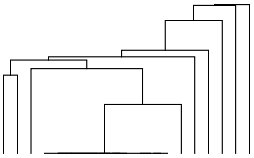, {G1S13} → 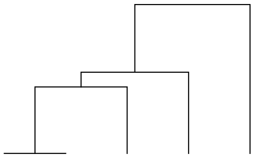,

{G1S14} → 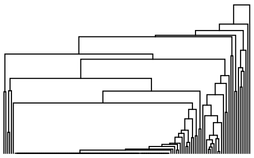 |⟩, G2 → ⟨| {G2S1} → 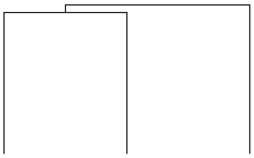,

{G2S2} → {{-0.152057, 0., 0.23905, -0.0174597, 0.234592, -0.192606, 0.147406, 0.116446, 0.779354, -0.109448, 0.0185872, 0.198649, 0.074635, 0.247257, -0.257149} → {RRN3P3, RNA}} |⟩ |⟩,

SpikeMin → ⟨| G1 → ⟨| {G1S1} → 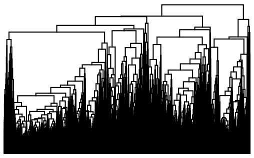, {G1S2} → 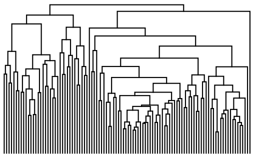 |⟩ |⟩,

f1 →

⟨| G1 → ⟨| {G1S1} → 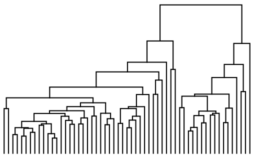, {G1S2} → 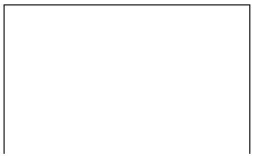 |⟩,

G2 → ⟨| {G2S1} → {{0.129049, 0., 0.138131, 0.184863, 0.177164, 0.195737, 0.176713, 0.169323, 0.388209, 0.138266, 0.221229, 0.381703, 0.407244, 0.375505, 0.359416} → {C1orf35, RNA}}, {G2S2} → 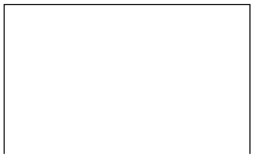 |⟩ |⟩,

f2 → ⟨| G1 → ⟨| {G1S1} → 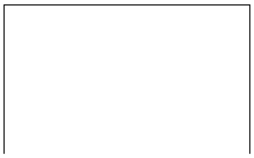 |⟩,

G2 → <| {G2S1} → {{0.30694, 0., 0.216277, 0.3638, 0.212436, 0.331803, 0.290702, 0.219389, 0.235242, 0.340605, 0.132122, 0.20408, 0.0814162, 0.280708, 0.350598} → {SNRPG, RNA}} |> |⟩,

f3 → ⟨| G1 → ⟨| {G1S1} → 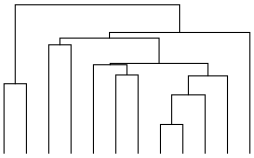 , {G1S2} →

{{0., 0., 0.192522, 0.266594, 0.174533, 0.110734, 0., 0.17435, 0., 0., 0., 0.230308, 0., 0.283945, 0.827692} → {TUBB2A, RNA}} |⟩,

G2 → <| {G2S1} → {{-0.0367349, 0., 0.353367, 0.278445, 0.169023, 0.462754, 0.138503, 0.172756, 0.0845424, 0.182143, 0.0129593, 0.307028, 0.305215, 0.13458, 0.508416} → {HIST1H4C, RNA}} |> |⟩,

f4 → ⟨| G1 → ⟨| {G1S1} → 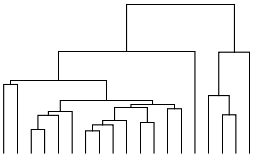 , {G1S2} → 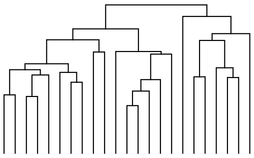 |⟩ |⟩,

f5 →

⟨| G1 → ⟨| {G1S1} → 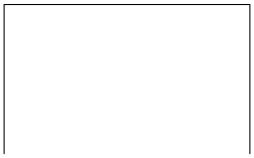 , {G1S2} → 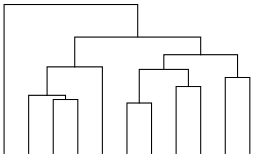 |⟩,

G2 → <| {G2S1} → {{-0.217795, 0., 0.103979, -0.232642, 0.122186, 0.0302431, -0.255409, 0.0881042, -0.65238, -0.152971, 0.232259, -0.08014, 0.1381, -0.515318, 0.0692877} → {FBXL8, RNA}} |> |⟩,

f6 → ⟨| G1 → ⟨| {G1S1} → 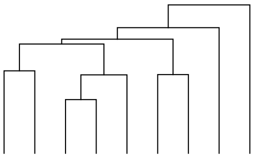 , {G1S2} →

{{-0.0903641, 0., 0.0987218, -0.529464, -0.0542017, 0.214781, -0.220934, -0.0393788, -0.546495, 0.131911, 0.041652, -0.023886, -0.356358, -0.162879, -0.361167} → {CKMT2-AS1, RNA}} |⟩ |⟩,

f7 → ⟨| G1 → ⟨| {G1S1} → 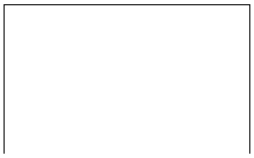 , {G1S2} → 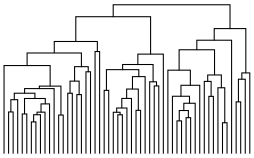 |⟩ |⟩ |⟩

Out[29]=
```
<|SpikeMax → <|Cluster → Cluster[Cluster[Cluster[Cluster[ ⋯1⋯ ], Cluster[ ⋯1⋯ ], 0.804523, 408, 158],
    Cluster[ ⋯1⋯ ], 0.865475, 566, 18], ⋯1⋯ , 0.903333, 584, 4],
  ⋯4⋯ , GroupAssociations → <| ⋯1⋯ |>|>, ⋯7⋯ , f7 → ⋯1⋯ |>
```

large output | show less | show more | show all | set size limit...

For each class we can generate a dendrogram/heatmap plot using `TimeSeriesDendrogramsHeatmaps` , with groupings represented on the left, and highlighted to represent the grouping level. The G, S, columns represent the groupings and subgroupings generated by the clustering. The legend shows the corresponding groupings and subgrouping, and the number of elements in each group subgroup.

In[30]:=
```
TimeSeriesDendrogramsHeatmaps[rnaClusters]
```

SpikeMax

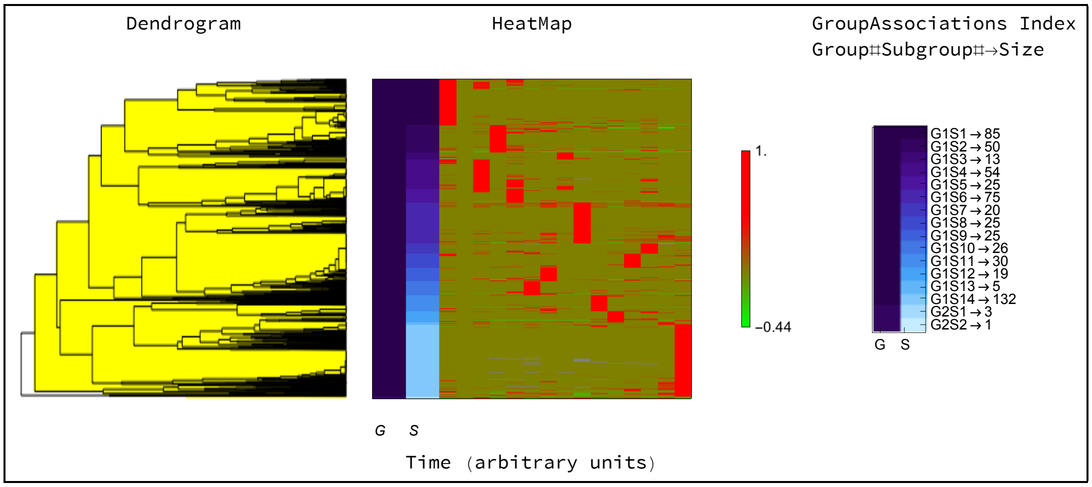

SpikeMin

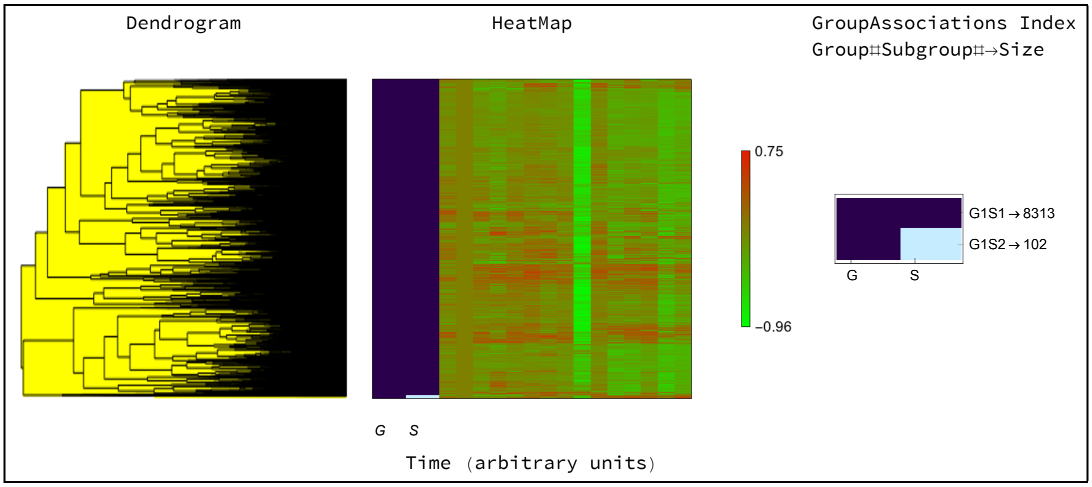

f1

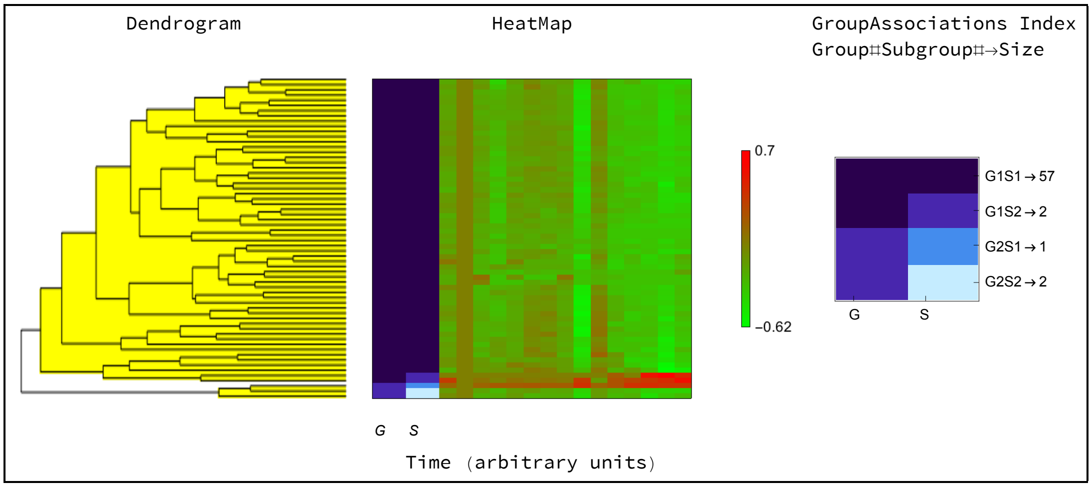

f2

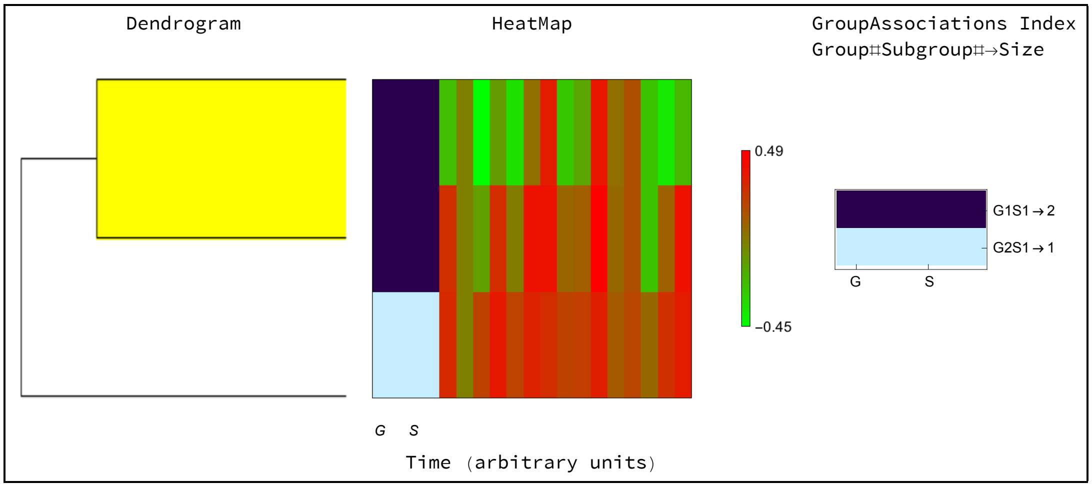

Out[30]=

f3

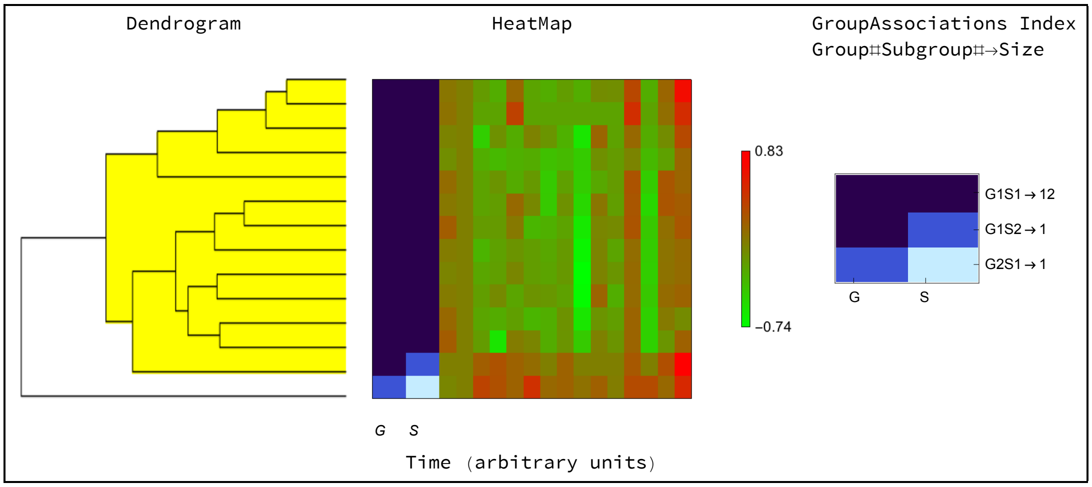

f4

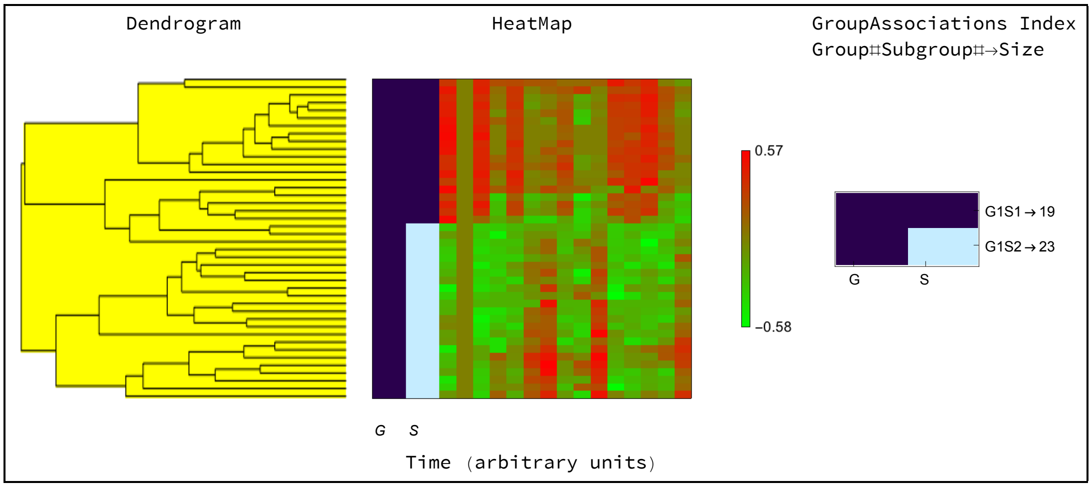
Dendrogram
HeatMap
GroupAssociations Index
Group♯Subgroup♯→Size
0.57
−0.58
G1S1→19
G1S2→23
G
S
G S
Time (arbitrary units)

f5

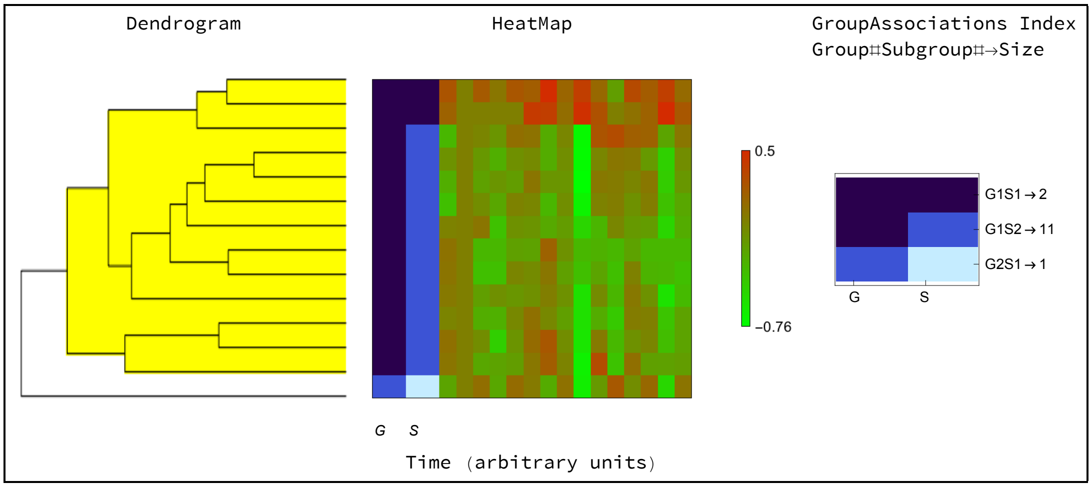
Dendrogram
HeatMap
GroupAssociations Index
Group♯Subgroup♯→Size
0.5
−0.76
G1S1→2
G1S2→11
G2S1→1
G
S
G S
Time (arbitrary units)

f6

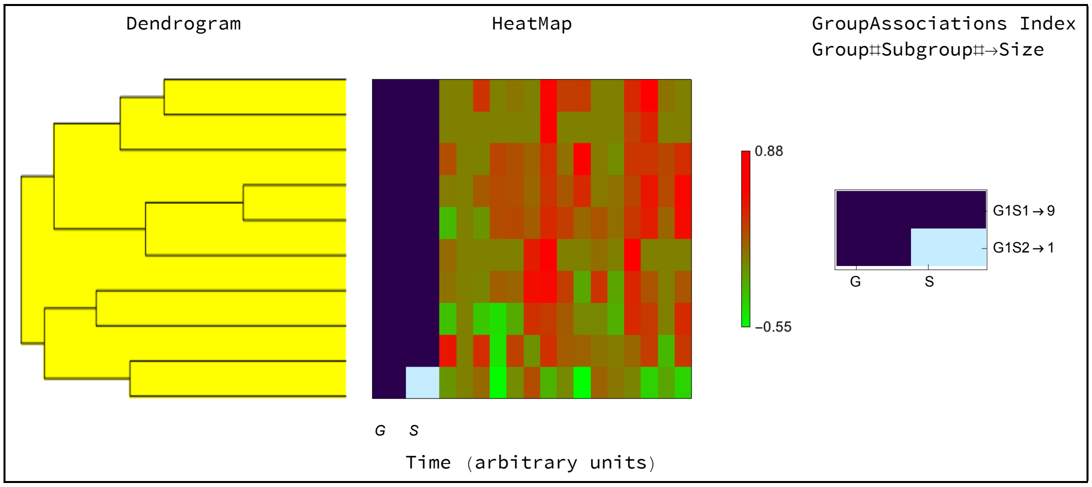
Dendrogram
HeatMap
GroupAssociations Index
Group♯Subgroup♯→Size
0.88
−0.55
G1S1→9
G1S2→1
G
S
G S
Time (arbitrary units)

f7

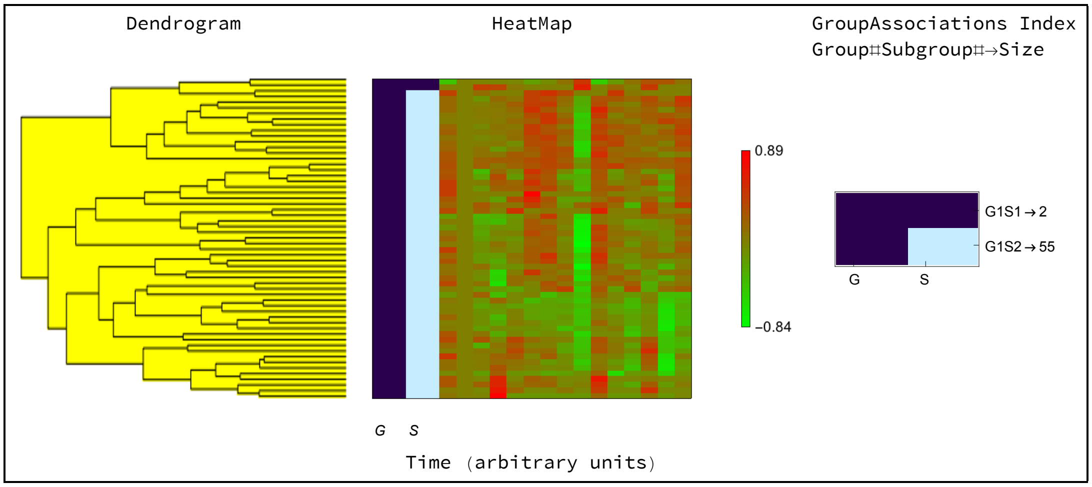

Annotation and Enrichment

We can carry out Gene Ontology analysis using `GOAnalysis` for all the classes and groups/subgroups. We only report terms for which there are at least 3 members (2 sets of GO terms, one each for proteomics and transcriptomics). Please note that this may be a time consuming computation.

In[31]:=
```
goAnalysisRNA = GOAnalysis[rnaClusters, OntologyLengthFilter → 3, ReportFilter → 3 ];
```

The output of `GOAnalysis` has enrichments for each class and group

In[32]:=
```
Query[Keys]@goAnalysisRNA
```

Out[32]=
```
{SpikeMax, SpikeMin, f1, f2, f3, f4, f5, f6, f7}
```

In[33]:=
```
Query[All, Keys]@goAnalysisRNA
```

Out[33]=
```
⟨|SpikeMax →
  {G1S1, G1S2, G1S3, G1S4, G1S5, G1S6, G1S7, G1S8, G1S9, G1S10, G1S11, G1S12, G1S13, G1S14, G2S1, G2S2},
 SpikeMin → {G1S1, G1S2}, f1 → {G1S1, G1S2, G2S1, G2S2}, f2 → {G1S1, G2S1}, f3 → {G1S1, G1S2, G2S1},
 f4 → {G1S1, G1S2}, f5 → {G1S1, G1S2, G2S1}, f6 → {G1S1, G1S2}, f7 → {G1S1, G1S2}|⟩
```

We can view results for any of the groups (and also check out the behavior using the heatmaps generated in the previous section

In[34]:= **Query["SpikeMax", "G1S1"]@goAnalysisRNA**

```
Out[34]= ⟨|GO:0006351 → {{2.59263×10^-10, 9.48904×10^-8, True},
    {60, 2285, 47241, 18}, {{transcription, DNA-templated, biological_process},
     {{{ZNF234, RNA}}, {{TP53INP2, RNA}}, {{ZNF841, RNA}}, {{SCML1, RNA}}, {{ZNF514, RNA}}, {{ZNF169, RNA}},
      {{HOXC4, RNA}}, {{ZBTB26, RNA}}, {{ZNF532, RNA}}, {{ZNF823, RNA}}, {{ZNF441, RNA}}, {{ZNF440, RNA}},
      {{ZSCAN22, RNA}}, {{ZNF577, RNA}}, {{TBX19, RNA}}, {{ZNF2, RNA}}, {{ZNF528, RNA}}, {{ZNF436, RNA}}}}},
 GO:0003677 → {{2.39558×10^-8, 4.3839×10^-6, True}, {60, 2344, 47241, 16},
    {{DNA binding, molecular_function}, {{{ZNF234, RNA}}, {{ZNF841, RNA}}, {{SCML1, RNA}}, {{ZNF514, RNA}},
      {{ZNF169, RNA}}, {{E2F2, RNA}}, {{ZBTB26, RNA}}, {{ZNF823, RNA}}, {{ZNF441, RNA}}, {{ZNF440, RNA}},
      {{DNASE1L3, RNA}}, {{ZNF577, RNA}}, {{ZNF2, RNA}}, {{ZNF528, RNA}}, {{BLM, RNA}}, {{ZNF436, RNA}}}}},
 GO:0003700 → {{1.01591×10^-7, 0.0000113597, True}, {60, 1623, 47241, 13},
    {{transcription factor activity, sequence-specific DNA binding, molecular_function}, {{{ZNF234, RNA}},
      {{ZNF841, RNA}}, {{SCML1, RNA}}, {{ZNF514, RNA}}, {{E2F2, RNA}}, {{HOXC4, RNA}}, {{ZNF532, RNA}},
      {{ZSCAN22, RNA}}, {{ZNF577, RNA}}, {{TBX19, RNA}}, {{ZNF2, RNA}}, {{ZNF528, RNA}}, {{ZNF436, RNA}}}}},
 GO:0046872 → {{1.2415×10^-7, 0.0000113597, True}, {60, 3010, 47241, 17},
    {{metal ion binding, molecular_function},
     {{{ZNF234, RNA}}, {{ZNF841, RNA}}, {{POLI, RNA}}, {{ZNF514, RNA}}, {{ZNF169, RNA}}, {{B3GAT1, RNA}},
      {{ADHFE1, RNA}}, {{ZBTB26, RNA}}, {{ZNF532, RNA}}, {{ZNF823, RNA}}, {{ZNF441, RNA}}, {{ZNF440, RNA}},
      {{ZSCAN22, RNA}}, {{ENPP5, RNA}}, {{ZNF577, RNA}}, {{ZNF528, RNA}}, {{ZNF436, RNA}}}}},
 GO:0006355 → {{4.7938×10^-7, 0.0000350906, True}, {60, 3311, 47241, 17},
    {{regulation of transcription, DNA-templated, biological_process},
     {{{ZNF234, RNA}}, {{ZNF841, RNA}}, {{SCML1, RNA}}, {{ZNF514, RNA}}, {{ZNF169, RNA}}, {{E2F2, RNA}},
      {{HOXC4, RNA}}, {{ZBTB26, RNA}}, {{ZNF532, RNA}}, {{ZNF823, RNA}}, {{ZNF441, RNA}}, {{ZNF440, RNA}},
      {{ZSCAN22, RNA}}, {{ZNF577, RNA}}, {{ZNF2, RNA}}, {{ZNF528, RNA}}, {{ZNF436, RNA}}}}},
 GO:0005634 → {{7.63375×10^-7, 0.0000465659, True}, {60, 7197, 47241, 25},
    {{nucleus, cellular_component}, {{{ZNF234, RNA}}, {{SEPSECS, RNA}}, {{TP53INP2, RNA}},
      {{ZNF841, RNA}}, {{SCML1, RNA}}, {{POLI, RNA}}, {{ZNF514, RNA}}, {{ZNF169, RNA}}, {{FANCD2, RNA}},
      {{HOXC4, RNA}}, {{ZBTB26, RNA}}, {{ZNF532, RNA}}, {{ZNF823, RNA}}, {{ZNF441, RNA}},
      {{ZNF440, RNA}}, {{STK36, RNA}}, {{DNASE1L3, RNA}}, {{OCRL, RNA}}, {{PPAN-P2RY11, RNA}},
      {{ZSCAN22, RNA}}, {{ZNF577, RNA}}, {{TBX19, RNA}}, {{ZNF2, RNA}}, {{ZNF528, RNA}}, {{BLM, RNA}}}}},
 GO:0005515 → {{9.17899×10^-6, 0.00047993, True}, {60, 8801, 47241, 26},
    {{protein binding, molecular_function}, {{{SEPSECS, RNA}}, {{TP53INP2, RNA}}, {{POLI, RNA}},
      {{ZNF169, RNA}}, {{FANCD2, RNA}}, {{E2F2, RNA}}, {{HOXC4, RNA}}, {{DAPK2, RNA}}, {{SLC22A5, RNA}},
      {{CEP152, RNA}}, {{ZBTB26, RNA}}, {{KLHL3, RNA}}, {{ZNF440, RNA}}, {{NLRP2, RNA}}, {{STK36, RNA}},
      {{C1QTNF6, RNA}}, {{OCRL, RNA}}, {{DLG5, RNA}}, {{ZSCAN22, RNA}}, {{AGPAT4, RNA}}, {{ITIH4, RNA}},
      {{ZNF2, RNA}}, {{BLM, RNA}}, {{ELMO3, RNA}}, {{ZNF436, RNA}}, {{PACSIN1, RNA}}}}},
 GO:0042384 → {{0.0000447897, 0.00204913, True}, {60, 153, 47241, 4}, {{cilium assembly, biological_process},
     {{{IFT172, RNA}}, {{WDR19, RNA}}, {{STK36, RNA}}, {{OCRL, RNA}}}}}|⟩
```

We can export the reports, for example to the `$UserDocumentDirectory`:

In[35]:= **EnrichmentReportExport[goAnalysisRNA,**
OutputDirectory → $UserDocumentsDirectory, AppendString → "GOAnalysisRNA"];

We carry out our KEGG: Kyoto Encyclopedia of Genes and Genomes pathway analysis using `KEGGAnalysis` for all the classes and groups/subgroups. We only report terms for which there are at least 2 members. Please note that this is a time consuming computation.

In[36]:= **keggAnalysisRNA = KEGGAnalysis[rnaClusters, ReportFilter → 2];**

The output of `KEGGAnalysis` has enrichments for each class and group

In[37]:= **Query[Keys]@keggAnalysisRNA**

Out[37]= {SpikeMax, SpikeMin, f1, f2, f3, f4, f5, f6, f7}

In[38]:= **Query[All, Keys]@keggAnalysisRNA**

```
Out[38]= ⟨|SpikeMax →
  {G1S1, G1S2, G1S3, G1S4, G1S5, G1S6, G1S7, G1S8, G1S9, G1S10, G1S11, G1S12, G1S13, G1S14, G2S1, G2S2},
 SpikeMin → {G1S1, G1S2}, f1 → {G1S1, G1S2, G2S1, G2S2}, f2 → {G1S1, G2S1}, f3 → {G1S1, G1S2, G2S1},
 f4 → {G1S1, G1S2}, f5 → {G1S1, G1S2, G2S1}, f6 → {G1S1, G1S2}, f7 → {G1S1, G1S2}|⟩
```

We can export the reports, for example to the `$UserDocumentDirectory`:

```
In[39]:= EnrichmentReportExport[keggAnalysisRNA,
 OutputDirectory → $UserDocumentsDirectory, AppendString → "KEGGAnalysisRNA"]
```

We can view results for any of the groups (and also check out the behavior using the heatmaps generated in the previous section

```
In[44]:= Query["SpikeMax", "G1S2"]@keggAnalysisRNA

Out[44]= ⟨|path:hsa05142 → {{0.000789315, 0.0370978, True},
   {13, 104, 7086, 3}, {Chagas disease (American trypanosomiasis) - Homo sapiens (human),
    {{{C1QB, RNA}}, {{CCL3L3, RNA}}, {{CCL2, RNA}}}}}|⟩
```

```
In[46]:= Query["SpikeMin", "G1S1"]@keggAnalysisRNA

Out[46]= ⟨|path:hsa01100 → {{2.31644×10^-39, 6.94931×10^-37, True},
   {2480, 1243, 7086, 639}, {Metabolic pathways - Homo sapiens (human),
    {{{FAH, RNA}}, {{ST3GAL3, RNA}}, {{ODC1, RNA}}, {{DHCR7, RNA}}, {{NDUFS5, RNA}}, {{NDUFA4, RNA}},
     {{NDUFA7, RNA}}, {{DHCR24, RNA}}, {{HSD17B12, RNA}}, {{DCK, RNA}}, {{SUCLG2, RNA}}, ⋯ 618 ⋯,
     {{IPMK, RNA}}, {{LPCAT2, RNA}}, {{ATP6V1A, RNA}}, {{ACSL4, RNA}}, {{PAPSS1, RNA}}, {{ALDH1A1, RNA}},
     {{PLA2G7, RNA}}, {{IDH1, RNA}}, {{ATP6V0B, RNA}}, {{AFMID, RNA}}}}}, ⋯ 153 ⋯, … → ⋯ 1 ⋯|⟩

large output | show less | show more | show all | set size limit...
```

```
In[56]:= nfkbPathwayRNAExample = Query["SpikeMin", "G1S1", {30}]@keggAnalysisRNA

Out[56]= ⟨|path:hsa04064 → {{3.94357×10^-10, 3.94357×10^-9, True},
   {2480, 93, 7086, 62}, {NF-kappa B signaling pathway - Homo sapiens (human),
    {{{BCL2L1, RNA}}, {{CD40LG, RNA}}, {{PRKCQ, RNA}}, {{PARP1, RNA}}, {{MALT1, RNA}},
     {{TAB2, RNA}}, {{CSNK2A2, RNA}}, {{TAB3, RNA}}, {{RIPK1, RNA}}, {{TIRAP, RNA}}, {{TRAF3, RNA}},
     {{IRAK1, RNA}}, {{DDX58, RNA}}, {{CSNK2A1, RNA}}, {{CHUK, RNA}}, {{PRKCB, RNA}}, {{BTK, RNA}},
     {{CARD11, RNA}}, {{RELA, RNA}}, {{BIRC2, RNA}}, {{IRAK4, RNA}}, {{ATM, RNA}}, {{PLCG1, RNA}},
     {{TAB1, RNA}}, {{PLCG2, RNA}}, {{IKBKB, RNA}}, {{MAP3K14, RNA}}, {{TRIM25, RNA}}, {{LCK, RNA}},
     {{TNFAIP3, RNA}}, {{TICAM1, RNA}}, {{TRAF5, RNA}}, {{ZAP70, RNA}}, {{TRAF1, RNA}},
     {{TNFRSF1A, RNA}}, {{CCL4, RNA}}, {{IKBKG, RNA}}, {{TRAF2, RNA}}, {{TRADD, RNA}},
     {{CD40, RNA}}, {{RELB, RNA}}, {{BCL2, RNA}}, {{PIAS4, RNA}}, {{LAT, RNA}}, {{TNFRSF13C, RNA}},
     {{NFKB2, RNA}}, {{BLNK, RNA}}, {{TLR4, RNA}}, {{MAP3K7, RNA}}, {{NFKB1, RNA}}, {{TRAF6, RNA}},
     {{ICAM1, RNA}}, {{CFLAR, RNA}}, {{SYK, RNA}}, {{MYD88, RNA}}, {{LYN, RNA}}, {{NFKBIA, RNA}},
     {{IL1B, RNA}}, {{LTBR, RNA}}, {{CD14, RNA}}, {{TICAM2, RNA}}, {{LY96, RNA}}}}}|⟩
```

```
In[57]:= pathwaymembers = Query["SpikeMin", "G1S1", 30, 3, 2, All, 1]@keggAnalysisRNA

Out[57]= {{BCL2L1, RNA}, {CD40LG, RNA}, {PRKCQ, RNA}, {PARP1, RNA}, {MALT1, RNA}, {TAB2, RNA},
 {CSNK2A2, RNA}, {TAB3, RNA}, {RIPK1, RNA}, {TIRAP, RNA}, {TRAF3, RNA}, {IRAK1, RNA}, {DDX58, RNA},
 {CSNK2A1, RNA}, {CHUK, RNA}, {PRKCB, RNA}, {BTK, RNA}, {CARD11, RNA}, {RELA, RNA}, {BIRC2, RNA},
 {IRAK4, RNA}, {ATM, RNA}, {PLCG1, RNA}, {TAB1, RNA}, {PLCG2, RNA}, {IKBKB, RNA}, {MAP3K14, RNA},
 {TRIM25, RNA}, {LCK, RNA}, {TNFAIP3, RNA}, {TICAM1, RNA}, {TRAF5, RNA}, {ZAP70, RNA}, {TRAF1, RNA},
 {TNFRSF1A, RNA}, {CCL4, RNA}, {IKBKG, RNA}, {TRAF2, RNA}, {TRADD, RNA}, {CD40, RNA}, {RELB, RNA},
 {BCL2, RNA}, {PIAS4, RNA}, {LAT, RNA}, {TNFRSF13C, RNA}, {NFKB2, RNA}, {BLNK, RNA}, {TLR4, RNA},
 {MAP3K7, RNA}, {NFKB1, RNA}, {TRAF6, RNA}, {ICAM1, RNA}, {CFLAR, RNA}, {SYK, RNA}, {MYD88, RNA},
 {LYN, RNA}, {NFKBIA, RNA}, {IL1B, RNA}, {LTBR, RNA}, {CD14, RNA}, {TICAM2, RNA}, {LY96, RNA}}
```

We can visualize any KEGG pathway using `KEGGPathwayVisual`, getting (1) a link to the website, (2) importing the figure (3) importing the figure with highlighted annotations, (4) importing a series of figures with intensities corresponding to each time

point, (5) export a series of figures with time intensities as a movie (animation).

In[58]:= (*1*)KEGGPathwayVisual["path:hsa04064"]

Out[58]= <|Pathway → path:hsa04064, Results → {http://www.kegg.jp/kegg-bin/show_pathway?map=hsa04064}|>

In[59]:= (*2*)KEGGPathwayVisual["path:hsa04064", ResultsFormat → "Figure"]

Out[59]= <|Pathway → path:hsa04064, Results → {

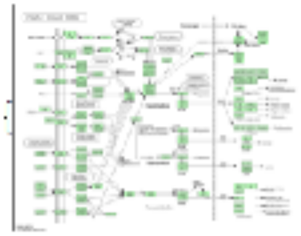

}|>

In[60]:= (*3*)KEGGPathwayVisual["path:hsa04064", ResultsFormat → "Figure", MemberSet → pathwaymembers]

Out[60]= <|Pathway → path:hsa04064, Results → {

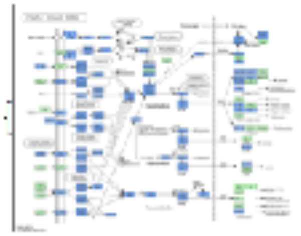

}|>

In[62]:= (*4*)nfkbPathwayFigureList = KEGGPathwayVisual["path:hsa04064", ResultsFormat → "Figure", MemberSet → pathwaymembers, Intensities → Query[Key[#] & /@ pathwaymembers]@rnaFinalTimeSeries]

<|Pathway → path:hsa04064,

Results → {

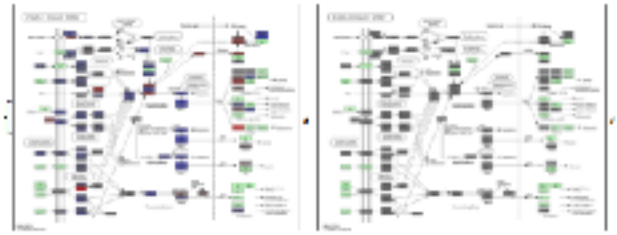

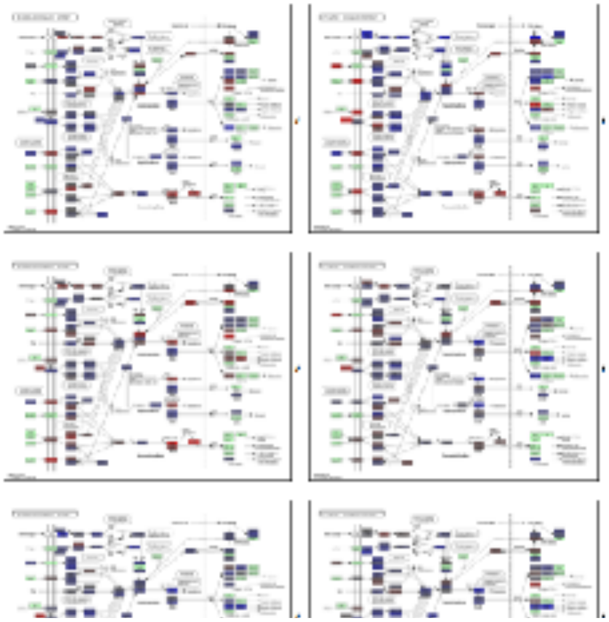

Out[62]=

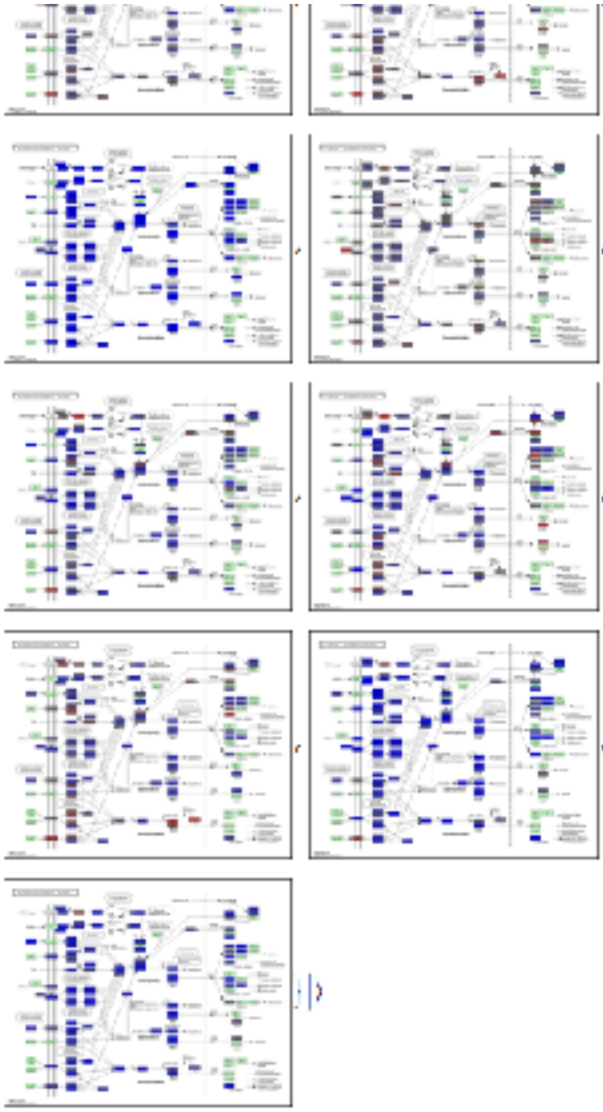

In[83]:= ListAnimate[nfkbPathwayFigureList["Results"], ImageSize → Automatic]

Out[83]=

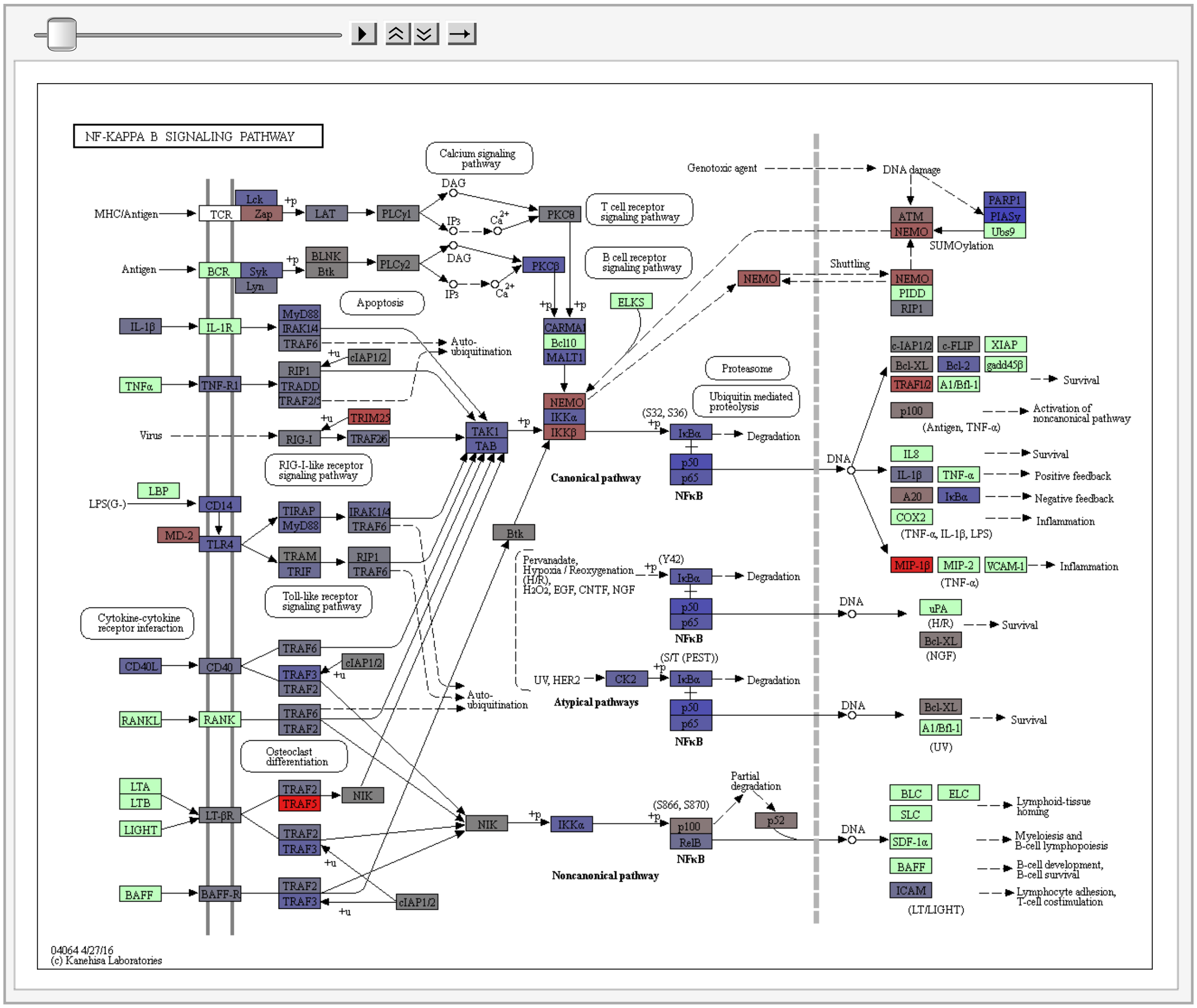

In[72]:=

```
(*5*)KEGGPathwayVisual["path:hsa04064", ResultsFormat → "Movie",
  MemberSet → pathwaymembers, Intensities → Query[Key[#]] & /@ pathwaymembers]@rnaFinalTimeSeries]
```

Out[72]= ⟨|Pathway → path:hsa04064, Results → path_hsa04064.mov|⟩

Appendix: All Commands Up to Enrichment Analysis in One Step

As a summary, we list here all the commands up to the enrichment analysis is one step:

```
In[1]:= << MathIOmica`;
rnaExample = Get[FileNameJoin[{ConstantMathIOmicaExamplesDirectory, "rnaExample"}]];
sampleToDays =
  <|"7" → "186", "8" → "255", "9" → "289", "10" → "290", "11" → "292", "12" → "294", "13" → "297", "14" → "301",
   "15" → "307", "16" → "311", "17" → "322", "18" → "329", "19" → "369", "20" → "380", "21" → "400"|>;
rnaLongitudinal = KeyMap[sampleToDays, rnaExample];
rnaQuantileNormed = QuantileNormalization[rnaLongitudinal];
rnaZeroTagged = LowValueTag[rnaQuantileNormed, 0];
rnaNoiseAdjusted = LowValueTag[rnaZeroTagged, 1, ValueReplacement → 1];
rnaFiltered = FilterMissing[rnaNoiseAdjusted, 3/4, Reference → "255", ShowPlots → False];
timesRNA = TimeExtractor[rnaFiltered];
timeSeriesRNA = CreateTimeSeries[rnaFiltered];
timeSeriesRNALog = SeriesApplier[Log, timeSeriesRNA];
rnaCompared = SeriesInternalCompare[timeSeriesRNALog, ComparisonIndex → 2];
normedRNACompared = SeriesApplier[Normalize, rnaCompared];
rnaFinalTimeSeries = ConstantSeriesClean[normedRNACompared];
(*Bootstrap*)
rnaBootstrap = BootstrapGeneral[rnaLongitudinal, 100 000];
(*1*)rnaBootstrapQuantileNormed = QuantileNormalization[rnaBootstrap];
(*2*)rnaBootstrapZeroTagged = LowValueTag[rnaBootstrapQuantileNormed, 0];
(*3*) rnaBootstrapNoiseAdjusted = LowValueTag[rnaBootstrapZeroTagged, 1, ValueReplacement → 1];
(*4*)
rnaBootstrapFiltered = FilterMissing[rnaBootstrapNoiseAdjusted, 3/4, Reference → "255", ShowPlots → False];
(*5*) timeSeriesBootstrapRNA = CreateTimeSeries[rnaBootstrapFiltered];
(*6*) timeSeriesBootstrapRNALog = SeriesApplier[Log, timeSeriesBootstrapRNA];
(*7*)rnaBootstrapCompared = SeriesInternalCompare[timeSeriesBootstrapRNALog, ComparisonIndex → 2];
(*8*)normedBootstrapRNACompared = SeriesApplier[Normalize, rnaBootstrapCompared];
(*9*)rnaBootstrapFinalTimeSeries = ConstantSeriesClean[normedBootstrapRNACompared];
q95RNA = QuantileEstimator[rnaBootstrapFinalTimeSeries, timesRNA];
q95RNASpikes = QuantileEstimator[rnaBootstrapFinalTimeSeries, timesRNA, Method → "Spikes"];
rnaClassification = TimeSeriesClassification[rnaFinalTimeSeries,
   timesRNA, LombScargleCutoff → q95RNA, SpikeCutoffs → q95RNASpikes];
rnaClusters = TimeSeriesClusters[rnaClassification, PrintDendrograms → True];
goAnalysisRNA = GOAnalysis[rnaClusters, OntologyLengthFilter → 3, ReportFilter → 3 ];
keggAnalysisRNA = KEGGAnalysis[rnaClusters, ReportFilter → 2];
EnrichmentReportExport[goAnalysisRNA,
  OutputDirectory → $UserDocumentsDirectory, AppendString → "GOAnalysisRNA"];
EnrichmentReportExport[keggAnalysisRNA, OutputDirectory → $UserDocumentsDirectory,
 AppendString → "KEGGAnalysisRNA"]
TimeSeriesDendrogramsHeatmaps[rnaClusters]
```

Related Tutorials

- MathIOmica Overview
- MathIOmica Tutorial
- MathIOmica Guide
